# Probing Immune Signatures of Conjugated Pattern Recognition Receptor Ligands Identifies Chimeras with Potent Adjuvant and Antitumor Activities

**DOI:** 10.1021/acs.jmedchem.6c00372

**Published:** 2026-05-06

**Authors:** Špela Janež, Samo Guzelj, Veronika Weiss, Marcela Šišić, Ruža Frkanec, Stane Pajk, Lenny Burgmeijer, Bram Slütter, Žiga Jakopin

**Affiliations:** † Faculty of Pharmacy, 63721 University of Ljubljana, Ljubljana SI-1000, Slovenia; ‡ Centre for Research and Knowledge Transfer in Biotechnology, 37631 University of Zagreb, Zagreb 10000, Croatia; § Div. BioTherapeutics, Leiden Academic Centre for Drug Research, 4496 Leiden University, Leiden 2333 CC, The Netherlands

## Abstract

Pattern recognition receptor (PRR) ligands represent a promising class of immunostimulants. Here, we demonstrate that the covalent conjugation of PRR ligands enables coordinated receptor engagement and amplifies immune responses beyond those achievable with unlinked mixtures. We synthesized a focused panel of chimeric PRR ligands comprising defined pairwise agonist combinations targeting selected extracellular and intracellular PRRs. Using phenotypic screening in human peripheral blood mononuclear cells, we identified conjugates that induced distinct cytokine signatures and enhanced cytotoxic immune activity. Among these, chimeric TLR4/TLR7 and TLR7/RIG-I ligands elicited broad innate immune activation in vitro and enhanced antigen-specific immune responses in murine vaccination models. Notably, intratumoral administration of the conjugated TLR4/TLR7 ligand resulted in significant antitumor activity in a syngeneic B16F10 melanoma model. Collectively, these findings establish covalent PRR ligand conjugation as a powerful chemical strategy for modulating innate immune signaling and support the development of conjugated PRR ligands as next-generation vaccine adjuvants and immunotherapeutics.

## Graphical abstract

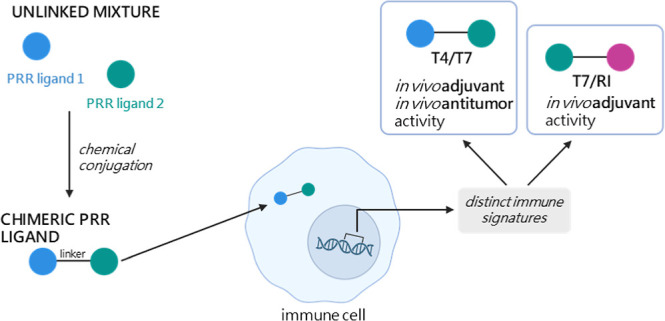

## Introduction

Stimulation of the innate immune system is essential for effective vaccinations and immunotherapies. This is often achieved through adjuvants or immunotherapeutics, molecules that resemble pathogen-associated molecular patterns (PAMPs) and activate pattern recognition receptors (PRRs), such as Toll-like receptors (TLRs), nucleotide-binding oligomerization domain (NOD)-like receptors (NLRs), and retinoic-acid-inducible gene-I (RIG-I)-like receptors (RLRs), present in immune cells, particularly dendritic cells (DCs). On activation, PRRs trigger two innate immune signaling pathways: nuclear factor kappa-light-chain-enhancer of activated B-cells (NF-κB) and interferon regulatory factors (IRF), providing indispensable initial signals that determine the type, magnitude, and durability of adaptive immune response.^1^

Synthetic PRR ligands, in particular those targeting TLR4, TLR7, and TLR9, that are widely explored as vaccine adjuvants or immunotherapeutics, are promising candidates but often elicit suboptimal responses.^1^ In addition, overactivation can trigger systemic inflammation, while repeated stimulation may result in immune tolerance.^2^ During natural infections, pathogens presenting multiple PAMPs engage several innate immune receptors simultaneously. For example, the yellow fever vaccine activates a broad immune response via multiple PRRs, including TLRs 2, 7, 8, and 9.^3^ Coactivation of distinct receptors by agonist mixtures enhances immune signaling via cross-talk and cross-regulation.^4^ Chemical conjugation of such agonists allows coordinated activation of targets within the same cell, potentially producing superior immune responses beyond those of established adjuvant platforms.^5^ Several conjugated dual-TLR ligands, including TLR2/6/TLR9 ^6^ and TLR2/6/TLR7,^7^ as well as conjugated PRR ligands targeting NOD2/TLR2/6,^8^ NOD2/TLR7,^9,10^ and NOD2/TLR4,^11^ have demonstrated synergistic immune activation, regardless of the subcellular localization of their targets.

Understanding immune receptor cross-talk is critical for rational adjuvant and immunotherapeutic design, as effective responses rely on precise coordination. Building on promising results with conjugated NOD2/TLR7 ligands,^9^ we sought to dissect synergistic PRR interactions capable of driving potent immune activation. Unlike prior dual-PRR conjugates that focused primarily on specific receptor pairings, this study provides a systematic evaluation of chemically defined small-molecule PRR conjugates across multiple receptor combinations including previously unexplored pairings. To this end, we synthesized conjugates combining agonists targeting pairs of the following PRRs: extracellular TLR2, TLR4, and intracellular NOD2, TLR7, and RIG-I. These receptors were selected based on their known adjuvant properties^12^ and the ability to enhance nonspecific antitumor activity in the tumor microenvironment.^13,14^ Additionally, synergistic effects—reflected in cytokine secretion and adjuvant activity—have been reported for all pairwise combinations of these receptors.^15,16^ To identify optimal conjugates, we employed a two-step in vitro screening approach. First, we profiled their cytokine responses in human peripheral blood mononuclear cells (PBMCs). Next, we assessed their ability to induce PBMC-mediated cytotoxicity against cancer cells. Top candidates were tested for ex vivo adjuvant activity in murine bone-marrow-derived dendritic cells (BMDCs). Finally, we identified potent chimeric combinations and conjugated TLR4/TLR7 and TLR7/RIG-I ligands that functioned as effective in vivo vaccine adjuvants. Notably, the TLR4/TLR7 ligand also induced robust antitumor response in a B16F10 melanoma model.

## Results and Discussion

At the outset of this study, our aim was not to pursue iterative medicinal chemistry optimization of individual PRR agonists but rather to systematically examine how covalent conjugation of chemically and biologically distinct PRR ligands reshapes innate immune activation relative to unlinked agonist mixtures. By treating each agonist as a modular signaling unit and enforcing their codelivery through covalent linkage, we sought to probe how agonist pairing and coordinated intracellular trafficking influence immune outcomes. Such an approach provides a complementary perspective to classical structure–activity relationship (SAR)-driven optimization and is particularly suited to interrogating complex immunological systems.

### Design and Construction of Conjugated PRR Ligands

Building on the immunological rationale outlined above, we designed a chemically defined set of chimeric PRR ligands in which two distinct small-molecule agonists are covalently linked to a single construct. The goal was to assemble a focused and synthetically tractable panel that enables the systematic interrogation of pairwise PRR cross-talk within a common molecular framework. Five PRR agonists were selected as building blocks: **N2** (NOD2 agonist), **T1/2** (TLR1/2 agonist), **T4** (TLR4 agonist), **T7** (TLR7 agonist), and **RI** (RIG-I agonist) (Figure S1). These ligands were chosen based on their established biological activity, structural simplicity, and suitability for chemical modification. For NOD2 activation, we used **N2**, a highly potent agonist previously identified by our group.^17^ While TLR2/6 agonists have been conjugated with NOD2 agonists in prior studies,^8,18^ their association with systemic inflammation^19^ prompted us to instead employ the small-molecule TLR1/2 agonist **T1/2**, which signals through the TLR1/2 heterodimer and exhibits favorable adjuvant properties.^20^ For TLR4 activation, we employed the carboxylic acid precursor **T4** of the pyrimido­[5,4-*b*]­indole agonist **T4′**.^21^ Although **T4** is only a weak agonist per se, its chemical properties make it well-suited for conjugation as shown previously.^22^ Similarly, the purine-based TLR7 agonist **T7** was chosen based on previous reports demonstrating its suitability for conjugation.^23^ For RIG-I activation, we selected **RI**, the active form of the RIG-I agonist **RI′**.^24^ All of the selected ligands feature functional groups that permit chemical derivatization. In principle, pairwise conjugation of five ligands affords ten unique combinations. However, the present panel was intentionally limited to nine members, excluding the previously reported conjugated NOD2/TLR7 ligand **N2/T7**.^9^ This combination has been characterized in detail by our own group and was therefore omitted to avoid redundancy and focus on unexplored PRR agonist pairings. The resulting conjugate set comprises **T4/N2**, **N2/RI**, **T1/2/N2**, **T1/2/T4**, **T1/2/T7**, **T1/2/RI**, **T4/T7**, **T4/RI**, and **T7/RI** (Figure 1).

**Figure 1:**
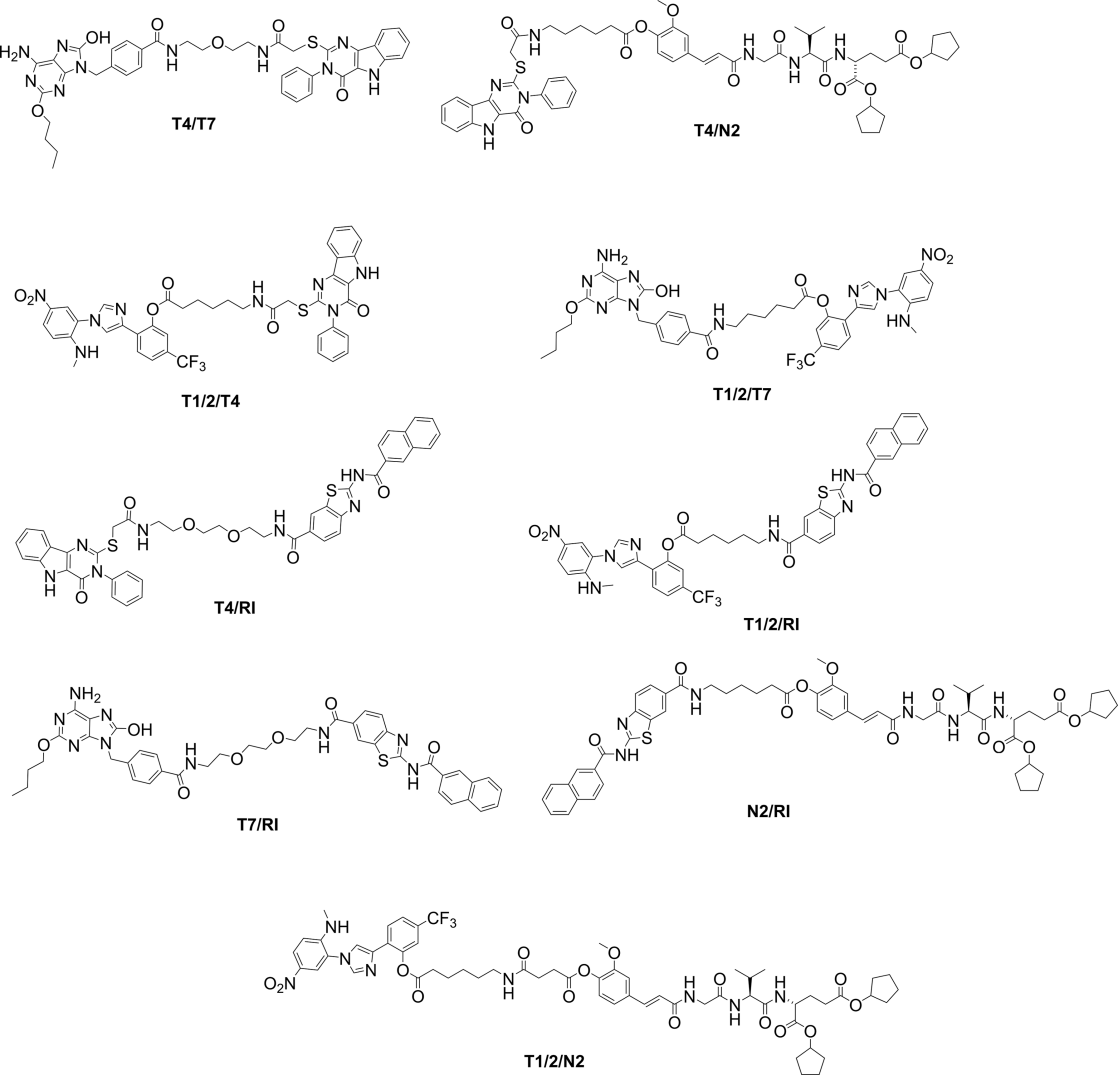
Chemical structures of the synthesized chimeric PRR ligands.

From a design perspective, the conjugates were conceived to balance chemical stability with biological functionality. While intracellular cleavage is expected and, in many cases, desirable to liberate individual agonists, the constructs were designed such that productive receptor engagement could also occur in the absence of complete processing. To this end, ligands were connected through either ester or amide linkages, providing differential and tunable stability toward enzymatic hydrolysis. Ester-linked conjugates are expected to undergo more efficient cleavage in cellular systems, whereas amide-linked constructs were designed to exhibit increased stability. Accordingly, linker length and attachment geometry were selected to preserve receptor compatibility both prior to and following potential intracellular processing. The synthetic realization of these conjugates is described in the following section.

### Chemistry

Guided by the design principles outlined above, we synthesized a focused panel of nine covalent PRR ligand conjugates comprising pairwise combinations of five chemically accessible agonists (**N2**, **T1/2**, **T4**, **T7**, and **RI**). Given the limited number of orthogonal PRR ligands that combine robust biological activity with suitable functional handles for derivatization, this set represents a systematic exploration of feasible conjugation topologies. The overall synthetic strategy relied on stepwise assembly from prefunctionalized agonist intermediates using either 6-aminohexanoic acid-based or poly­(2-aminoethyl)­ether linkers. The preparation of protected linker building blocks is described in the Supporting Information (Figure S1), whereas the final conjugation steps are detailed below and summarized in Scheme 1 (for conjugates **T4/T7**, **T7/RI**, and **T4/N2**), Scheme 2 (for conjugates **T1/2/T4**, **T1/2/T7**, and **T1/2/N2**), and Scheme 3 (for conjugates **T4/RI**, **T1/2/RI**, and **N2/RI**).

**Scheme 1:**
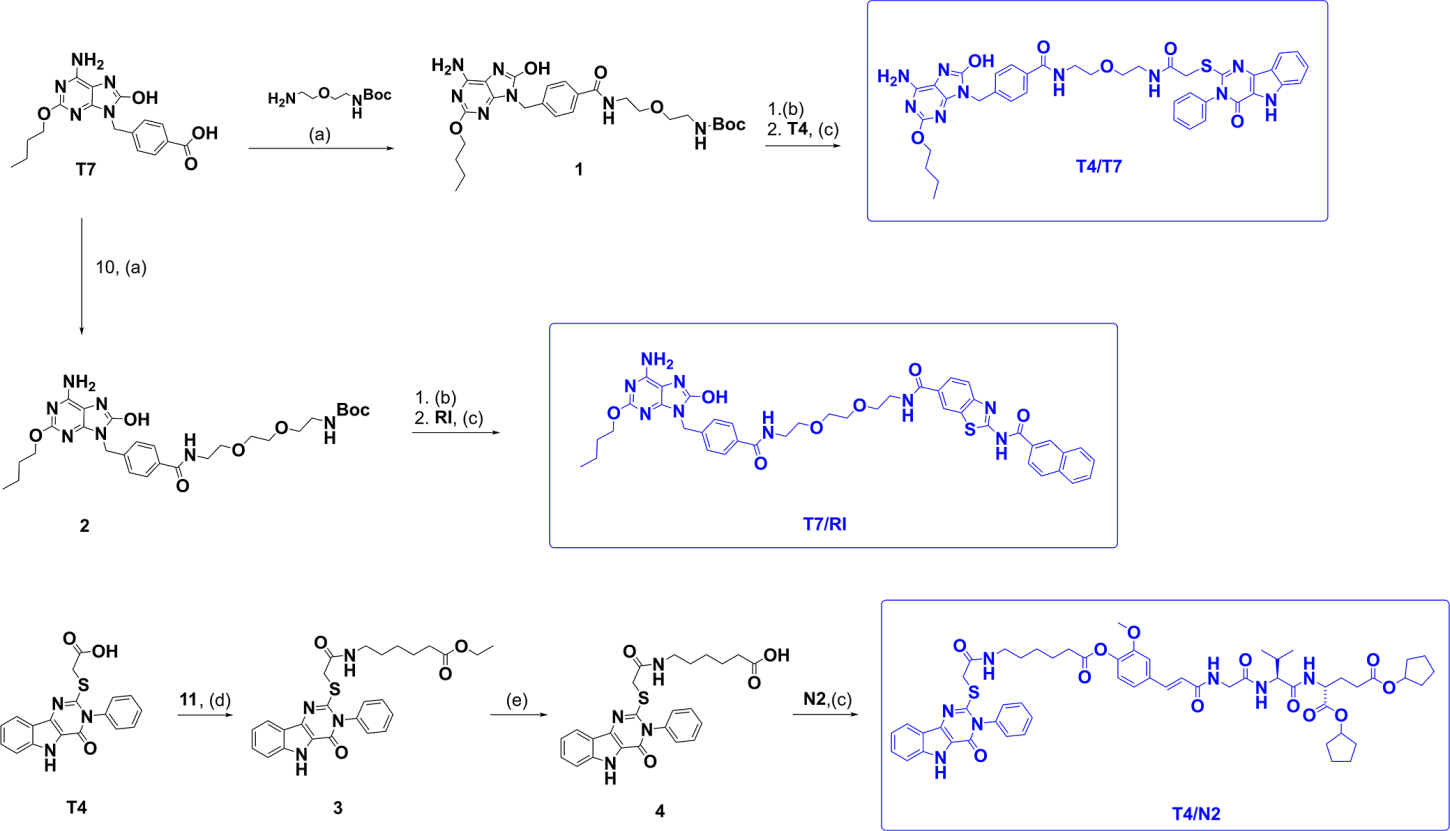
Synthesis of Conjugates **T4/T7**, **T7/RI,** and **T4/N2** ^a^

a Reagents and conditions: (a) DIPEA, COMU, DCM, DMSO, rt.; (b) TFA, DCM (1:5), rt.; (c) DIPEA, COMU, DMF, rt.; (d) DIPEA, HATU, DMF, rt.; (e) NaOH, MeOH, rt.

**Scheme 2:**
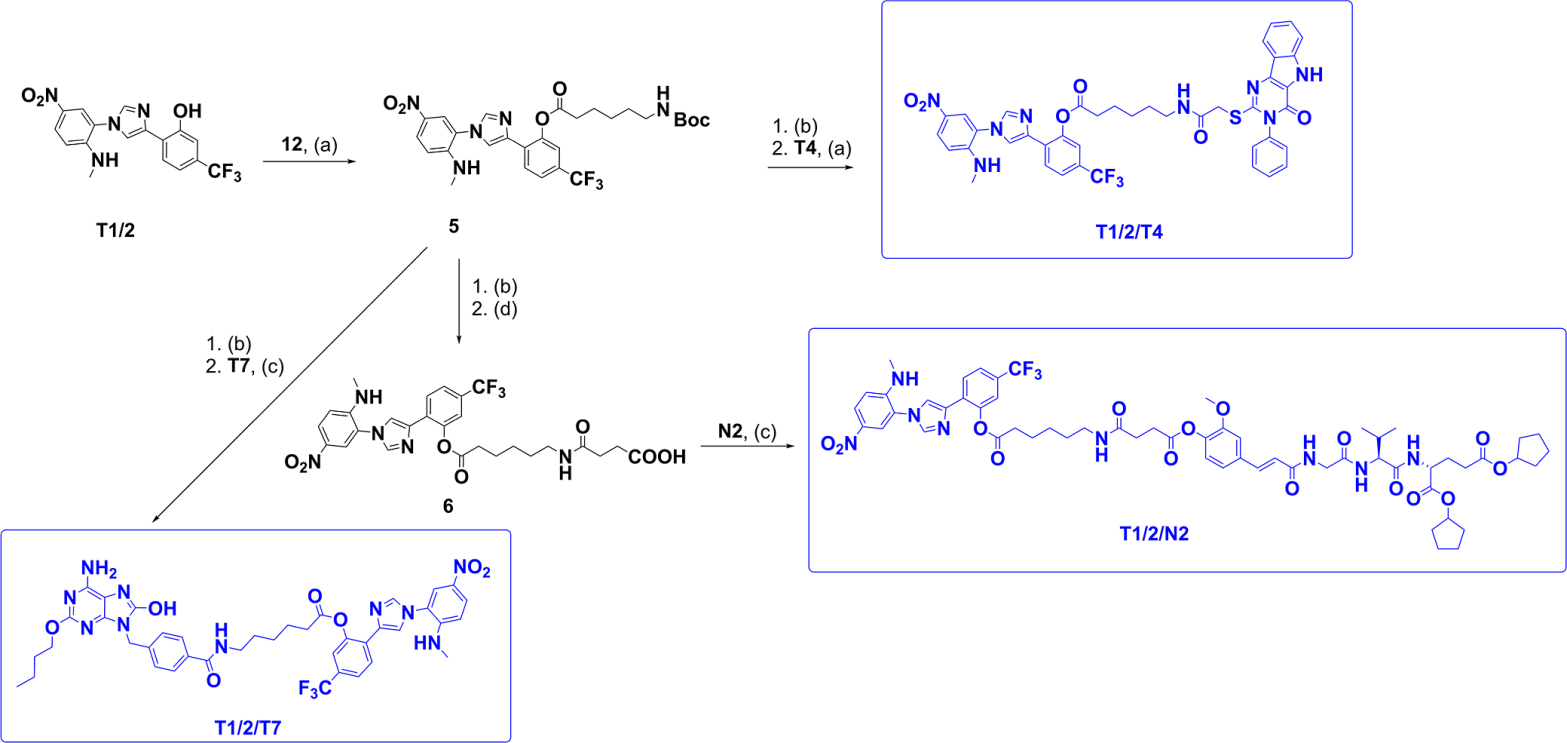
Synthesis of Conjugates **T1/2/T4**, **T1/2/T7,** and **T1/2/N2** ^a^

a Reagents and conditions: (a) DIPEA, HATU, DMF, rt.; (b) TFA, DCM (1:5), rt.; (c) DIPEA, COMU, DMF, rt.; (d) succinic anhydride, Et_3_N, DMAP, DMF, rt.

**Scheme 3:**
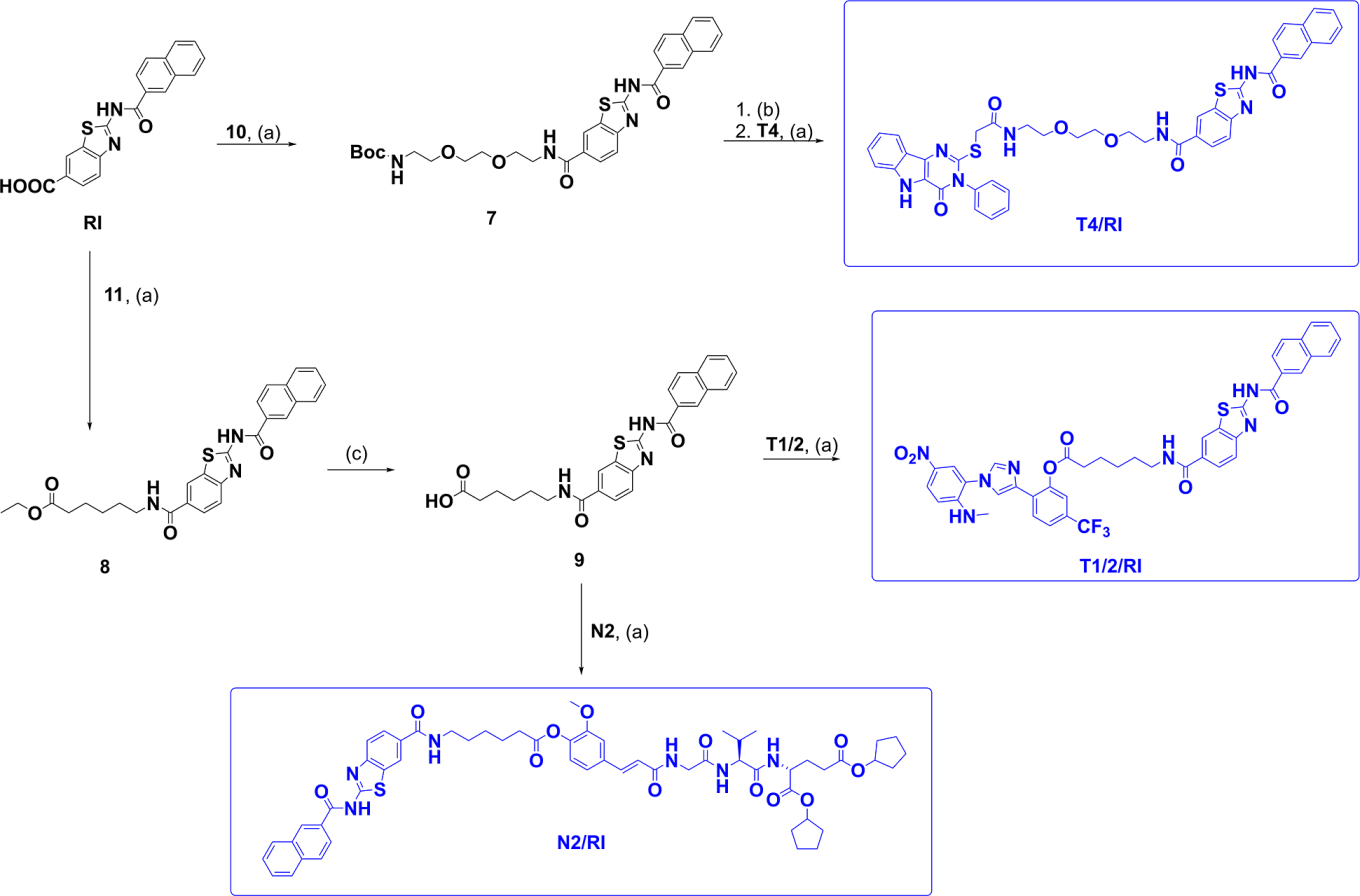
Synthesis of Conjugates **T4/RI**, **T1/2/RI,** and **N2/RI** ^a^

a Reagents and conditions: (a) DIPEA, COMU, DMF, rt.; (b) TFA, DCM (1:5), rt.; (c) NaOH, MeOH, rt.

With all agonist intermediates available, conjugate assembly commenced with COMU-mediated amide bond formation between the TLR7 agonist **T7** and mono-Boc-protected bis­(2-aminoethyl)­ether, affording intermediate **1**. Subsequent acidolytic removal of the Boc group liberated a primary amine, which was directly coupled to the carboxylic-acid-containing TLR4 agonist precursor **T4** to yield conjugate **T4/T7**. An analogous strategy was employed for the synthesis of **T7/RI**. In this case, **T7** was first coupled to the corresponding mono-Boc-protected linker **10** to give intermediate **2**. After Boc deprotection, the resulting amine was coupled to RIG-I agonist **RI**, furnishing the desired conjugate.

For the preparation of **T4/N2**, the sequence was reversed to accommodate the functional groups of the NOD2 agonist. **T4** was initially reacted with ethyl 6-aminohexanoate (**11**), providing intermediate **3**. Base-catalyzed hydrolysis of the ethyl ester afforded carboxylic acid **4**, which was subsequently coupled to **N2** to generate the final conjugate.

The synthesis of conjugates incorporating the TLR1/2 agonist **T1/2** exploited its phenolic hydroxyl group as the linkage site. Acylation with Boc-protected 6-aminohexanoic acid **12** afforded intermediate **5**, introducing a masked amine handle for further diversification. Acidolytic Boc removal then provided a common amine intermediate that served as a branching point for multiple conjugates. Coupling of this amine with **T4** under HATU-mediated conditions yielded conjugate **T1/2/T4**, whereas the reaction with **T7** furnished **T1/2/T7**. In contrast, the assembly of **T1/2/N2** required an additional functional group interconversion; the free amine was first acylated with succinic anhydride to generate intermediate **6**, which upon COMU-mediated coupling with **N2** provided the final conjugate.

Conjugates incorporating RIG-I agonist **RI** were accessed through two complementary routes, depending on the identity of the second PRR ligand. In the first approach, **RI** was coupled to mono-Boc-protected linker **10** to give intermediate **7**. Following acidolytic deprotection, the liberated amine was coupled to **T4**, affording conjugate **T4/RI**. Alternatively, RI was reacted with ethyl 6-aminohexanoate (**11**) to form intermediate **8**. Hydrolysis of the ethyl ester yielded carboxylic acid **9**, which could be coupled directly to either **T1/2** or **N2**, producing conjugates **T1/2/RI** and **N2/RI**, respectively.

### Receptor-Specific Activities Exhibited by Conjugated PRR Ligands

Individual PRR ligand building blocks as well as the corresponding conjugated ligands were first evaluated for receptor-specific activities using commercially available HEK-Blue reporter cell lines (and HEK-Lucia cells for RIG-I agonists) together with appropriate positive controls (Figure 2). This initial profiling was performed at a single concentration to qualitatively assess whether covalent conjugation preserves the ability of each pharmacophore to engage its cognate receptor, followed by dose–response analyses where meaningful receptor activation was observed.

**Figure 2:**
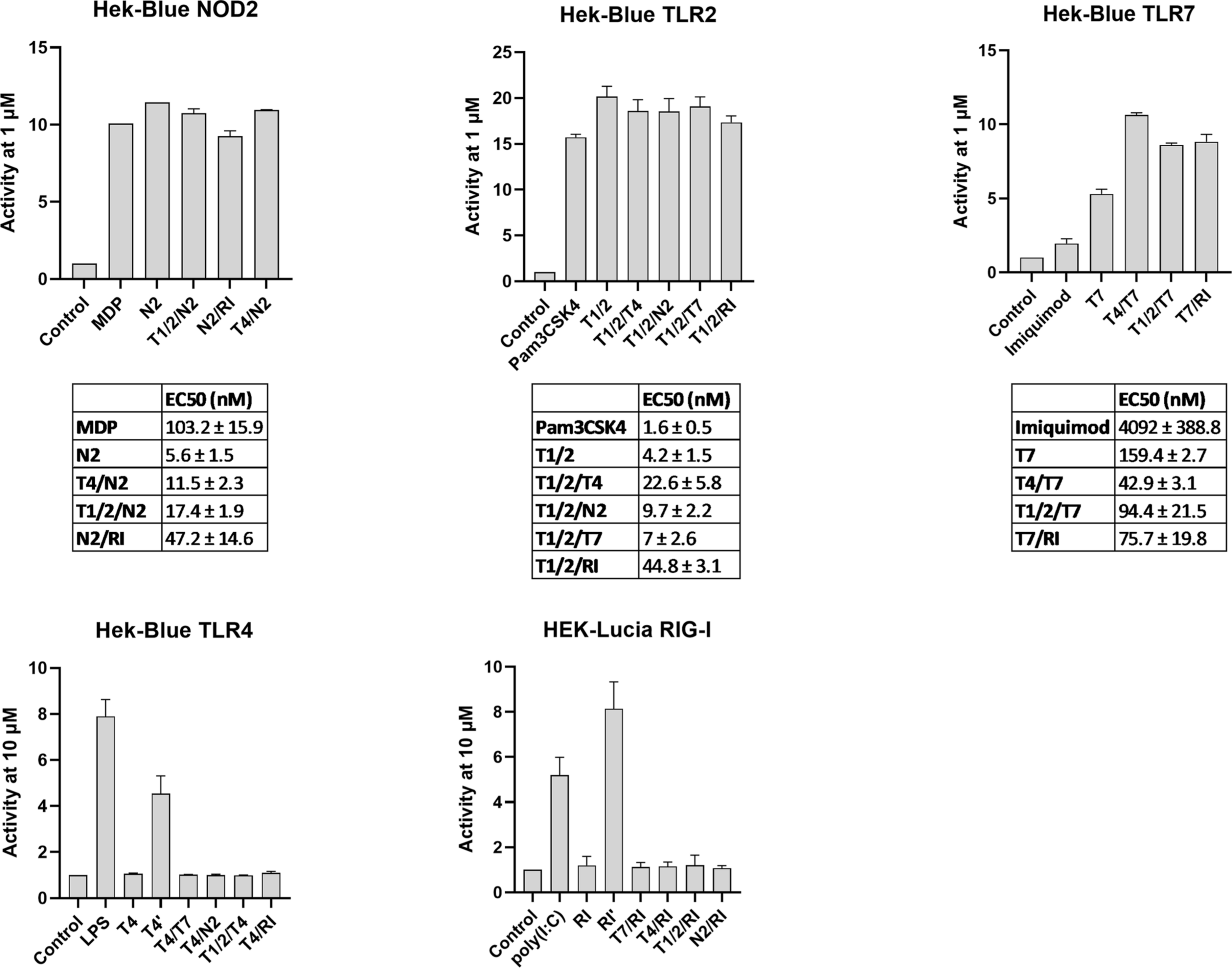
Receptor-specific activities of individual PRR ligands and conjugated PRR ligands. HEK-Blue NOD2, TLR2, TLR7, TLR4, or HEK-Lucia RIG-I cells were treated with the compounds (1 or 10 μM as indicated) and the corresponding positive controls (1 μM MDP for NOD2, 100 nM Pam3CSK4 for TLR2, 1 μM imiquimod for TLR7, 100 ng/mL LPS for TLR4, and 100 ng/mL Poly­(I/C) for RIG-I) for 18 h. The activities are shown relative to the vehicle-treated control (0.1% DMSO). Data are mean ± SEM of three independent experiments. EC_50_ values were determined in HEK-Blue NOD2, TLR2, and TLR7 cells in at least three independent experiments with eight concentrations (1 nM to 10 μM for NOD2 cells, and 0.1 nM to 1 μM for TLR2 and TLR7 cells).

At a concentration of 1 μM, the parent agonists **N2**, **T1/2**, and **T7** robustly activated their respective receptors, whereas **T4** and **RI** did not elicit detectable receptor activation even at 10-fold higher concentration. Consistent with previous reports, dose–response analysis of the TLR4 agonist **T4′** (EC_50_ = 4.3 μM) and its carboxylic acid precursor **T4** (EC_50_ = 90 μM) revealed moderate-to-weak intrinsic agonistic activity. In the case of **RI**, esterification (as present in the prodrug **RI′**; EC_50_ = 7.5 μM in the HEK-Lucia assay) is required to confer cell permeability, while the active RIG-I-binding species is the corresponding carboxylic acid. HEK-Lucia cells are capable of rapidly converting esterified **RI′** to the active acid form, enabling the assessment of RIG-I activation in this system.

Overall, conjugated PRR ligands retained the ability to activate their intended receptors, indicating that the covalent linkage does not compromise receptor engagement. All conjugates containing the NOD2 agonist **N2** activated NOD2 signaling, with EC_50_ values in the low nanomolar range, preserving the potency of the parent ligand. A similar trend was observed for **T1/2** and **T1/2**-containing conjugates, which likewise exhibited robust activity in the low nanomolar range. Notably, conjugates incorporating the TLR7 agonist displayed low- to midnanomolar potency and, in all cases, showed increased activity relative to unconjugated **T7**.

In contrast, conjugates incorporating the TLR4 or RIG-I ligands did not permit reliable EC_50_ determination in the corresponding reporter assays under these conditions, reflecting the intrinsically weak agonistic activity of **T4** and the limited intracellular hydrolysis of **RI**-containing conjugates under these assay conditions. Namely, even at the highest concentrations tested, the **T4**-featuring conjugates did not reach the maximal levels of receptor activation observed with the canonical TLR4 agonist LPS. Analogously, maximal responses of RIG-I-targeting conjugates remained below those observed for poly­(I/C). While these data indicate low apparent potency and submaximal efficacy in HEK-based reporter assays, they do not preclude functional immune activity in physiologically relevant systems, particularly in primary immune cells or in vivo settings, where cellular uptake and enzymatic processing differ substantially from engineered reporter systems.

While a comprehensive analysis of receptor-proximal signaling kinetics and cell type-specific pathway activation would provide additional mechanistic insights, such studies were beyond the scope of the present work. Instead, receptor engagement and pathway specificity were established using a combination of complementary approaches, including receptor-specific reporter assays, pharmacological antagonism, transcriptomic profiling, and functional immune readouts, providing a level of validation appropriate for a chemically focused investigation.

### Conjugated PRR Ligands Induce Cytokine Release from PBMCs

The immunostimulatory potential of conjugates, single agonists, and unlinked agonist mixtures was first assessed in human PBMCs, rather than in commonly used reporter cell lines. The latter express only select PRRs, lack metabolic activity, and thus carry a high risk of false negatives.^25^ More importantly, they do not reflect the complexity of primary immune responses. PBMCs, by contrast, offer a heterogeneous mix of immune cells and a physiologically relevant system to study PRR coactivation, capturing multi-input effects such as paracrine cytokine signaling. Therefore, their use has recently been proposed for the discovery of novel immunostimulants.^26^ Compounds were tested at 1 μM, the concentration previously yielding clear distinctions among conjugated NOD2/TLR7 ligands.^9^ Conjugate **N2/T7** ^9^ and LPS served as internal and external controls.

Our primary objective was to determine how chimeric conjugates shape cytokine responses. Recognizing that no single in vitro parameter reliably predicts in vivo adjuvanticity, we employed multiplex cytokine assays to profile the breadth and intensity of the responses. Cytokine fingerprints also inform on the type of immune response triggered. Accordingly, we analyzed supernatants for 13 cytokines and chemokines associated with Th1, Th2, and Th17 responses (Figures 3A, S2A, Table S1). Single agonists or coadministration of individual ligands led to modest increases in cytokine levels, whereas certain chimeric molecules showed clear synergy. Notably, **T4/T7** and **T7/RI** induced significantly higher cytokine secretion than their respective unlinked mixtures, confirming that covalent conjugation enhances immune activation. Both conjugates elicited strong Th1 responses, with marked increases in interleukin (IL)-12p70, interferon (IFN)-γ, and IFN-γ-induced protein 10 (IP-10), with **T7/RI** showing slightly stronger effects. These cytokines are hallmarks of IRF pathway activation and cellular immunity. IL-12p70 promotes natural killer (NK) cell activation, IFN-γ production, and Th1/cytotoxic T cell polarization, while IP-10 recruits T cells and DCs. Although proinflammatory responses were less pronounced, both conjugates elevated IL-6, transforming growth factor (TGF)-β1, and monocyte chemoattractant protein 1 (MCP-1), with minimal effects on other cytokines. Interestingly, a previously reported TLR4/TLR7 agonist based on a triazine scaffold failed to exhibit synergy in NF-κB activation or cytokine output from BMDCs.^22^ Conjugate **T1/2/T7** also displayed synergy, albeit weaker, inducing IL-12p70 and upregulating the type 2-associated cytokines IL-4 and TGF-β1. Increases in IL-10 and IL-17A were observed but not statistically significant. Importantly, the levels of endogenous pyrogen tumor necrosis factor (TNF)-α and IL-1β, indicators of systemic inflammation and inflammasome activation, were not significantly elevated following stimulation with conjugates **T4/T7**, **T7/RI**, and **T1/2/T7**, suggesting a moderate systemic response. In contrast, conjugates **T4/T7**, **T7/RI**, **T1/2/T7**, and **T1/2/N2** induced substantial IL-6 secretion. While IL-6 is often linked to inflammation, it also stimulates B cell antibody production and influences Th17, Tfh, and Treg cell differentiation.^27^

**Figure 3:**
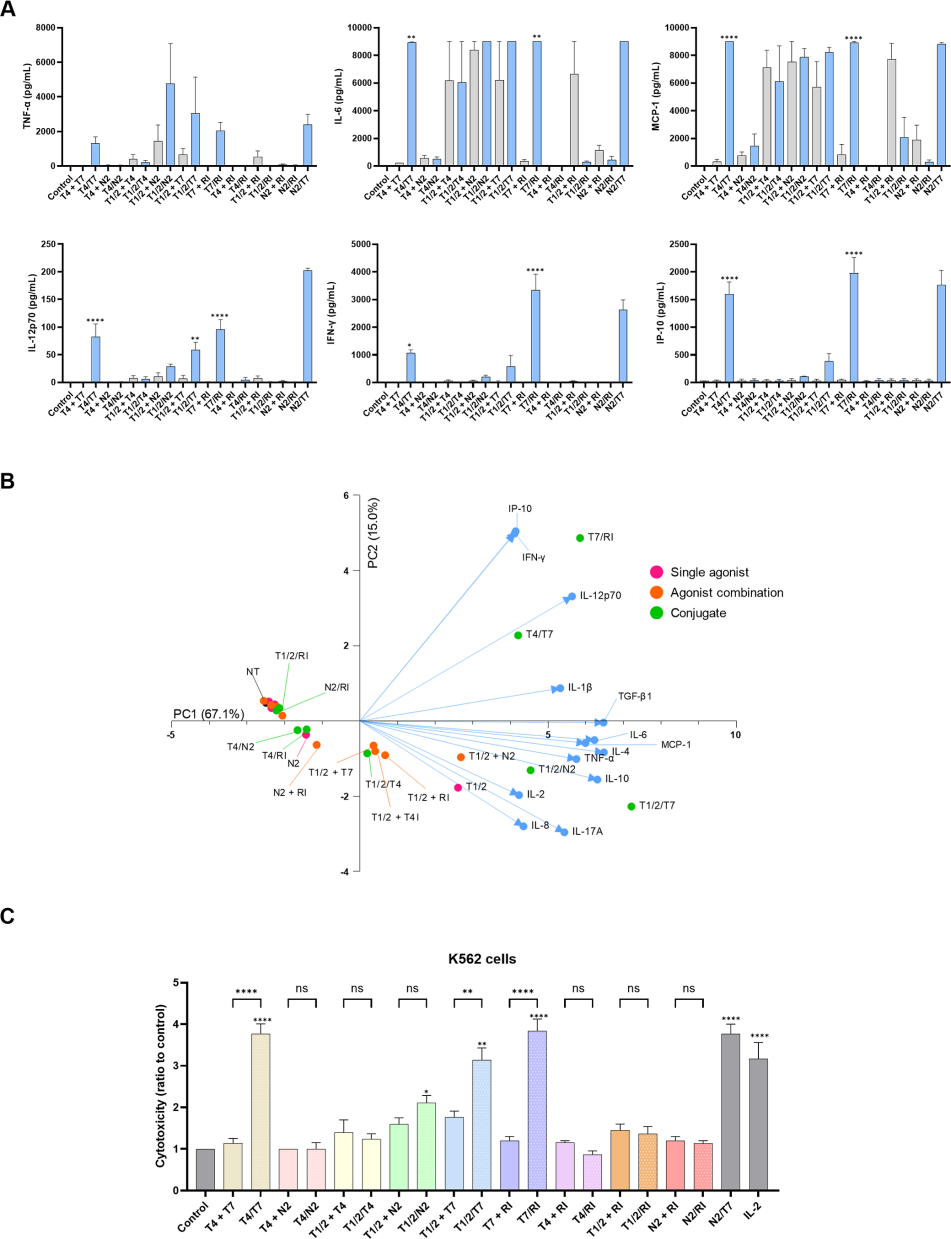
(A) Cytokine release from human PBMCs after 18 h stimulation with conjugated agonists (blue bars), unlinked mixtures (gray bars, both 1 μM), **N2/T7** (1 μM, positive control), or vehicle (0.1% DMSO). Data are mean ± SEM of three independent experiments. One-way ANOVA with Bonferroni’s test compared unlinked mixtures to conjugates. *, *p* < 0.05; **, *p* < 0.01; ***, *p* < 0.001; ****, *p* < 0.0001. (B) Principal component analysis (PCA) of cytokine/chemokine levels in PBMC supernatants after stimulation with conjugates, single agonists, and unlinked mixtures. PCA includes data from three donors. PC scores for stimulation conditions (red, orange, green) and cytokine loadings (blue) are shown. PCA captures 82.1% of variance (PC1:67.1%, PC2:15.0%). (C) PBMC cytotoxicity against K562 cells after 18 h treatment with conjugates, unlinked mixtures (1 μM), controls (**N2/T7**, 1 μM; IL-2, 200 U/mL), or vehicle (0.1% DMSO). Cytotoxicity was assessed after 4 h coincubation. Data are relative to the negative control (NT, 0.1% DMSO) and shown as mean ± SEM of three independent experiments. ANOVA with Bonferroni’s test compared both unlinked mixtures to conjugates and all compounds versus control; ns, not significant; *, *p* < 0.05; **, *p* < 0.01; ***, *p* < 0.001; ****, *p* < 0.0001.

The remaining conjugates failed to markedly enhance individual cytokine levels, relative to unlinked mixtures. For example, **T4/N2** lacked activity, in contrast to Ding et al., who reported enhanced IL-6 secretion and antigen-presenting cell maturation by conjugated NOD2/TLR4 agonists, leading to improved antibody and T cell responses.^11^ It is worth noting that NOD2 is known to modulate TLR4 signaling—either enhancing or suppressing cytokine production depending on context.^28^ **N2/RI** was also inactive, unlike the dual NOD2/RIG-I agonist dinucleotide SB9200, which improved BCG vaccine efficacy.^29^ The inactivity of **T1/2/T4**, **T1/2/RI**, and **T4/RI** aligns with previous findings showing no synergy among TLR2, TLR4, and RIG-I agonists on T cell proliferation assays using BMDC/T cell cocultures.^30^ Of note, the activity of innate immune agonists does not always correlate directly with the cytokine levels in PBMCs. Instead, it reflects a complex interplay of multiple secreted cytokines and paracrine signaling.^31^

To better understand the architecture of these responses, we performed PCA of cytokine distributions from conjugates, unlinked mixtures, and single agonists across 13 variables. PCA revealed stimulus-specific clustering, with PC1 and PC2 explaining 82% of the total variance. Contributions of individual cytokines to each axis are listed in Table S2A. PC1 was driven by a core innate and adaptive immune signature, while PC2 differentiated response types: positive PC2 values were associated with IP-10, IFN-γ, and IL-12p70, whereas negative PC2 values corresponded to IL-2, IL-8, and IL-17A. As shown in Figure 3B and Table S2B, most conjugates had distinct PCA profiles. **T7/RI** and **T4/T7** strongly induced IFN-γ, IL-12p70, and IP-10 and also upregulated PC1-group cytokines. In contrast, **T1/2/N2** and especially **T1/2/T7** induced both PC1 group and negative PC2-associated cytokines such as IL-2 and IL-17A. To prioritize compounds with the most pronounced impact, we defined a selection criterion using a three-unit PCA radius centered around an untreated control. Compounds positioned outside this boundary—**T4/T7**, **T7/RI**, **T1/2/T7** and **T1/2/N2**—exhibited the most distinctive immune signatures, with **T1/2/T7** and **T1/2/N2** showing particularly unique profiles.

### Conjugated PRR Ligands Enhance PBMC Cytotoxicity against Cancer Cells

To evaluate the antitumor immune potential, we used a PBMC cytotoxicity assay against K562 cells. We tested single agonists (Figure S2B), unlinked mixtures, and conjugates (Figure 3C, Table S3). Unlike isolated NK cells, PBMCs preserve contributions from accessory immune cells that interact via cytokine signaling upon PRR stimulation. **T4/T7** and **T7/RI** significantly enhanced cytolytic activity, mirroring their strong cytokine induction. **T1/2/T7** showed a milder but notable effect, while **T1/2/N2** did not reach statistical significance. Neither single agonists nor unlinked mixtures increased the cytotoxicity, highlighting the superior potency of chimeric conjugates. Independent treatment of PBMCs and cancer cells showed no direct cytotoxicity (Table S3), confirming that the effect stems from immune activation. Notably, the same conjugates that performed best in cytokine assays also emerged as the top candidates here. Based on this dual-screening strategy, we selected these conjugates for further characterization.

### RNA Sequencing Reveals Differential Gene Expression in Conjugate-Stimulated PBMCs

To explore transcriptional changes, PBMCs were stimulated overnight with selected conjugates, followed by mRNA isolation and sequencing; vehicle-treated cells served as controls. Differential expression analysis revealed that chimeric ligands **T4/T7**, **T7/RI**, **T1/2/T7,** and **T1/2/N2** significantly upregulated the transcription of 1,719, 1,484, 1,051, and 541 genes, respectively, while downregulating 1,538, 1,443, 854, and 629 genes (Figure 4A). Pathway enrichment analysis of differentially expressed genes using the KEGG database identified several innate immune-related pathways. All conjugates enriched the “cytokine–cytokine receptor interaction”, “viral protein interaction with cytokine and cytokine receptor”, and “hematopoietic cell lineage” pathways. **T1/2/N2** uniquely enriched the “IL-17 signaling pathway” (Figure 4B). We categorized synergized transcripts into four clusters (Figure 4C,D): (i) Th1-associated genes, (ii) Th2-linked genes, (iii) genes defining the Th17 secretory phenotype, and (iv) other immune-related transcripts.

**Figure 4:**
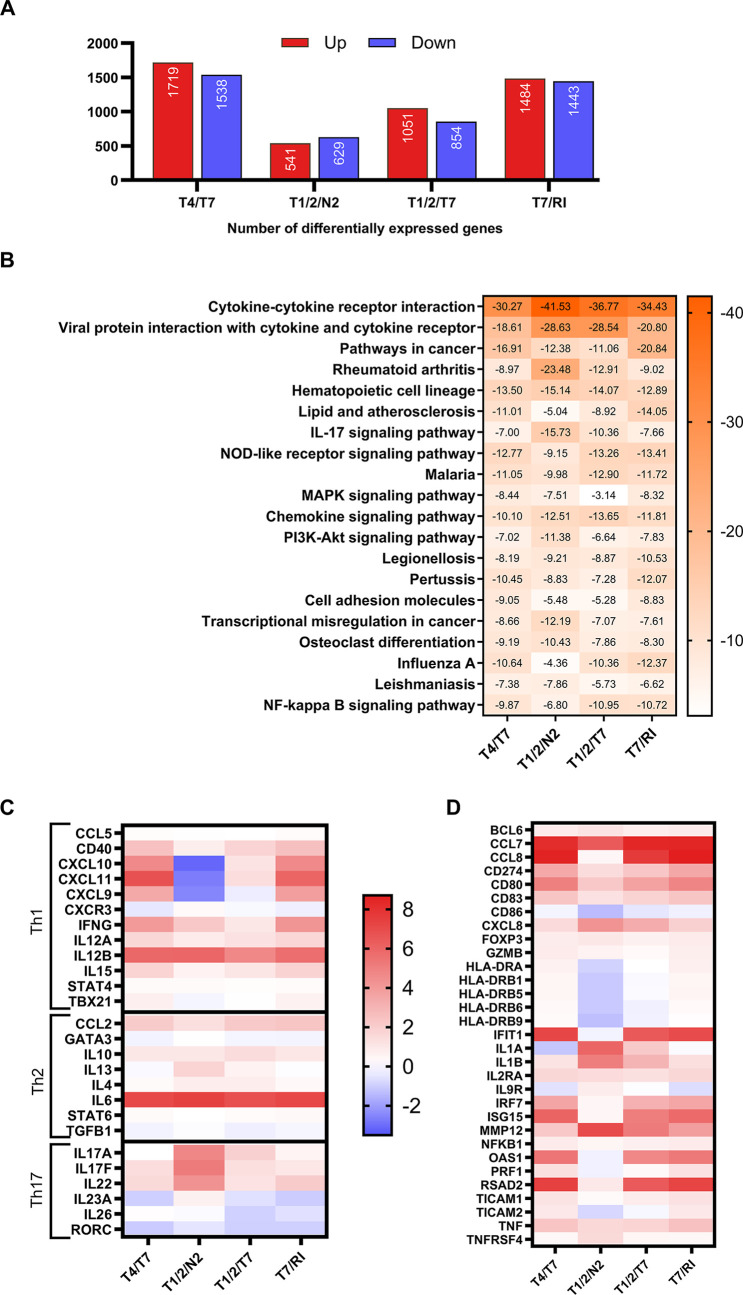
(A) Number of significantly upregulated/downregulated genes in PBMCs from three donors after 18 h treatment with conjugates (1 μM), using a false discovery rate <0.1 and fold-change >2 or <0.5 vs vehicle (0.1% DMSO), as determined by DESeq2 analysis using an adjusted *p*-value. (B) Heatmap of 20 top enriched KEGG terms colored by log­(*q* value). Differentially expressed genes from Figure 3A were used as the input for pathway enrichment analysis by Metascape. (C,D) Transcriptomic profiles of conjugate-stimulated PBMCs. Heatmaps show log_2_(fold change) in gene expression relative to unstimulated control: (C) Th1, Th2, and Th17 response genes; (D) other relevant differentially expressed genes. Red indicates upregulation, blue downregulation.

In line with the results obtained at the protein level, **T4/T7** and **T7/RI**, and to a lesser extent **T1/2/T7** and **T1/2/N2**, enriched the cytokine signaling genes. Both **T4/T7** and **T7/RI** strongly induced the transcription of IFN-γ (*IFNG*) and both IL-12 subunits (*IL12A*, *IL12B*), hallmark Th1 cytokines, alongside *IL15*, which is also associated with Th1 immunity. This polarization was reinforced by the upregulation of the T-bet transcription factor (*TBX21*), the master regulator of Th1 cell differentiation, as well as IFN-γ-inducible chemokines *CXCL9*, *CXCL10*, and *CXCL11*, which act through the CXCR3 receptor expressed on Th1 and NK cells. Importantly, genes for granzyme B (*GZMB*) and perforin (*PRF1*), indicative of cytolytic activity,^32^ were also markedly upregulated. Moderate increases in Th17-associated genes (*IL17F*, *IL22*) were seen, whereas Th2 markers (*IL4*, *IL13*, *STAT6*, and *GATA3*) remained largely unchanged, with minor changes in *CCL2*, *IL6*, and *IL10*, which promote Th2 differentiation. **T4/T7** and **T7/RI** also upregulated genes involved in IFN-signaling (*IFIT1*, *IRF7*, *ISG15*, *OAS1*, *RSAD2*) and chemokine recruitment (*CCL7*, *CCL8*), further supporting their role in activating Th1-biased immune responses, as well as DC maturation markers (*CD40*, *CD80*, *CD83*, and *HLA-DR*). **T1/2/N2** had little effect on Th1 genes beyond modest increases in *IL12B* and *IFNG*, while key Th1 chemokines (*CXCL9*, *CXCL10*, and *CXCL11*) were downregulated. Prototypical Th2 genes (*IL5*, *STAT6*, and *GATA3*) remained mostly unchanged, but modest increases in *IL4* and *IL13* were observed.

Unlike the other conjugates, **T1/2/N2** selectively provoked a Th17 immune response in PBMCs; it induced the transcription of *IL17A*, *IL17F*, and *IL22*, which collectively define the Th17 secretory phenotype, as well as *IL1B*, *IL23A* and *IL6*, which are essential for Th17 differentiation. In addition, *IL9R* and *OX40* (*TNFRSF4*), a costimulatory receptor implicated in IL-9-polarized response,^33^ were also selectively upregulated. Given that the IL-9/IL-9R axis contributes to Th17 differentiation,^34^ these data strongly suggest that **T1/2/N2** preferentially activates the Th17 T cell subset, important in host defense against microbial infections at mucosal surfaces by promoting local IgA responses.^35^ Our findings in PBMCs align with previous studies demonstrating that NOD2 stimulation, while inactive alone, acted synergistically with the TLR1/2 ligand Pam3CSK4 to enhance Th17 responses.^36^ Additionally, *MMP12*, *IL1B*, and *CXCL8* were significantly upregulated, whereas DC maturation markers *CD80* and *CD83* were only weakly increased, and *CD86* was downregulated. **T1/2/T7** exhibited a distinct immune profile, characterized by weak induction of canonical Th1 genes (*IL12A*, *IL12B*, *IFNG*, *CXCL10*, and *CXCL11*) and Th1-associated *IL15*. Moderate increases in *IL17A*, *IL17F*, and *IL22* further suggested Th17 activation, supported by the upregulation of *IL1B* and *IL6* (although *IL23A* was downregulated). **T1/2/T7** displayed a weak induction of Th2-related genes (*IL4*, *IL13*, and *CCL2*) and the Th2-promoting cytokine *IL10*. Similar to **T4/T7** and **T7/RI**, and likely due to the shared TLR7 agonist moiety, **T1/2/T7** significantly upregulated IFN-signaling-dependent genes (*IFIT1*, *IRF7*, *ISG15*, *OAS1*, and *RSAD2*), chemokine attractants (*CCL7*, *CCL8*, and *CCL2*), and DC maturation markers *CD80* and *CD83*.

### Conjugates **T4/T7** and **T7/RI** are Taken Up by PBMCs and Exhibit Receptor-Driven Activities

We next examined the cellular uptake of the two best-performing conjugates, **T4/T7** and **T7/RI**, in comparison with their corresponding unconjugated agonists as well as the stability of the conjugates toward intracellular enzymatic processing. To this end, extracellular media and cell lysates were collected following 18 h of stimulation of human PBMCs with **T4/T7**, **T7/RI**, or the individual agonists **T4**, **T7**, and **RI** (all at 1 μM), and analyzed by LC–MS (Table S4A). Quantitative analysis revealed that both conjugates were taken up by PBMCs, although to different extents. Specifically, extracellular and intracellular concentrations of **T4/T7** were 667.22 and 52.44 ng/mL, respectively, whereas the corresponding values for **T7/RI** were 209.44 and 126.21 ng/mL. In contrast, the unconjugated agonists showed negligible intracellular accumulation under the same conditions, consistent with limited cellular uptake. Notably, while the primary target of **T4** is extracellular, conjugation enabled partial internalization of the **T4**-containing construct, supporting the notion that a covalent linkage facilitates coordinated cellular delivery. In addition to intact conjugates, LC–MS analysis detected intracellular species arising from limited hydrolytic cleavage of amide bonds, including free agonists and linker-modified agonists (Tables S4B,C; Figures S3 and S4). These observations indicate that both conjugates undergo partial intracellular processing in the PBMCs. However, the extent of hydrolysis was low, and intact conjugates remained the predominant intracellular species. Importantly, a preliminary functional assessment of the anticipated degradation products revealed that, while these metabolites were capable of inducing cytokine production, their activity was consistently weaker than that of the parent conjugates (Figure S5). The markedly lower activity of degradation products combined with their substantially lower intracellular abundance thus suggests that intact conjugates are the primary functional entities driving the observed immune responses. This distinction is mechanistically relevant, as costimulation with unlinked mixtures of agonists can result in nonintegrative cellular responses, whereby individual cells respond preferentially to only one ligand rather than integrating signals from multiple receptors.^37^ In contrast, covalent linkage ensures copresentation of both agonistic moieties to the same immune cell, thereby promoting coordinated receptor engagement and more robust immune activation.

To further delineate the contribution of individual PRR ligands within **T4/T7** and **T7/RI** to PBMC activation, we conducted preliminary mechanistic studies using pharmacological inhibitors of the relevant signaling pathways. Specifically, we employed TAK242 (TLR4 antagonist),^38^ M5049 (TLR7 antagonist),^39^ and MRT67307 (RIG-I signaling inhibitor),^40^ either alone or in combination, at noncytotoxic concentrations (Figure S6). Inhibition of either TLR4 or TLR7 signaling significantly reduced cytokine production induced by **T4/T7**, whereas **T7/RI** activity was attenuated by the inhibition of either TLR7 or RIG-I signaling (Figure 5A; Table S5A). Combined inhibition of both pathways resulted in a further reduction of cytokine release for both conjugates, consistent with dual receptor engagement. Comparable trends were observed in a functional cytotoxicity assay, where cotreatment with the respective inhibitors completely abrogated **T4/T7**- and **T7/RI**-induced PBMC-mediated killing of K562 cancer cells (Figure 5B; Table S5B).

**Figure 5:**
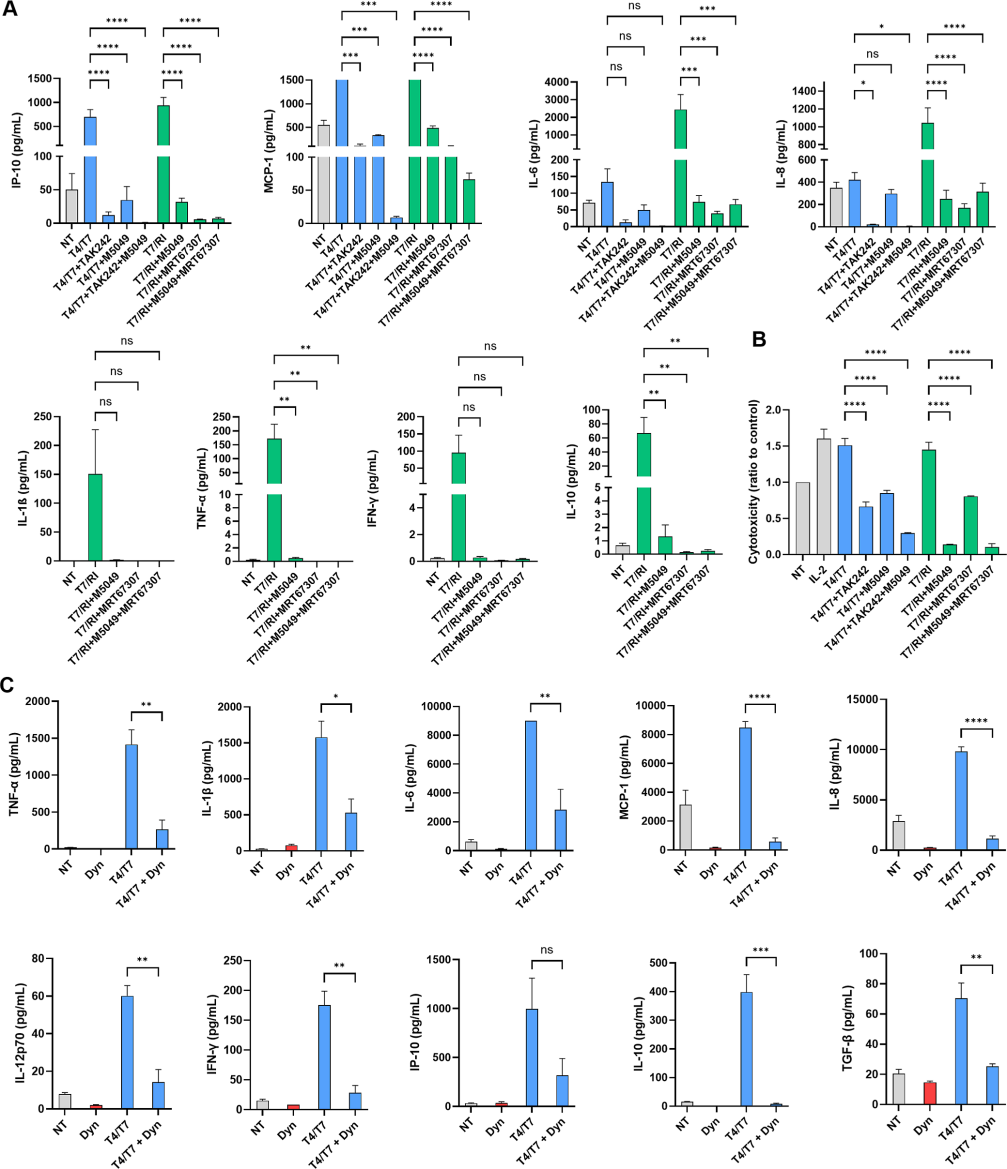
(A) Cytokine release from human PBMCs pretreated with signaling inhibitors (5 μM) and stimulated for 18 h with conjugates (100 nM) or vehicle (0.1% DMSO). Mean ± SEM of two independent experiments. One-way ANOVA with Bonferroni’s test compared **T4/T7** and **T7/RI** to their respective combinations with signaling inhibitors. *, *p* < 0.05; **, *p* < 0.01; ***, *p* < 0.001; ****, *p* < 0.0001. (B) Cytotoxicity of PBMCs against cancer cells after 18 h treatment as above, followed by 4 h coincubation with K562 cells. Data shown relative to vehicle control (NT, 0.1% DMSO); mean ± SEM of two independent experiments. One-way ANOVA with Bonferroni’s test compared **T4/T7** and **T7/RI** to their respective combinations with signaling inhibitors. *, *p* < 0.05; **, *p* < 0.01; ***, *p* < 0.001; ****, *p* < 0.0001. (C) Cytokine release from human PBMCs stimulated for 18 h with **T4/T7** (1 μM) or vehicle (0.1% DMSO) in the absence or presence of Dynasore (80 μM). Mean ± SEM of two independent experiments. Unpaired *t* test compared **T4/T7** to its combination with Dynasore; *, *p* < 0.05; **, *p* < 0.01; ***, *p* < 0.001; ****, *p* < 0.0001.

Although **T4/T7** exhibited only weak direct TLR4 agonism in reporter assays, we hypothesized that the TLR4-targeting moiety may facilitate receptor-mediated endocytosis, thereby enhancing the intracellular delivery of the TLR7 agonistic component to endosomal compartments. Indeed, structurally related weak TLR4 ligands have been shown to induce TLR4 internalization in a dose-dependent manner.^41^ Consistent with this model, pharmacological inhibition of endocytosis using Dynasore significantly reduced cytokine induction by **T4/T7** (Figure 5C), supporting a role for receptor-mediated uptake in the immunostimulatory activity of this conjugate.

### Conjugated PRR Ligands Demonstrate Ex Vivo Adjuvant Activity

After showing the efficacy of conjugates in eliciting nonspecific immune responses, we focused on their ability to enhance antigen-specific immunity. To assess how dual PRR stimulation influences antigen presentation by DCs and the generated T cell responses, we evaluated the conjugates in an ex vivo coculture system with murine BMDCs and naïve ovalbumin (OVA)-specific CD4^+^ and CD8^+^ T cells, isolated from OT-II and OT-I mice, respectively. BMDCs were treated with conjugates and soluble OVA protein, washed, and then cocultured for 3 days with carboxyfluorescein succinimidyl ester (CFSE)-labeled T cells. To quantify the effects, we used the proliferation index as a proxy for T cell response, which corresponds to the average number of divisions per responding T cell in the coculture. Preactivation of BMDCs with conjugated PRR ligands significantly enhanced their ability to induce antigen-specific activation and proliferation of both CD4^+^ and CD8^+^ T cells, as measured by the upregulation of the early activation marker CD25 and CFSE dilution (Figure 6A,B, Table S6A). While all conjugates activated CD8^+^ T cells similarly and outperformed stimulation with LPS, conjugates **T1/2/T7** and **T1/2/N2** slightly surpassed **T4/T7** and **T7/RI** in their ability to activate CD4^+^ T cells.

**Figure 6:**
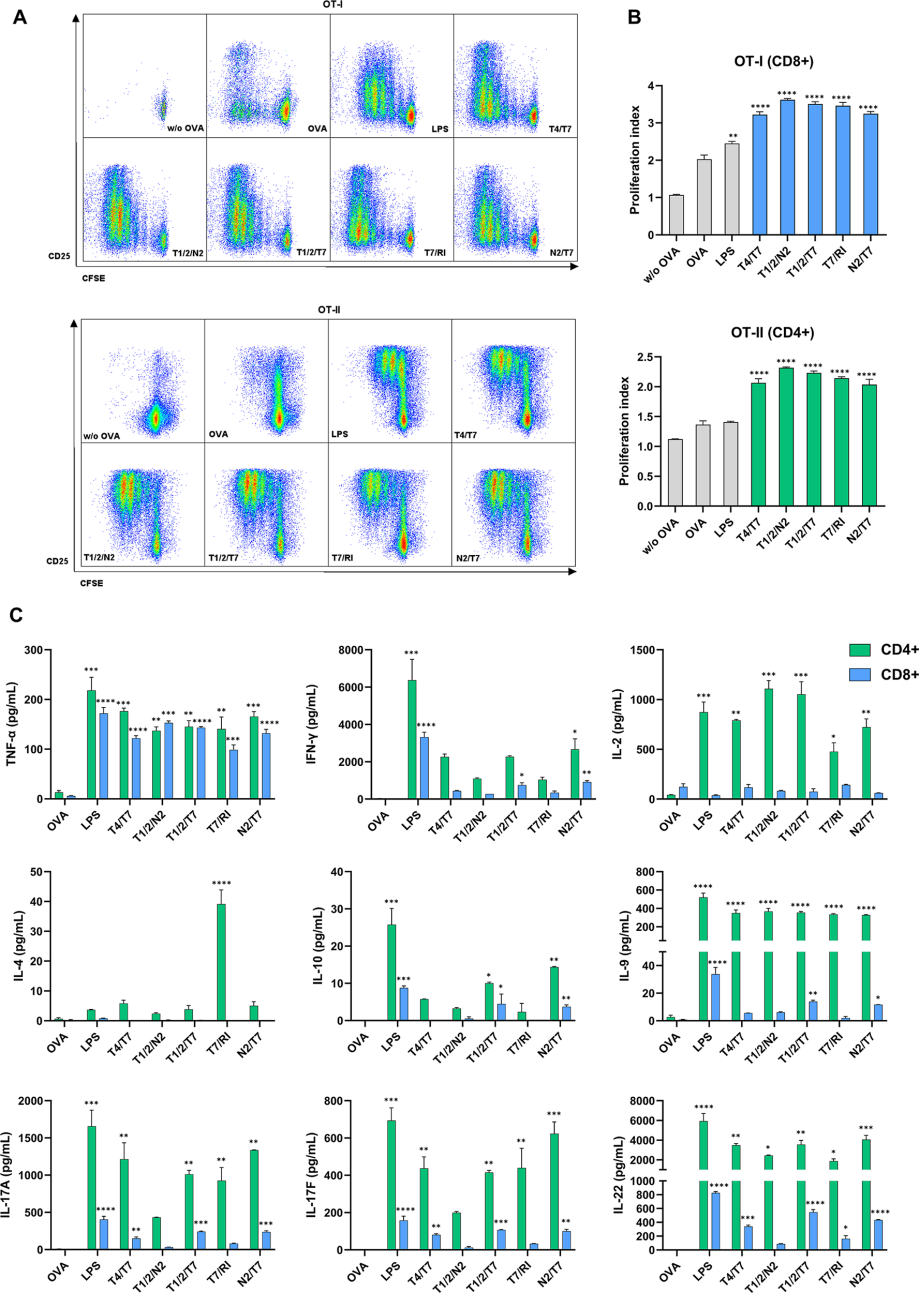
Treatment of BDMCs with conjugates promotes their antigen-presenting activity and enhances antigen-specific activation and proliferation of CD4^+^ and CD8^+^ T cells. BMDCs from C57BL/6 mice were treated for 18 h with conjugates (1 μM), LPS (1 μg/mL), or vehicle (0.1% DMSO) in the presence of OVA (50 μg/mL). CFSE-labeled OVA-specific CD4^+^ or CD8^+^ T cells (isolated from OT-II or OT-I mouse splenocytes, respectively) were added to the treated and washed BMDCs and cocultured for 72 h. (A) Representative dot plots show CD25 expression and CFSE dilution in live Thy1.2^+^/CD4^+^ (OT-II) and Thy1.2^+^/CD8^+^ (OT-I) T cells. (B) Pooled data from (A), expressed as proliferation indexes. (C) Cytokine concentrations in BMDC/T cell coculture supernatants following 72 h coincubation. Data are mean ± SEM of duplicates of two independent experiments. *, *p* < 0.05, **, *p* < 0.01, ***, *p* < 0.001, ****, *p* < 0.0001 versus vehicle-treated control. Statistical significance was determined using one-way ANOVA with posthoc Dunnett’s test comparing conjugates versus OVA.

Subsequent analysis of the cytokine profiles in the supernatants of BMDC/T cell cocultures (Figures 6C, S7, Table S6B) revealed notable differences between the conjugates, particularly in terms of cytokine production by CD8^+^ T cells. While all conjugates induced the secretion of TNF-α, stimulation with **T1/2/T7** resulted in significantly elevated levels of Th2 cytokines (IL-9, IL-10, IL-13) and Th17 cytokines (IL-17A, IL-17F, IL-22), along with a modest increase in Th1-associated IFN-γ. A similar, though less pronounced, Th2/Th17-polarized profile was observed with **T4/T7**. In contrast, the cytokine profiles elicited from CD4^+^ T cells were more similar across conjugates. Stimulation with all conjugates increased TNF-α and IL-2 secretion, with the highest levels of IL-2 observed for conjugates **T1/2/T7** and **T1/2/N2**, followed by **T4/T7** and **T7/RI**. Conjugates **T1/2/T7**, **T4/T7,** and **T7/RI** also significantly elevated the levels of Th2 cytokines (IL-9, IL-10, and IL-13) and Th17 cytokines (IL-17A, IL-17F, and IL-22), with **T7/RI** being the only conjugate that elicited detectable levels of IL-4.

The effects of conjugate **T1/2/N2** on Th2- and Th17-associated cytokines were less pronounced, which contrasts with the results obtained in PBMCs, where **T1/2/N2** induced the most notable Th17-biased response. Overall, the results from this murine model were not entirely consistent with those observed in human PBMCs. Unlike the Th1-biased cytokine profiles induced by **T4/T7** and **T7/RI** in human PBMCs—supported by transcriptomic analysis—murine BMDCs stimulated with these conjugates elicited prominent levels of Th1 cytokines but also induced some Th2/Th17 cytokines. These differences are likely due to the fact that murine BMDCs respond poorly to TLR7 ligands,^42^ as well as the absence of accessory cell effects observed in PBMCs.

### Conjugated PRR Ligands Exhibit In Vivo Adjuvant Activity

Encouraged by the promising in vitro and ex vivo results, we next evaluated the adjuvant potential of conjugates in a murine vaccination model by measuring the B cell responses. Mice were vaccinated using a prime-boost regimen with an OVA as the model antigen and conjugates **T4/T7**, **T7/RI**, **T1/2/T7**, and **T1/2/N2** as adjuvants. All conjugates were administered at doses equimolar to 50 μg of the previously reported NOD2/TLR7 conjugate **N2/T7** to enable direct comparison across studies. Alum (100 μg) was included as a universal benchmark. OVA-specific IgG, IgG1, and IgG2a responses were measured to delineate B cell responses (Figure 7A, Table S7A). **T4/T7** and **T7/RI** induced robust systemic immune responses, with IgG titers several orders of magnitude higher than those elicited by OVA alone or with alum. While **T1/2/N2** and **T1/2/T7** had a lesser effect on total IgG titers, their impact remained notable. Importantly, immunization with **T4/T7** and **T7/RI** increased both IgG1 and IgG2a titers, indicating the induction of Th2 and Th1 responses, respectively, consistent with previous reports. Specifically, combinations of TLR4 and TLR7 agonists have been shown to elicit balanced Th1/Th2 responses to influenza antigens,^43,44^ while nanoparticle-based codelivery of TLR7 and RIG-I agonists generated strong cellular responses in an influenza model.^45^ In contrast, conjugates featuring the TLR1/2 agonist (**T1/2/N2** and **T1/2/T7**) favored IgG1 over IgG2a, suggesting a Th2-skewed profile. This aligns with prior findings that TLR2 activation promotes antigen-specific responses biased toward Th2. However, our results diverge from studies in which coengagement of TLR2 with Th1-polarizing TLRs or NOD2 enhanced Th1-type responses. For example, coadministration of HIV-1 p24 with the dual TLR2/6/TLR7 ligand CL413 elevated anti-p24 IgG titers, predominantly IgG1, but also IgG2a.^7^ Similarly, the dual TLR2/6/NOD2 ligand CL429 induced a balanced Th1/Th2 profile and enhanced mucosal immunity, as indicated by elevated IgA.^8^ Notably, both studies used the p24 antigen, nanoparticles, and TLR2/6 agonists. These factors, individually or in combination, may have contributed to differing outcomes. As expected, alum induced a Th2-skewed response, consistent with historical data. Interestingly, the in vivo adjuvant activity data did not align with the findings from the BMDC/T cell assay but correlated well with the RNA sequencing data from PBMCs (expression of CD80, CD83, CD86, and HLA-DR), thus attesting to the importance of accessory cells. Collectively, these results underscore the potential of **T4/T7** and **T7/RI** as vaccine adjuvants capable of eliciting potent, yet balanced, cellular and humoral immune responses.

**Figure 7:**
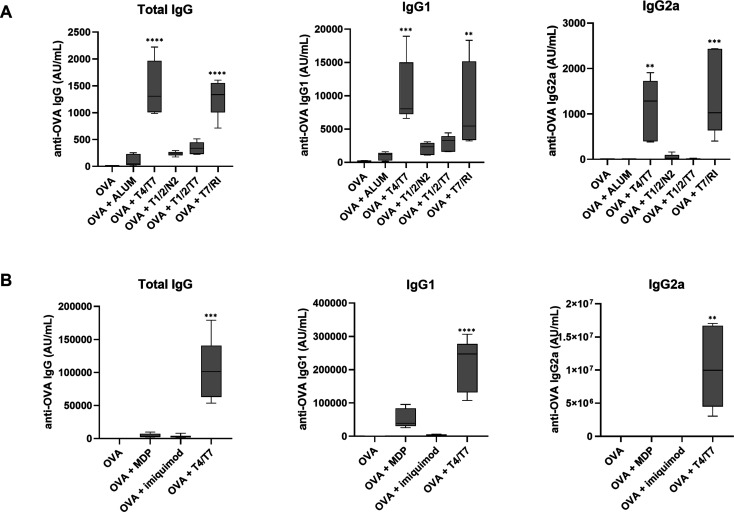
(A) In vivo adjuvant activity of conjugates. NIH/OlaHsd mice (*n* = 6/group) were immunized s.c. on days 0, 21, and 42 with OVA (10 μg) alone or with conjugates (equimolar to 50 μg **N2/T7**) or Al-hydrogel (100 μg). OVA-specific IgG, IgG1, and IgG2a responses were measured 7 days after the final dose; **, *p* < 0.01, ***p < 0.001, ****p < 0.0001 vs control (one-way ANOVA, Dunnett’s test). (B) Benchmarking of **T4/T7** adjuvant activity against established adjuvants. NIH/OlaHsd mice (*n* = 6/group) were immunized s.c. on days 0, 21, and 42 with OVA (10 μg) in the presence or absence of **T4/T7** (42 μg), imiquimod (72 μg), or muramyl dipeptide (148 μg). OVA-specific IgG, IgG1, and IgG2a responses were measured 7 days after the final dose; **, *p* < 0.01, ***p < 0.001, ****p < 0.0001 vs control (one-way ANOVA, Dunnett’s test).

To further contextualize the adjuvant activity of **T4/T7**, we benchmarked it against established PRR agonists, imiquimod, and MDP, commonly used as reference adjuvants. Under identical immunization conditions, **T4/T7** elicited stronger and more balanced humoral responses than those of these benchmarks (Figure 7B, Table S7B), highlighting the potential of covalent PRR agonist conjugation in adjuvant design.

### Conjugate **T4/T7** Exhibits Antitumor Activity In Vivo

Consistent with the strong Th1-skewed immunostimulatory activity observed in cytokine assays, both **T4/T7** and **T7/RI** upregulated granzyme B and perforin expression and amplified PBMC cytolytic activity against cancer cells. We therefore further evaluated their antitumor potential in the B16F10 melanoma model, benchmarking their efficacy against resiquimod, a potent TLR7/8 agonist^46^ with established antitumor activity in various murine tumor models.^47,48^ Mice were injected with B16F10 tumor cells and randomized into four treatment groups: vehicle, resiquimod (20 μg/mouse, ∼1 mg/kg), **T4/T7**, and **T7/RI** (both administered at doses equimolar to resiquimod). Intratumoral injections were administered every two or 3 days for a total of six doses over 2 weeks. Tumor growth was monitored throughout treatment and follow-up (Figure 8A, Table S8A). Treatment with **T4/T7** and resiquimod led to significant tumor growth suppression compared to vehicle, as determined by ANOVA mixed-effects modeling (*p* = 0.0033 and *p* = 0.0014, respectively; Figure 8B). Both compounds inhibited tumor growth during the treatment phase and delayed progression after treatment cessation. On day 18, **T4/T7** achieved a tumor growth inhibition (TGI) of 59%, compared to 56% for resiquimod, while **T7/RI** showed a modest TGI of 31%. Tumor/control (T/C) ratios also confirmed the superior efficacy of **T4/T7** over resiquimod (48 vs 53% on day 18; Table S8B). In addition, both **T4/T7** and resiquimod significantly prolonged survival relative to the vehicle (*p* = 0.0019 and *p* = 0.0004, respectively; Figure 8C, Table S8C). These findings support earlier reports that intratumoral delivery of TLR4 and TLR7/8 agonists can synergize with checkpoint inhibitors to eradicate tumors.^49^ In contrast, despite robust cytolytic effects observed in vitro, **T7/RI** failed to translate into comparable antitumor efficacy in vivo, unlike CV8102, a nonchimeric TLR7/RIG-I dual agonist with clinical efficacy in hepatocellular carcinoma.^50^

**Figure 8:**
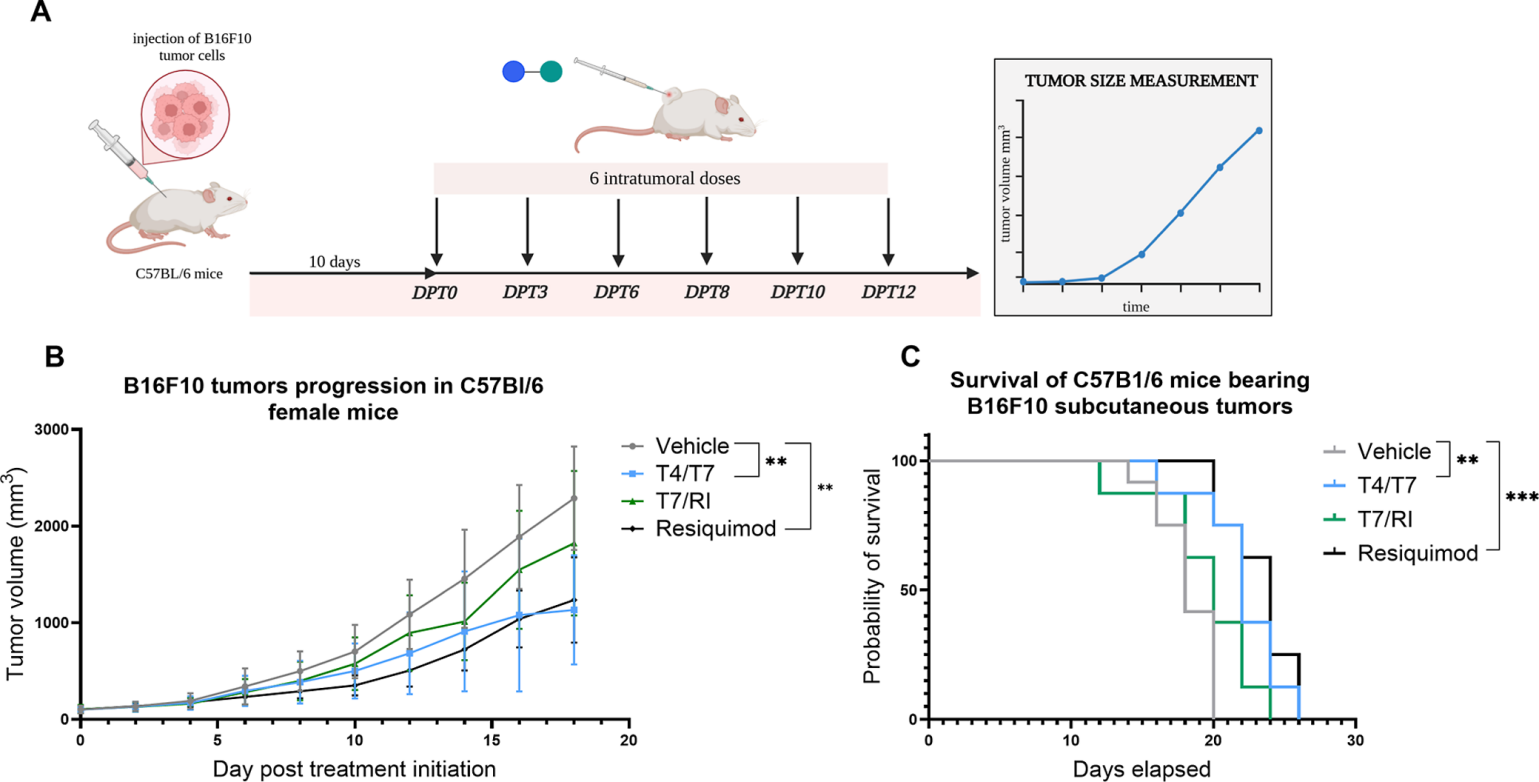
(A) Tumor challenge experiment design. C57BL/6 mice (*n* = 8/group) were injected with 5 × 10^5^ B16F10 tumor cells. After tumors reached ∼80 mm^3^ (day 10), mice were randomized and treated intratumorally with vehicle, **T4/T7**, **T7/RI**, or resiquimod every 2–3 days for six doses. (B) Tumor growth. Volume was recorded every other day until tumors exceeded 3000 mm^3^. Data (DPT0–DPT18) are mean ± SEM. Tumor growth was analyzed using two-way ANOVA with a linear mixed-effects model; *p < 0.05, **p < 0.01. (C) Survival analysis. Kaplan–Meier survival curves (DPT0–DPT26) were analyzed using the Mantel–Cox log-rank test. **, *p* < 0.01; ***, *p* < 0.001 vs control.

Preliminary safety assessment revealed no significant changes in body weight across treatment groups (Figure S8). Animals were additionally monitored for overt clinical signs throughout the study, and no treatment-related adverse effects were observed (Table S9). Given the absence of analyses of tumor-infiltrating immune cell populations, we intentionally refrain from attributing the observed antitumor effects to specific immune mechanisms. Accordingly, these in vivo data are best interpreted as functional proof-of-concept that immune-active conjugated PRR ligands can translate into measurable antitumor efficacy following intratumoral administration. Taken together, our results demonstrate that **T4/T7** induces robust antitumor responses, even in the poorly immunogenic and aggressive B16F10 melanoma model. Given the higher baseline immune infiltration characteristic of more immunogenic tumors, intratumoral treatment with **T4/T7** may yield an even greater therapeutic benefit in such settings.

## Conclusions

In this study, we sought to harness synergistic innate immune cross-talk by integrating distinct PRR ligands into chemically defined chimeric conjugates. Using a two-step phenotypic screening strategy in primary human PBMCs, we identified several conjugated PRR ligand combinations that elicited cytokine profiles and functional immune activities distinct from those induced by unlinked agonist mixtures. These findings highlight the importance of the covalent linkage for enforcing coordinated receptor engagement and enabling higher-order immune responses, even when the targeted PRRs reside in different cellular compartments. Among the evaluated constructs, the TLR1/2-NOD2 conjugate displayed a unique capacity to promote Th17-associated immune responses, whereas conjugated TLR4/TLR7 and TLR7/RIG-I ligands induced broad innate immune activation in vitro and enhanced antigen-specific humoral responses in vivo. Notably, intratumoral administration of the conjugated TLR4/TLR7 ligand resulted in significant TGI and prolonged survival in the aggressive and poorly immunogenic B16F10 melanoma model, providing a functional proof of concept for the antitumor potential of covalently linked PRR agonists.

Collectively, these findings establish covalent PRR agonist conjugation as a versatile and modular chemical approach for rationally shaping innate immune response through defined agonist pairing. By directly comparing covalently linked constructs with their unconjugated counterparts across complementary models, this study provides insight into how enforced codelivery and spatial organization of PRR ligands can modulate immune signaling profiles. Future studies exploring linker architecture and spatial parameters may further refine these responses and enable the additional tuning of immunological activity. Although further mechanistic refinement, safety evaluation, and translational investigation will be necessary to advance these molecules toward clinical application, the conjugates described herein represent both functional lead structures and a conceptual foundation for the development of next-generation immunomodulatory agents with potential utility in vaccine adjuvant design and immunotherapeutic strategies.

## Experimental Section

### Materials

Chemicals were obtained from Sigma-Aldrich (St. Louis, MO, U.S.A.), Tokyo Chemical Industry (Tokyo, Japan), Acros Organics (Geel, Belgium), Enamine (Monmouth Junction, NJ, U.S.A.), and Apollo (Stockport, U.K.) and were used without further purification. LPS (from *E. coli*, no. O55:B5), TAK242, M5049, and MRT67307 were obtained from InvivoGen (San Diego, CA, U.S.A.). Dynasore was purchased from BLD Pharmatech (Reinbek, Germany). MDP, Imiquimod, Pam3CSK4, and Poly­(I/C) (HMW) (LyoVec) were purchased from InvivoGen (San Diego, CA, U.S.A.). Compound **N2/T7** (Ethyl *N* ^*5*^-(2-(2-(4-((6-amino-2-butoxy-8-hydroxy-9*H*-purin-9-yl)­methyl)­benzamido)­ethoxy)­ethyl)-*N* ^*2*^-((*E*)-3-(4-hydroxy-3-methoxyphenyl)­acryloyl)­glycyl-l-valyl-d-glutaminate) was prepared as previously described.^9^ Compounds **N2**,^17^ **T1/2**,^20^ **T4**,^21^ **T7,** ^23^ and **RI** ^24^ were prepared according to the reported procedures. Analytical TLC was performed on Merck 60 F254 silica gel plates (0.25 mm) with visualization using ultraviolet light, ninhydrin, and potassium permanganate. Flash column chromatography was carried out on Merck silica gel 60 (particle size 240–400 mesh) and on a Biotage Isolera One flash chromatograph using Biotage Sfär C18 D Duo 100 Å 30 μm 30 g. 1H and 13C NMR spectra were recorded at 400 and 100 MHz, respectively, on an Avance III spectrometer (Bruker Corporation, Billerica, MA, U.S.A.) in CDCl_3_ or DMSO-*d*_6_, with tetramethylsilane as the internal standard. Mass spectra were obtained using an Exactive Plus orbitrap mass spectrometer (Thermo Fisher Scientific, Waltham, MA, U.S.A.) or on an Expression CMS mass spectrometer (Advion Inc., Ithaca, NY, U.S.A.). Analytical UHPLC was performed on a Dionex UltiMate 3000 Rapid Separation Binary System (Thermo Fisher Scientific, Waltham, MA, U.S.A.) equipped with an autosampler, a binary pump system, a photodiode array detector, a thermostated column compartment, and the Chromeleon Chromatography data system. The columns used were a Waters Acquity UPLC BEH C18 (1.7 μm, 2.1 mm × 50 mm) or a Waters Acquity UPLC CSH C18 (1.7 μm, 2.1 mm × 50 mm) with a flow rate of 0.3 mL/min. The eluent was a mixture of 0.1% TFA in water (A) and MeCN (B) with the gradient (%B) as follows: 0–10 min, 5–95%; 10–12 min, 95%; 12–12.5 min, 95–5%. For compound T7/RI, the mobile phase consisted of 0.1% TFA in water (A) and MeCN (B), employing the following gradient: 95% A to 5% A in 7 min, then 95% B for 1 min, with a flow rate of 0.3 mL/min. The columns were thermostated at 40 °C. All the compounds tested were established to be ≥95% pure. The analytical data here were identical with those reported previously. The assembly of the final compounds was performed as described below.

### Synthesis and Characterization of Compounds

#### General Procedures

##### General Procedure A: Boc Deprotection

The *tert*-butyloxycarbonyl (Boc)-protected compound was added to an ice-chilled mixture of trifluoroacetic acid (TFA) and dichloromethane (DCM) (1:5), and the mixture was allowed to warm to room temperature. After 3 h, the solvent was evaporated in vacuo. The residue was washed three times with diethyl ether.

##### General Procedure B: COMU-Mediated Coupling

To an ice-chilled solution of the amine or alcohol (1–1.2 equiv) and carboxylic acid (1–1.2 equiv) in anhydrous dimethylformamide (DMF), *N*,*N*-diisopropylethylamine (DIPEA; 4 equiv) and 1-[(1-(cyano-2-ethoxy-2-oxoethylideneaminooxy)-dimethylamino-morpholinomethylene)]-methanaminium hexafluorophosphate (COMU; 1.2 equiv) were added, and the mixture was allowed to warm to room temperature. The stirring was continued overnight, after which the mixture was washed twice with saturated NaHCO_3_ and once with brine. The organic layer was dried in anhydrous Na_2_SO_4_ and concentrated in vacuo.

##### General Procedure C: HATU-Mediated Coupling

To an ice-chilled solution of the amine or alcohol (1–1.5 equiv) and carboxylic acid (1–1.5 equiv) in anhydrous dimethylformamide (DMF), *N*,*N*-diisopropylethylamine (DIPEA; 4 equiv) and 1-[bis­(dimethylamino)­methylene]-1H-1,2,3-triazolo­[4,5-*b*]­pyridinium 3-oxide hexafluorophosphate (HATU; 1.5 equiv) were added, and the mixture was allowed to warm to room temperature. The stirring was continued overnight, after which the mixture was washed twice with 1 M HCl and saturated NaHCO_3_ and once with brine. The organic layer was dried over anhydrous Na_2_SO_4_ and concentrated in vacuo.

#### Synthesis of Compounds

##### *tert*-Butyl (2-(2-(4-((6-Amino-2-butoxy-8-hydroxy-9*H*-purin-9-yl)­methyl)­benzamido)­ethoxy)­ethyl)­carbamate (**1**)

*tert*-Butyl (2-(2-aminoethoxy)­ethyl)­carbamate (380 mg, 1.86 mmol) and DIPEA (0.523 mL, 2.99 mmol) were dissolved in DCM (0.5 mL) and added to the stirring solution of compound **T7** (200 mg, 0.56 mmol) in DMSO (3 mL). To an ice-chilled solution was added COMU (0.640 g, 1.49 mmol), and the mixture was stirred at room temperature for 3 h. Subsequently, ethyl acetate (40 mL) was added, and the mixture was cooled in ice for 1 h, after which 1 M NaHCO_3_ (40 mL) was added. The precipitate obtained, after concentrating the mixture in vacuo, was filtered and washed with water and diethyl ether to give compound **1** as an off-white solid. Yield (160 mg, 53%). ^1^H NMR (400 MHz, DMSO-*d*_6_) δ: 9.98 (s, 1H), 8.44 (t, *J* = 6.4, 1H), 7.78 (d, *J* = 8.2, 2H), 7.34 (d, *J* = 8.2, 2H), 6.76 (t, *J* = 6.8, 1H), 6.47 (s, 2H), 4.90 (s, 2H), 4.12 (t, *J* = 7.0, 2H), 3.53–3.45 (m, 2H), 3.43–3.36 (m, 4H), 3.14–2.97 (m, 2H), 1.69–1.52 (m, 2H), 1.42–1.27 (m, 11H), 0.89 (t, *J* = 7.4, 3H).

##### 4-((6-Amino-2-butoxy-8-hydroxy-9*H*-purin-9-yl)­methyl)-*N*-(2-(2-(2-((4-oxo-3-phenyl-4,5-dihydro-3H-pyrimido­[5,4-*b*]­indol-2-yl)­thio)­acetamido)­ethoxy)­ethyl)­benzamide (**T4/T7**)

Compound **1** (89 mg, 0.164 mmol, 1.1 equiv) was deprotected using General procedure A and coupled to **T4** (52 mg, 0.149 mmol, 1 equiv) using General procedure B. The crude product was purified by Isolera One flash chromatography (acetonitrile/0.1% TFA 20% → 100%) to give compound **T4/T7** as a pale-yellow solid (38 mg, 10%). ^1^H NMR (400 MHz, DMSO-*d*_6_) δ: 12.10 (s, 1H), 9.98 (s, 1H), 8.44 (t, *J* = 5.5 Hz, 1H), 8.32 (t, *J* = 5.5 Hz, 1H), 8.06–7.99 (m, 1H), 7.82–7.74 (m, 2H), 7.64–7.55 (m, 3H), 7.53–7.42 (m, 4H), 7.39–7.30 (m, 2H), 7.27–7.19 (m, 1H), 6.47 (s, 2H), 4.88 (s, 2H), 4.12 (t, *J* = 6.6 Hz, 2H), 3.91 (s, 2H), 3.54–3.34 (m, 5H), 3.31–3.18 (m, 2H), 1.67–1.51 (m, 2H), 1.43–1.31 (m, 2H), 0.88 (t, *J* = 7.4 Hz, 3H). ^13^C NMR (100 MHz, DMSO) δ: 165.80, 164.73, 158.79, 153.63, 151.07, 150.89, 147.82, 146.49, 138.88, 137.60, 135.92, 134.74, 132.22, 128.55, 128.24, 126.11, 125.98, 125.88, 119.07, 119.00, 118.84, 117.94, 111.50, 96.95, 67.45, 67.42, 64.52, 40.78, 35.14, 29.25, 17.41, 12.39. HRMS *m*/*z* calculated for C_39_H_41_O_6_N_10_S, 777.2926 (M + H)^+^; found, 777.2903.

##### *tert*-Butyl (2-(2-(2-(4-((6-amino-2-butoxy-8-hydroxy-9H-purin-9-yl)­methyl)­benzamido)­ethoxy)­ethoxy)­ethyl)­carbamate (**2**)

Compound **10** (1.157 g, 4.659 mmol, 3.33 equiv) and DIPEA (1.299 mL, 7.457 mmol, 5.33 equiv) were dissolved in DCM (1 mL) and added to the stirring solution of compound **T7** (500 mg, 1.399 mmol, 1 equiv) in DMSO (20 mL). After cooling the reaction mixture in ice, COMU (1.594 g, 3.722 mmol, 2.66 equiv) was added, and the mixture was stirred at rt for 20 h. Ethyl acetate (100 mL) and a 1 M NaHCO_3_ solution (75 mL) were added. After the mixture was concentrated in vacuo, the mixture was cooled in ice for 1 h. The precipitate was filtered and washed with water and diethyl ether to afford subject compound **2**. Yield (450 mg, 55%). ^1^H NMR (400 MHz, DMSO-*d*_6_) δ: 10.14 (s, 1H), 8.48 (t, *J* = 5.6 Hz, 1H), 7.84–7.76 (m, 2H), 7.39–7.30 (m, 2H), 6.84–6.68 (m, 1H), 6.52 (s, 2H), 4.90 (s, 2H), 4.12 (t, *J* = 6.6 Hz, 2H), 3.61–3.45 (m, 8H), 3.45–3.38 (m, 2H), 3.07–3.02 (m, 2H), 1.69–1.53 (m, 2H), 1.37–1.29 (m, 11H), 0.89 (t, *J* = 7.4 Hz, 3H).

##### 2-(2-Naphthamido)-*N*-(2-(2-(2-(4-((6-amino-2-butoxy-8-hydroxy-9*H*-purin-9-yl)­methyl)­benzamido)­ethoxy)­ethoxy)­ethyl)­benzo­[*d*]­thiazole-6-carboxamide (**T7/RI**)

Compound **2** (120 mg, 0.205 mmol, 1 equiv) was deprotected using general procedure A and coupled to **RI** (79 mg, 0.226 mmol, 1.1 equiv) using general procedure B. The crude product was purified by flash chromatography (dichloromethane/methanol 25:1) to give compound **T7/RI** as a white solid (18 mg, 11%). ^1^H NMR (400 MHz, DMSO-*d*_6_) δ: 13.27 (s, 1H), 10.05 (s, 1H), 8.93 (s, 1H), 8.70–8.64 (m, 1H), 8.64–8.59 (m, 1H), 8.59–8.53 (m, 1H), 8.26–8.21 (m, 1H), 8.20–8.15 (m, 2H), 8.14–8.09 (m, 1H), 8.05–8.01 (m, 1H), 7.97–7.89 (m, 1H), 7.88–7.82 (m, 2H), 7.79–7.71 (m, 2H), 7.41 (d, *J* = 8.3 Hz, 2H), 6.53 (s, 2H), 4.96 (s, 2H), 4.18 (t, *J* = 6.6 Hz, 2H), 3.62 (s, 12H), 1.76–1.59 (m, 2H), 1.42 (q, *J* = 7.4 Hz, 2H), 0.95 (t, *J* = 7.4 Hz, 3H). ^13^C NMR (100 MHz, DMSO) δ: 166.41, 166.39, 152.40, 149.26, 140.32, 135.34, 134.10, 132.44, 131.84, 130.37, 130.11, 129.80, 129.09, 128.81, 128.21, 127.91, 127.78, 127.58, 126.01, 124.79, 121.73, 120.26, 98.68, 70.07, 69.43, 69.39, 67.33, 62.48, 42.77, 30.80, 25.96, 19.08, 14.10. HRMS *m*/*z* calculated for C_42_H_44_O_7_N_9_S, 818.3079 (M + H)^+^; found, 818.3069.

##### Ethyl 6-(2-((4-Oxo-3-phenyl-4,5-dihydro-3*H*-pyrimido­[5,4-*b*]­indol-2-yl)­thio)­acetamido)­hexanoate (**3**)

Compound **3** was synthesized from **11** (167 mg, 0.854 mmol, 1.5 equiv) and **T4** (200 mg, 0.569 mmol, 1 equiv) using general procedure C as an off-white solid. Yield (150 mg, 53%). ^1^H NMR (400 MHz, DMSO-*d*_6_) δ: 12.11 (s, 1H), 8.26–8.17 (m, 1H), 8.08–7.99 (m, 1H), 7.63–7.57 (m, 3H), 7.53–7.42 (m, 4H), 7.28–7.20 (m, 1H), 4.01 (q, *J* = 7.1 Hz, 2H), 3.88 (s, 2H), 3.05 (q, *J* = 6.6 Hz, 2H), 2.11 (t, *J* = 6.9, 6.5 Hz, 2H), 1.44–1.36 (m, 3H), 1.28–1.20 (m, 5H), 1.15 (t, *J* = 7.1 Hz, 3H).

##### 6-(2-((4-Oxo-3-phenyl-4,5-dihydro-3*H*-pyrimido­[5,4-*b*]­indol-2-yl)­thio)­acetamido)­hexanoic Acid (**4**)

To a stirring solution of compound **3** (150 mg, 0.303 mmol) in methanol (5 mL) was added 1 M NaOH (3 mL). The mixture was stirred at room temperature overnight. The next day, H_2_O (20 mL) was added, and methanol was evaporated in vacuo. The water phase was washed with ethyl acetate (20 mL) and acidified with 1 M HCl to pH 3. The product was extracted two times with ethyl acetate (20 mL). The combined organic phases were washed with brine (10 mL), dried over anhydrous Na_2_SO_4_, and concentrated in vacuo to give compound **4** as an off-white solid. Yield (141 mg, 100%). ^1^H NMR (400 MHz, DMSO-*d*_6_) δ: 12.10 (s, 1H), 11.97 (s, 1H), 8.22 (t, *J* = 5.6 Hz, 1H), 8.11–8.00 (m, 1H), 7.62–7.59 (m, 3H), 7.54–7.39 (m, 4H), 7.33–7.18 (m, 1H), 3.88 (s, 2H), 3.05 (q, *J* = 6.6 Hz, 2H), 2.08 (t, *J* = 7.4 Hz, 2H), 1.42–1.37 (m, 2H), 1.25–1.22 (m, 4H).

##### Dicyclopentyl ((*E*)-3-(3-Methoxy-4-((6-(2-((4-oxo-3-phenyl-4,5-dihydro-3*H*-pyrimido­[5,4-*b*]­indol-2-yl)­thio)­acetamido)­hexanoyl)­oxy)­phenyl)­acryloyl)­glycyl-l-valyl-d-glutamate (**T4/N2**)

This was synthesized from **4** (141 mg, 0.303 mmol, 1.2 equiv) and **N2** (156 mg, 0.253 mmol, 1 equiv) using general procedure B. The crude product was purified by flash ^1^H NMR (400 MHz, Chloroform-*d*) δ: 10.65 (s, 1H), 8.05 (d, *J* = 8.0 Hz, 1H), 7.60–7.44 (m, 8H), 7.40–7.31 (m, 4H), 7.21–7.16 (m, 1H), 7.01–6.97 (m, 2H), 6.87 (d, *J* = 8.1 Hz, 1H), 6.39 (d, *J* = 15.6 Hz, 1H), 5.16–5.10 (m, 2H), 4.53–4.42 (m, 2H), 4.13–4.05 (m, 2H), 3.88–3.83 (m, 2H), 3.72 (s, 2H), 3.29 (q, *J* = 6.5 Hz, 2H), 2.34 (t, *J* = 7.4 Hz, 4H), 2.15–2.11 (m, 1H), 1.97 (s, 3H), 1.85–1.75 (m, 5H), 1.72–1.51 (m, 19H), 1.34 (t, 2H), 0.94–0.88 (m, 6H). ^13^C NMR (100 MHz, CDCl_3_) δ: 172.60, 171.58, 171.38, 171.28, 169.63, 168.76, 166.51, 155.85, 153.30, 151.14, 140.86, 140.72, 139.47, 138.20, 135.48, 133.61, 130.39, 129.90, 129.88, 129.15, 129.13, 128.19, 123.00, 121.01, 120.59, 120.47, 120.35, 120.22, 119.34, 113.11, 111.64, 78.67, 77.51, 77.24, 58.62, 55.81, 51.99, 43.48, 39.54, 36.07, 33.51, 32.68, 32.63, 32.57, 32.49, 30.96, 30.72, 30.64, 28.93, 26.86, 26.10, 24.32, 23.68, 23.63, 19.32, 17.89. HRMS *m*/*z* calculated for C_56_H_68_O_12_N_7_S, 1062.4641 (M + H)^+^; found, 1062.4635.

##### 2-(1-(2-(Methylamino)-5-nitrophenyl)-1*H*-imidazole-4-yl)-5-(trifluoromethyl)­phenyl 6-((*tert*-Butoxycarbonyl)­amino)­hexanoate (**5**)

This was synthesized from **T1/2** (50 mg, 0.134 mmol, 1 equiv) and **12** (37 mg, 0.161 mmol, 1.2 equiv) using general procedure C (1 M HCl was not used for extraction). The crude product was purified by flash chromatography (hexane/ethyl acetate 1:1) to give compound **5** as a white solid (35 mg, 44%). ^1^H NMR (400 MHz) δ: 8.32 (d, *J* = 8.2 Hz, 1H), 8.22 (dd, *J* = 9.3, 2.7 Hz, 1H), 8.04–7.97 (m, 2H), 7.86–7.75 (m, 1H), 7.75–7.67 (m, 1H), 7.62 (d, *J* = 1.9 Hz, 1H), 6.86 (d, *J* = 9.4 Hz, 1H), 6.82–6.68 (m, 2H), 2.90–2.79 (m, 5H), 2.76–2.67 (m, 2H), 1.71–1.55 (m, 2H), 1.47–1.20 (m, 13H).

##### 2-(1-(2-(Methylamino)-5-nitrophenyl)-1*H*-imidazole-4-yl)-5-(trifluoromethyl)­phenyl 6-(2-((4-Oxo-3-phenyl-4,5-dihydro-3*H*-pyrimido­[5,4-*b*]­indol-2-yl)­thio)­acetamido)­hexanoate (**T1/2/T4**)

Compound **5** (35 mg, 0.0594 mmol, 1 equiv) was deprotected using general procedure A and coupled to **T4** (23 mg, 0.0653 mmol, 1.1 equiv) using general procedure C (1 M HCl was not used for extraction). The crude product was purified by flash chromatography (hexane/ethyl acetate 1:3) to give compound **T1/2/4** as a white solid (21 mg, 36%). ^1^H NMR (400 MHz, DMSO-*d*_6_) δ: 12.16 (s, 1H), 8.37 (d, *J* = 8.2 Hz, 1H), 8.29–8.21 (m, 2H), 8.12–8.03 (m, 3H), 7.82 (d, *J* = 1.2 Hz, 1H), 7.75 (dd, *J* = 8.4, 1.9 Hz, 1H), 7.70–7.60 (m, 4H), 7.57–7.46 (m, 4H), 7.30–7.25 (m, 1H), 6.87 (d, *J* = 9.4 Hz, 1H), 6.83–6.74 (m, 1H), 3.93 (s, 2H), 3.10 (q, *J* = 6.7 Hz, 2H), 2.83 (d, *J* = 4.7 Hz, 3H), 2.64 (t, *J* = 7.5 Hz, 2H), 1.66–1.56 (m, 2H), 1.50–1.41 (m, 2H), 1.38–1.30 (m, 2H). ^13^C NMR (100 MHz, DMSO) δ: 171.93, 167.16, 155.42, 152.89, 150.73, 146.98, 139.40, 139.13, 137.70, 136.52, 135.92, 135.55, 131.09, 130.31, 130.01, 128.89, 127.71, 127.12, 124.32, 123.12, 123.08, 122.97, 121.51, 121.39, 121.27, 120.85, 120.76, 120.52, 119.73, 113.29, 110.48, 55.38, 39.22, 36.99, 33.99, 30.22, 29.27, 26.20, 24.15. HRMS *m*/*z* calculated for C_41_H_36_O_6_N_8_F_3_S, 825.2425 (M + H)^+^; found, 825.2411.

##### 4-((6-(2-(1-(2-(Methylamino)-5-nitrophenyl)-1*H*-imidazole-4-yl)-5-(trifluoromethyl)­phenoxy)-6-oxohexyl)­amino)-4-oxobutanoic Acid (**6**)

Compound **5** (110 mg, 0.187 mmol, 1 equiv) was deprotected using general procedure A. The resulting intermediate was dissolved in DMF (1 mL). To an ice-chilled solution, succinic anhydride (22 mg, 0.224 mmol, 1.2 equiv), Et_3_N (101 μL, 0.746 mmol, 4 equiv), and DMAP (catalytic amount) were added, and stirring continued overnight at room temperature. Subsequently, DCM (20 mL) was added, and the mixture was extracted twice with saturated NaHCO_3_. Combined water phases were acidified with 1 M HCl to pH 5. The product was extracted with ethyl acetate, dried over anhydrous Na_2_SO_4_, and concentrated in vacuo to produce compound **6**. Yield (100 mg, 89%). ^1^H NMR (400 MHz, DMSO-*d*_6_) δ: 12.05 (s, 1H), 8.35–8.29 (m, 1H), 8.23 (dd, *J* = 9.3, 2.6 Hz, 1H), 8.05–7.99 (m, 2H), 7.82–7.73 (m, 2H), 7.73–7.67 (m, 1H), 7.66–7.58 (m, 1H), 6.91–6.82 (m, 1H), 6.81–6.71 (m, 1H), 2.98 (q, *J* = 6.4 Hz, 2H), 2.81 (d, *J* = 4.8 Hz, 3H), 2.72–2.66 (m, 2H), 2.43–2.34 (m, 2H), 2.27 (t, *J* = 6.8 Hz, 2H), 1.69–1.55 (m, 2H), 1.43–1.26 (m, 4H).

##### Dicyclopentyl ((*E*)-3-(3-Methoxy-4-((4-((6-(2-(1-(2-(Methylamino)-5-nitrophenyl)-1*H*-imidazole-4-yl)-5-(trifluoromethyl)­phenoxy)-6-oxohexyl)­amino)-4-oxobutanoyl)­oxy)­phenyl)­acryloyl)­glycyl-l-valyl-d-glutamate (**T1/2/N2**)

This was synthesized from **6** (100 mg, 0.166 mmol, 1.1 equiv) and **N2** (93 mg, 0.151 mmol, 1.0 equiv) using general procedure B. The crude product was purified by Isolera One flash chromatography (acetonitrile/0.1% TFA 20% → 100%) to obtain compound **T1/2/N2** as a pale-yellow solid (25 mg, 14%). ^1^H NMR (400 MHz, DMSO-*d*_6_) δ: 8.42–8.26 (m, 3H), 8.26–8.16 (m, 1H), 8.05–7.98 (m, 2H), 7.96 (d, *J* = 9.0 Hz, 1H), 7.88 (t, *J* = 5.6 Hz, 1H), 7.80 (d, *J* = 1.3 Hz, 1H), 7.74–7.66 (m, 1H), 7.66–7.60 (m, 1H), 7.42 (d, *J* = 15.7 Hz, 1H), 7.33 (d, *J* = 1.8 Hz, 1H), 7.17 (dd, *J* = 8.3, 1.8 Hz, 1H), 7.09 (d, *J* = 8.2 Hz, 1H), 6.86 (d, *J* = 9.4 Hz, 1H), 6.80–6.70 (m, 2H), 5.10–5.01 (m, 2H), 4.29–4.17 (m, 2H), 3.91 (d, *J* = 5.6 Hz, 2H), 3.80 (s, 3H), 3.02 (q, *J* = 6.5 Hz, 2H), 2.81 (d, *J* = 4.8 Hz, 3H), 2.73 (dt, *J* = 20.3, 7.3 Hz, 4H), 2.47–2.41 (m, 2H), 2.32 (t, *J* = 8.0 Hz, 2H), 2.04–1.89 (m, 2H), 1.86–1.71 (m, 5H), 1.69–1.49 (m, 14H), 1.42–1.25 (m, 4H), 0.85 (t, *J* = 6.3 Hz, 6H). ^13^C NMR (100 MHz, DMSO) δ: 172.18, 172.03, 171.77, 171.47, 171.13, 170.60, 169.20, 165.66, 161.28, 159.60, 151.52, 150.76, 146.99, 140.77, 140.70, 139.16, 138.88, 135.93, 135.55, 134.26, 131.10, 128.88, 124.35, 123.72, 123.09, 121.56, 121.54, 121.41, 120.56, 112.14, 112.11, 110.52, 108.02, 77.67, 76.93, 57.82, 56.27, 51.62, 38.76, 34.07, 32.62, 32.59, 32.48, 31.29, 30.41, 30.35, 30.23, 29.27, 26.37, 26.23, 24.19, 23.71, 23.66, 19.56, 18.33. HRMS *m*/*z* calculated for C_59_H_72_O_15_N_8_F_3_, 1189.5064 (M + H)^+^; found, 1189.5062.

##### 2-(1-(2-(Methylamino)-5-nitrophenyl)-1*H*-imidazole-4-yl)-5-(trifluoromethyl)­phenyl 6-(4-((6-Amino-2-butoxy-8-hydroxy-9*H*-purin-9-yl)­methyl)­benzamido)­hexanoate (**T1/2/T7**)

Compound **5** (95 mg, 0.161 mmol, 1 equiv) was deprotected using general procedure A and coupled to **T7** (69 mg, 0.193 mmol, 1.2 equiv) using general procedure B. The crude product was purified by flash chromatography (dichloromethane/methanol 15:1) to give compound **T1/2/T7** as a white solid (39 mg, 29%). ^1^H NMR (400 MHz, DMSO-*d*_6_) δ: 9.98 (s, 1H), 8.37 (t, *J* = 5.7 Hz, 1H), 8.31 (d, *J* = 8.3 Hz, 1H), 8.21–8.14 (m, 1H), 8.03–7.97 (m, 2H), 7.95 (s, 1H), 7.80 (d, *J* = 1.3 Hz, 1H), 7.74 (d, *J* = 8.3 Hz, 2H), 7.69 (d, *J* = 8.9 Hz, 1H), 7.62 (d, *J* = 1.8 Hz, 1H), 7.33 (d, *J* = 8.2 Hz, 2H), 6.79 (d, *J* = 9.4 Hz, 1H), 6.77–6.72 (m, 1H), 6.47 (s, 2H), 4.90 (s, 2H), 4.12 (t, *J* = 6.6 Hz, 2H), 2.89 (s, 4H), 2.77 (d, *J* = 4.8 Hz, 2H), 2.73 (s, 2H), 2.73 (s, 4H), 1.67–1.59 (m, 3H), 1.53–1.46 (m, 2H), 1.38–1.34 (m, 3H), 1.28–1.23 (m, 5H), 0.89 (t, *J* = 7.4 Hz, 3H). ^13^C NMR (100 MHz, DMSO) δ: 172.02, 166.19, 160.57, 152.69, 150.72, 149.60, 148.29, 146.99, 140.51, 139.13, 135.93, 135.54, 130.03, 128.86, 127.84, 127.81, 127.79, 127.64, 127.11, 124.31, 121.54, 121.40, 110.44, 98.75, 66.30, 42.58, 34.07, 31.04, 30.19, 29.31, 29.24, 26.26, 24.21, 19.20, 14.16. HRMS *m*/*z* calculated for C_40_H_42_F_3_O_7_N_10_, 831.3185 (M + H)^+^; found, 831.3186.

##### *tert*-Butyl (2-(2-(2-(2-(2-Naphthamido)­benzo­[*d*]­thiazole-6-carboxamido)­ethoxy)­ethoxy)­ethyl)­carbamate (**7**)

This was synthesized from **RI** (120 mg, 0.344 mmol, 1 equiv) and **10** (155 mg, 0.447 mmol, 1.3 equiv) using general procedure B. Yield (160 mg, 69%) ^1^H NMR (400 MHz, DMSO-*d*_6_) δ: 13.21 (s, 1H), 8.93–8.81 (m, 1H), 8.60 (t, *J* = 5.6 Hz, 1H), 8.56–8.45 (m, 1H), 8.22–8.15 (m, 1H), 8.15–8.07 (m, 2H), 8.07–8.01 (m, 1H), 7.97 (dd, *J* = 8.4, 1.8 Hz, 1H), 7.89–7.78 (m, 1H), 7.77–7.56 (m, 2H), 6.78 (s, 1H), 3.63–3.35 (m, 10H), 3.06 (q, *J* = 6.0 Hz, 2H), 1.36 (s, 9H).

##### 2-(2-Naphthamido)-*N*-(2-(2-(2-(2-((4-oxo-3-phenyl-4,5-dihydro-3*H*-pyrimido­[5,4-*b*]­indol-2-yl)­thio)­acetamido)­ethoxy)­ethoxy)­ethyl)­benzo­[*d*]­thiazole-6-carboxamide (**T4/RI**)

Compound **7** (80 mg, 0.138 mmol, 1 equiv) was deprotected using general procedure A and coupled to **T4** (53 mg, 0.152 mmol, 1.2 equiv) using general procedure B. The crude product was purified by flash chromatography (dichloromethane/methanol 40:1) to give compound **T4/RI** as a brown solid (55 mg, 49%). ^1^H NMR (400 MHz, DMSO-*d*_6_) δ: 13.20 (s, 1H), 12.10 (s, 1H), 8.91–8.80 (m, 1H), 8.59 (t, *J* = 5.4 Hz, 1H), 8.53 (s, 1H), 8.32 (t, *J* = 5.7 Hz, 1H), 8.20–8.13 (m, 1H), 8.13–8.07 (m, 2H), 8.07–8.00 (m, 2H), 8.00–7.91 (m, 1H), 7.89–7.81 (m, 1H), 7.75–7.63 (m, 2H), 7.63–7.54 (m, 3H), 7.54–7.39 (m, 4H), 7.28–7.19 (m, 1H), 3.91 (s, 2H), 3.56–3.39 (m, 10H), 3.24 (q, *J* = 5.8 Hz, 2H). ^13^C NMR (100 MHz, DMSO) δ: 167.60, 166.41, 155.42, 152.82, 139.39, 137.70, 136.51, 135.34, 132.44, 130.33, 130.10, 130.01, 129.81, 129.10, 128.81, 128.22, 127.77, 127.59, 126.00, 124.79, 121.71, 120.86, 120.80, 120.62, 119.72, 113.29, 70.05, 69.95, 69.46, 69.42, 55.39, 36.89. HRMS *m*/*z* calculated for C_43_H_38_O_6_N_7_S_2_, 812.2320 (M + H)^+^; found, 812.2312.

##### Ethyl 6-(2-(2-Naphthamido)­benzo­[*d*]­thiazole-6-carboxamido)­hexanoate (**8**)

This was synthesized from **RI** (900 mg, 2.583 mmol, 1 equiv) and **10** (607 mg, 3.100 mmol, 1.2 equiv) using general procedure B. Yield (0.935 mg, 75%). ^1^H NMR (400 MHz, DMSO-*d*_6_) δ: 8.88–8.80 (m, 1H), 8.54 (t, *J* = 5.5 Hz, 1H), 8.50–8.43 (m, 1H), 8.20 (dd, *J* = 8.6, 1.8 Hz, 1H), 8.14–7.99 (m, 3H), 7.93 (dd, *J* = 8.5, 1.8 Hz, 1H), 7.81–7.74 (m, 1H), 7.71–7.57 (m, 2H), 4.11–3.97 (m, 2H), 3.32–3.18 (m, 2H), 2.35–2.23 (m, 2H), 1.68–1.45 (m, 4H), 1.45–1.20 (m, 2H), 1.20–1.11 (m, 3H).

##### 6-(2-(2-Naphthamido)­benzo­[*d*]­thiazole-6-carboxamido)­hexanoic Acid (**9**)

To a stirring solution of compound **8** (0.935 mg, 1.912 mmol, 1 equiv) in methanol (15 mL) was added 1 M NaOH (15 mL). The mixture was stirred at room temperature for 2.5 h. Subsequently, methanol was evaporated in vacuo, and the water phase was washed with ethyl acetate (30 mL) and acidified with 1 M HCl to pH 5. The product was extracted two times with ethyl acetate (20 mL). Yield (454 mg, 52%). ^1^H NMR (400 MHz, DMSO-*d*_6_) δ: 8.73 (s, 1H), 8.36–8.28 (m, 2H), 8.18 (d, *J* = 1.8 Hz, 1H), 8.08–7.98 (m, 1H), 7.98–7.86 (m, 2H), 7.77–7.67 (m, 1H), 7.60–7.47 (m, 1H), 7.47–7.39 (m, 1H), 3.28–3.21 (m, 3H), 2.24–2.09 (m, 2H), 1.62–1.44 (m, 4H), 1.41–1.21 (m, 2H).

##### 2-(1-(2-(Methylamino)-5-nitrophenyl)-1*H*-imidazole-4-yl)-5-(trifluoromethyl)­phenyl 6-(2-(2-Naphthamido)­benzo­[*d*]­thiazole-6-carboxamido)­hexanoate (**T1/2/RI**)

This was synthesized from **9** (90 mg, 0.195 mmol, 1.1 equiv) and **T1/2** (66 mg, 0.177 mmol, 1 equiv) using general procedure B. The crude product was purified by flash chromatography (dichloromethane/methanol 40:1) and Isolera One flash chromatography (acetonitrile/0.1% TFA 20% → 100%) to give compound **T1/2/RI** as a pale-yellow solid. Yield (20 mg, 14%). ^1^H NMR (400 MHz, DMSO-*d*_6_) δ: 13.20 (s, 1H), 8.93–8.80 (m, 1H), 8.58–8.37 (m, 2H), 8.37–8.25 (m, 1H), 8.22–8.14 (m, 2H), 8.14–8.09 (m, 2H), 8.07–7.99 (m, 3H), 7.94 (dd, *J* = 8.5, 1.8 Hz, 1H), 7.90–7.74 (m, 2H), 7.74–7.60 (m, 4H), 6.87–6.69 (m, 2H), 3.30–3.22 (m, 2H), 2.87–2.70 (m, 5H), 1.79–1.65 (m, 2H), 1.64–1.50 (m, 2H), 1.49–1.33 (m, 2H). ^13^C NMR (100 MHz, DMSO) δ: 172.06, 166.16, 150.74, 146.99, 139.15, 135.94, 135.54, 135.33, 132.45, 131.12, 130.59, 130.09, 129.80, 129.07, 128.88, 128.80, 128.22, 127.86, 127.58, 127.58, 127.54, 127.11, 125.96, 125.68, 124.81, 124.32, 123.13, 123.09, 121.56, 121.41, 121.34, 121.32, 110.46, 34.09, 30.23, 29.30, 26.32, 24.25. HRMS *m*/*z* calculated for C_42_H_35_O_6_N_7_F_3_S_2_, 822.2316 (M + H)^+^; found, 822.2307.

##### Dicyclopentyl ((*E*)-3-(4-((6-(2-(2-Naphthamido)­benzo­[*d*]­thiazole-6-carboxamido)­hexanoyl)­oxy)-3-methoxyphenyl)­acryloyl)­glycyl-l-valyl-d-glutamate (**N2/RI**)

This was synthesized from **9** (110 mg, 0.239 mmol, 1.1 equiv) and **N2** (134 mg, 0.217 mmol, 1 equiv) using general procedure B. The crude product was purified Isolera One flash chromatography (acetonitrile/0.1% TFA 20% → 100%) to obtain compound **N2/RI** as a pale-yellow solid. Yield (42 mg, 18%). ^1^H NMR (400 MHz, DMSO-*d*_6_) δ: 13.19 (s, 1H), 8.83 (s, 1H), 8.49 (t, *J* = 5.7 Hz, 1H), 8.46–8.41 (m, 1H), 8.37 (d, *J* = 7.4 Hz, 1H), 8.31 (t, *J* = 5.8 Hz, 1H), 8.21 (dd, *J* = 8.6, 1.8 Hz, 1H), 8.13–7.85 (m, 5H), 7.78–7.69 (m, 1H), 7.69–7.57 (m, 2H), 7.42 (d, *J* = 15.7 Hz, 1H), 7.33 (d, *J* = 1.8 Hz, 1H), 7.19–7.06 (m, 2H), 6.77 (d, *J* = 15.8 Hz, 1H), 5.23–4.86 (m, 2H), 4.32–4.12 (m, 2H), 3.90 (d, *J* = 5.7 Hz, 2H), 3.80 (s, 3H), 2.60 (t, *J* = 7.2 Hz, 2H), 2.31 (t, *J* = 7.4 Hz, 2H), 2.04–1.86 (m, 3H), 1.84–1.36 (m, 24H), 0.92–0.73 (m, 6H). ^13^C NMR (100 MHz, DMSO) δ: 172.17, 171.76, 171.53, 171.46, 169.21, 166.47, 165.68, 151.54, 140.72, 138.87, 134.99, 134.30, 132.66, 132.31, 129.64, 129.53, 128.36, 128.34, 128.27, 128.12, 127.16, 125.44, 125.40, 123.69, 122.61, 121.11, 120.58, 119.37, 112.10, 77.66, 76.92, 57.83, 56.25, 51.63, 33.66, 32.62, 32.59, 32.48, 31.29, 30.43, 29.36, 26.39, 26.30, 24.79, 23.71, 23.66, 19.56, 18.34. HRMS *m*/*z* calculated for C_57_H_67_O_12_N_6_S, 1059.4532 (M + H)^+^; found, 1059.4524.

### Mice

#### Experiments with Bone-Marrow-Derived DCs and T Cells

C57BL/6, OT I (C57BL/6-Tg­(TcraTcrb)­1100Mjb/J), and OT II (C57BL/6-Tg­(TcraTcrb)­425Cbn/Crl) mice were obtained from Jackson Laboratory (Bar Harbor, ME) and bred at the University of Leiden (The Netherlands). The mice were housed under standard laboratory conditions with unrestricted access to food and water. Euthanasia was performed under sedation via cervical dislocation. All animal procedures complied with European Parliament Directive 2010/63/EU and were approved by the Animal Ethics Committee of Leiden University. Details on the BMDC culture conditions are provided below.

#### In Vivo Vaccination Model

NIH/OlaHsd inbred female mice (2.0–2.5 months old) were bred at the Institute of Immunology, Croatia. During the experiments, they were housed in the institute’s Animal Facility with unrestricted access to food and water. All animal procedures adhered to the Croatian Law on Animal Welfare (2017), in full compliance with the EC Directive 2010/63/EU. Ethical approval was obtained from the Ethical Committee of the Institute of Immunology and the Directorate of Veterinary and Food Safety of the Ministry of Agriculture, Republic of Croatia, approval number: HR-POK-009.

#### In Vivo Tumor Model

Female C57Bl/6 mice (9 weeks old) were obtained from Bienta’s Animal Facility. All in vivo procedures were approved by Bienta’s Animal Care and Use Committee (BACUC, Approval No. LU-EF-AP-0609624) and conducted in accordance with the European Convention for the Protection of Vertebrate Animals Used for Experimental and Other Scientific Purposes. Mice were housed under specific pathogen-free conditions with a 12 h light/dark cycle and had unrestricted access to food and water in Bienta’s animal research center laboratory.

### Cell Culture

#### HEK-Blue NOD2, TLR2, TLR4, and TLR7 Cells and HEK-Lucia RIG-I Cells

HEK-Blue NOD2 (Cat. code: hkb-hnod2), TLR2 (Cat. code: hkb-htlr2), TLR4 (Cat. code: hkb-htlr4), and TLR7 (Cat. code: hkb-htlr7) cell lines (Invivogen, San Diego, CA) are derived from HEK293 cells by cotransfection of hNOD2, hTLR2, hTLR4, or hTLR7 genes, respectively, and a nuclear factor-κB (NF-κB)-inducible secreted embryonic alkaline phosphatase (SEAP) reporter gene. Following the activation of the respective receptors, the resulting NF-κB induces the production of SEAP, the levels of which can be quantified colorimetrically. HEK-Lucia RIG-I cells (cat. code: hkl-hrigi) are derived from HEK293 cells by cotransfection of hRIG-I gene and an IFN-inducible Lucia luciferase reporter gene. Following activation, the resulting IFN induces Lucia luciferase activity, which can be quantified colorimetrically. HEK-Blue cells and HEK-Lucia cells were cultured according to the manufacturer’s instructions, in Dulbecco’s modified Eagle’s medium (Sigma-Aldrich, St. Louis, MO) supplemented with 10% heat-inactivated fetal bovine serum (Gibco), 2 mM l-glutamine (Sigma-Aldrich), 100 U/mL penicillin (Sigma-Aldrich), 100 μg/mL streptomycin (Sigma-Aldrich), and 100 μg/mL Normocin (Invivogen) for two passages. All of the subsequent passages were cultured in the medium additionally supplemented with 100 μg/mL Zeocin (Invivogen) and 30 μg/mL Blasticidin (Invivogen) for HEK-Blue hNOD2 cells and HEK-Lucia RIG-I cells with 100 μg/mL Zeocin (Invivogen) and 10 μg/mL Blasticidin (Invivogen) for HEK-Blue hTLR7, or 1x HEK-Blue Selection for HEK-Blue hTLR2 and HEK-Blue hTLR4 cells. The cells were incubated in a humidified atmosphere at 37 °C and 5% CO_2_.

#### Peripheral Blood Mononuclear Cells

Human PBMCs were isolated from buffy coats of healthy, consenting donors using density gradient centrifugation with Ficoll–Paque (Pharmacia, Sweden). Buffy coats from venous blood of normal healthy volunteers were obtained from the Blood Transfusion Centre of Slovenia, consistent with institutional guidelines and approval of the National Medical Ethics Committee of Slovenia (Number 0120-279/2017-3). The cells were washed twice with PBS, resuspended in RPMI 1640 medium (Sigma-Aldrich, St. Louis, MO) supplemented with 10% heat-inactivated fetal bovine serum (Gibco), 2 mM l-glutamine (Sigma-Aldrich), 100 U/mL penicillin (Sigma-Aldrich), and 100 μg/mL streptomycin (Sigma-Aldrich), and subsequently used in assays.

#### Cancer Cell Line

K562, a chronic myelogenous leukemia cell line (Cat. No. CCL-243; ATCC, Manassas, VA), was cultured in RPMI 1640 medium (Sigma-Aldrich, St. Louis, MO) supplemented with 10% heat-inactivated fetal bovine serum (Gibco), 2 mM l-glutamine (Sigma-Aldrich), 100 U/mL penicillin (Sigma-Aldrich), and 100 μg/mL streptomycin (Sigma-Aldrich). The cells were incubated in a humidified atmosphere at 37 °C and 5% CO_2_.

#### Bone-Marrow-Derived DCs

Bone-marrow cells were harvested from the tibiae of C57BL/6 mice and cultured in RPMI 1640 with HEPES (Lonza, Basel, Switzerland), supplemented with 10% heat-inactivated fetal bovine serum (Lonza), 2 mM l-glutamine (Lonza), 100 U/mL penicillin (Lonza), 100 μg/mL streptomycin (Lonza), 50 μM β-mercaptoethanol (Lonza), and 20 ng/mL granulocyte–macrophage colony-stimulating factor (ImmunoTools, Friesoythe, Germany) for 10 days at 37 °C and 5% CO_2_. BMDC purity was assessed via flow cytometry using a PE-labeled antimouse CD11c antibody (BioLegend, San Diego, CA), with >90% of cells confirmed as CD11c-positive.

### Measurement of NF-κB Transcriptional Activity in HEK-Blue Cells

HEK-Blue hNOD2, hTLR2, hTLR4, or hTLR7 cells were seeded (25,000 cells/well) in duplicate in 96-well plates in 100 μL of HEK-Blue detection medium (Invivogen, San Diego, CA) and treated in duplicates with the compounds (1 or 10 μM for fixed concentration assays; eight different concentrations ranging from 1 nM to 10 μM for hNOD2 cells, 0.1 nM to 1 μM for hTLR2 and hTLR7 cells, or 1.6 nM to 100 μM for hTLR4 cells for the determination of EC_50_) or with the corresponding vehicle (0.1% DMSO). After 18 h of incubation (37 °C, 5% CO_2_), SEAP activity was determined spectrophotometrically as the absorbance at 630 nm (BioTek Synergy microplate reader; Winooski, VT). EC_50_ values were calculated using Prism software (version 10; GraphPad Software, CA).

### Measurement of IRF-Induced Lucia Luciferase Activity in HEK-Lucia Cells

HEK-Lucia RIG-I cells were seeded (50,000 cells/well) in 96-well plates in 100 μL of growth medium without added selection antibiotics and treated in duplicate with the compounds (1 or 10 μM for fixed concentration assays; eight different concentrations ranging from 1.6 nM to 100 μM for the determination of EC_50_) or with the corresponding vehicle (0.1% DMSO). After 18 h of incubation (37 °C, 5% CO_2_), 10 μL of supernatants was transferred to 96-well white opaque plates. 50 μL of Quanti-Luc 4 detection medium (Invivogen, San Diego, CA) was added, and the activity of IRF-induced Lucia luciferase was immediately determined luminometrically (BioTek Synergy microplate reader; Winooski, VT).

### Cytokine Release from PBMCs

PBMCs were seeded (1 × 10^6^ cells/mL) in 24-well plates in 500 μL of growth medium and treated with conjugated agonists (1 μM), individual agonists (1 μM), unlinked mixtures of agonists (1 μM), lipopolysaccharide (LPS; 1 μg/mL), or the corresponding vehicle (0.1% DMSO). For degradation product studies, PBMCs were treated with conjugated agonists **T4/T7** and **T7/RI** (0.1 and 1 μM), their corresponding degradation products (0.1 and 1 μM), or the corresponding vehicle (0.1% DMSO). For mechanistic studies of **T4/T7** and **T7/RI**, PBMCs were pretreated with (a) TLR4 antagonist TAK242 (5 μM), TLR7 antagonist M5049 (5 μM), or both for 1 h, before the addition of **T4/T7** (100 nM); (b) TLR7 antagonist M5049 (5 μM), RIG-I signaling inhibitor MRT67307 (5 μM), or both for 1 h, before the addition of **T7/RI** (100 nM) or (c) the corresponding vehicle (0.1% DMSO). For endocytosis studies of **T4/T7**, PBMCs were pretreated with Dynasore (80 μM) for 1 h, before the addition of **T4/T7** (1 μM) or the corresponding vehicle (0.1% DMSO). Cell-free supernatants were collected after 18 h of incubation (37 °C, 5% CO_2_) and stored at −80 °C until tested. Cytokine production was determined with a LEGENDplex Human Essential Immune Response Panel (Biolegend, San Diego, CA) (comprising IL-4, IL-2, CXCL10 (IP-10), IL-1β, TNF-α, CCL2 (MCP-1), IL-17A, IL-6, IL-10, IFN-γ, IL-12p70, CXCL8 (IL-8), TGF-β1) on an Attune NxT flow cytometer (Thermo Fisher Scientific, Waltham, MA). Standard curves were generated using recombinant cytokines contained in the kit. The data were analyzed using FlowJo (Tree Star, Inc., Ashland, OR) and Prism (GraphPad, San Diego, CA) software. Statistical significance was determined using one-way ANOVA with posthoc Bonferroni’s test.

### RNA Sequencing

PBMCs from three independent donors were seeded in 24-well plates at a density of 3 × 10^6^ cells/mL in 1 mL of growth medium. Cells were treated with either conjugates (1 μM) or a vehicle control (0.1% DMSO) for 18 h at 37 °C with 5% CO_2_. Following treatment, cells were washed with PBS, resuspended in RNAlater RNA stabilization solution (Sigma-Aldrich, St. Louis, MO) and stored at −80 °C for subsequent RNA extraction.

RNA extraction, library construction, and sequencing were conducted by Azenta Life Sciences (Leipzig, Germany). Total RNA was extracted using RNeasy mini kit (Qiagen, Hilden, Germany), following the manufacturer’s protocol. RNA samples were quantified using a Qubit 4.0 fluorometer (Life Technologies, Carlsbad, CA), and RNA integrity was verified with an RNA kit on a 5300 Fragment Analyzer (Agilent Technologies, Palo Alto, CA), ensuring all samples had an RNA quality number ≥9.4. RNA sequencing libraries were prepared using the NEBNext Ultra II RNA library prep kit for Illumina, according to the manufacturer’s instructions (New England Biolabs, Ipswich, MA). Libraries were loaded on the Illumina NovaSeq 6000 instrument, and clustering was performed directly on the NovaSeq before sequencing according to the manufacturer’s instructions. The samples were sequenced using a 2 × 150 paired-end configuration. Image analysis and base calling were conducted using the NovaSeq Control Software. Raw sequencing data (.bcl files) generated from the Illumina NovaSeq were converted into fastq format and demultiplexed using the Illumina bcl2fastq 2.20 software, allowing for one mismatch in index sequence identification. After investigating the quality of the raw data, sequence reads were trimmed to remove possible adapter sequences and nucleotides with poor quality using Trimmomatic v.0.36. The trimmed reads were mapped to the *Homo sapiens* reference genome, as available on ENSEMBL, using STAR aligner v.2.5.2b, thus generating BAM files. Unique gene hit counts were calculated using feature counts from the Subread package v.1.5.2. Only unique reads that fell within the exon regions were counted. After the extraction of gene hit counts, the gene hit count table was used for the downstream differential expression analysis.

Differential gene expression analysis was conducted using iDEP 2.0.^51^ First, low-expressed genes were filtered out (retaining those with ≥0.5 counts per million in at least two libraries). The remaining gene counts were normalized by counts per million in the EdgeR package, with a pseudocount of 4. Differential gene expression analysis was carried out with the DESeq2 method, applying a false-discovery rate threshold of <0.1 and a fold-change cutoff of >2 or <0.5. The resulting list of differentially expressed genes was used for gene annotation and pathway enrichment analysis with Metascape.^52^

### PBMC Cytotoxicity Assay

The PBMC cytotoxicity assay using K562 cells was performed as described previously, with some modifications.^53^ PBMCs were seeded (4 × 10^5^ cells/well) in duplicate in 96-well U-bottom plates and treated with conjugated agonists (1 μM), individual agonists (1 μM), unlinked mixtures of agonists (1 μM), or a vehicle (0.1% DMSO) for 18 h. For mechanistic studies of T4/T7 and T7/RI, PBMCs were pretreated with (a) TLR4 antagonist TAK242 (5 μM), TLR7 antagonist M5049 (5 μM), or both for 1 h, before the addition of **T4/T7** (100 nM); (b) TLR7 antagonist M5049 (5 μM), RIG-I signaling inhibitor MRT67307 (5 μM), or both for 1 h, before the addition of **T7/RI** (100 nM), or (c) the corresponding vehicle (0.1% DMSO). K562 cells were stained with CFSE (Invitrogen, Carlsbad, CA), washed twice with complete medium, and added (1 × 10^4^ cells/well) to the pretreated PBMCs for a final effector cell/target tumor cell ratio of 40:1. After a 4 h coincubation (37 °C, 5% CO_2_), the cells were stained with Sytox blue dead cell stain (Invitrogen) and analyzed using an Attune NxT flow cytometer (Thermo Fisher Scientific, Waltham, MA) and FlowJo software (Tree Star, Inc., Ashland, OR). Cells that were positive for both CFSE and Sytox blue were defined as dead K562 cells. PBMCs alone and CFSE-labeled cancer cells alone were also treated with the compounds at the same concentrations and stained with Sytox blue to exclude any direct cytotoxicity of the compounds toward the PBMCs and cancer cells. Statistical significance was determined using one-way ANOVA with posthoc Bonferroni’s test.

### Bone-Marrow-Derived DC Antigen Presentation to CD4^+^ and CD8^+^ T Cells

CD4^+^ and CD8^+^ T cells were purified from the splenocytes of OT-II and OT-I transgenic mice using CD4^+^ and CD8^+^ T-cell-negative selection kits (Miltenyi Biotec, Germany), according to the manufacturer’s instructions. The purified T cells were stained with 0.5 μM CFSE in PBS at 1 × 10^6^ cells/mL (Invitrogen, Carlsbad, CA) and washed. Then, 5 × 10^4^ T cells were cultured in duplicate with 1 × 10^4^ BMDCs/well (pretreated with compounds [1 μM] or LPS [1 μg/mL] and 50 μg/mL ovalbumin (OVA)-soluble protein [Invivogen, San Diego, CA] for 18 h and then washed). After being coincubated for 72 h (37 °C, 5% CO_2_), the supernatants were collected and stored at −80 °C for subsequent cytokine measurements. The cells were stained with Fixable viability dye APC-eFluor 780 (eBioscience, Thermo Fisher Scientific, MA), anti-Thy1.2 PE-Cy7 (Biolegend, San Diego, CA), anti-CD8 eFluor450 (eBioscience), anti-CD4 eFluor450 (eBioscience), and anti-CD25 APC antibodies (Biolegend) and analyzed using a Beckman Coulter Cytoflex S flow cytometer (CA) and the FlowJo software (Tree Star, Inc., Ashland, OR). Live Thy1.2^+^/CD4^+^ and Thy1.2^+^/CD8^+^ were evaluated for CFSE dilution and CD25 expression. Supernatants from T cells and BMDC cocultures were collected as described above. The cytokine concentrations were determined with the LEGENDplex MU Th Cytokine Panel (12-plex) (Biolegend, San Diego, CA) (comprising IFN-γ, IL-5, TNF-α, IL-2, IL-6, IL-4, IL-10, IL-9, IL-17A, IL-17F, IL-22, and IL-13) on an Attune NxT flow cytometer (Thermo Fisher Scientific, Waltham, MA). Standard curves were generated using recombinant cytokines contained in the kit. The data were analyzed using the FlowJo (Tree Star, Inc., Ashland, OR) and Prism (GraphPad, San Diego, CA) software. Statistical significance was determined using one-way ANOVA with posthoc Dunnett’s test.

### Mouse Immunizations

Sex-matched NIH/OlaHsd mice (six per group; 60 mice in total for both experiments) were immunized subcutaneously into the tail base on days 0, 21, and 42 with (a) OVA (10 μg; Serva, Germany) alone or plus the conjugates (42 μg (**T4/T7**), 44 μg (**T7/RI**), 44 μg (**T1/2/T7**), and 64 μg (**T1/2/N2**)—doses are equimolar to 50 μg of **N2/T7**) and Al-hydrogel (100 μg; Invivogen, San Diego, CA) or (b) OVA (10 μg) alone or plus the conjugate **T4/T7** (42 μg), MDP (148 μg; Invivogen, San Diego, CA), and imiquimod (72 μg; Cat. Code: vac-imq; Invivogen, San Diego, CA). All experimental groups received an injection volume of 0.1 mL per mouse. Seven days after the second booster dose, mice were anesthetized via intraperitoneal administration of ketamine/xylazine (25 mg/kg each) before blood collection from the axillary plexus. Serum samples were individually processed by heat inactivation at 56 °C for 35 min to remove complement activity and subsequently stored at −20 °C until analysis.

### Measurement of Ovalbumin-Specific Serum Antibody Concentration

The levels of the OVA-specific total IgG, IgG1, and IgG2a in mouse sera were quantified using ELISA. High-binding ELISA plates (Costar) were coated with the OVA (1.5 mg/mL; Serva, Germany) in carbonate buffer (pH 9.6) and incubated overnight. To prevent nonspecific binding, plates were blocked with 0.5% (w/v) bovine serum albumin in PBS-T (0.05% [v/v] Tween 20 in PBS) for 2 h at 37 °C. After washing, five serial dilutions of mouse sera and standard preparations were added in duplicate. The plates were incubated overnight at room temperature, followed by washing and detection using HRP-conjugated goat antimouse IgG (Bio-Rad Laboratories) for 2 h at 37 °C. After further washing, the reaction was developed with 0.6 mg/mL *o*-phenylenediamine dihydrochloride in citrate-phosphate buffer (pH 5.0) containing 0.5 μL/mL of 30% H_2_O_2_ for 30 min at room temperature in the dark. The reaction was terminated with 12.5% H_2_SO_4_, and the absorbance at 492 nm was measured using a microplate reader (Thermo Fisher Scientific, Waltham, MA).

For the detection of OVA-specific IgG1 and IgG2a, the plates were incubated with biotin-conjugated rat antimouse IgG1 or IgG2a (PharMingen, Becton Dickinson) for 2 h at 37 °C, followed by incubation with streptavidin-peroxidase (Pharmingen) under the same conditions. After washing, the substrate solution was added and incubated for 30 min at room temperature in the dark. The reaction was stopped with 12.5% H_2_SO_4_, and absorbance at 492 nm was recorded. Antibody levels were determined using parallel line assays with standard preparations of anti-OVA IgG (20,000 AU/mL), anti-OVA IgG1 (400,000 AU/mL), and anti-OVA IgG2a (5000 AU/mL). Statistical significance was assessed using one-way ANOVA, followed by Dunnett’s multiple comparisons test.

### Tumor Model

The experiment was conducted following Bienta’s standard operating procedures. B16F10 cells (ATCC: CRL-6475) were cultured in DMEM (4.5 g/L glucose) supplemented with 10% FBS, 100 U/mL penicillin, and 100 μg/mL streptomycin at 37 °C under a 5% CO_2_ atmosphere. Cells were harvested using a 0.05% trypsin–EDTA solution, centrifuged, and resuspended in serum-free DMEM. Cell count and viability were assessed using a hemocytometer and trypan blue exclusion test. The final cell suspension, containing 5 × 10^5^ cells per 50 μL in DPBS, was kept on ice before injection. Each of the 90 female C57BL/6 mice received a subcutaneous inoculation of 5 × 10^5^ B16F10 cells (50 μL) above the upper thigh. Mice were randomized into four groups based on tumor volume and body weight between days 13 and 23 postinoculation. Treatment was initiated when tumor nodules reached an average volume of approximately 100 mm^3^. Mice with tumors outside the predefined volume range were excluded from the study.

Mice received intratumoral injections of vehicle (10% DMSO in PBS) or the compounds **T4/T7**, **T7/RI**, and resiquimod (formulated in 10% DMSO in PBS) at doses of 49 μg, 52 μg, and 20 μg, respectively. Injections were administered into a single tumor site at a volume equivalent to 1/10 of the tumor volume, every two to 3 days, for a total of six injections over 2 weeks.

Animals were weighed upon arrival, on the day of B16F10 cell inoculation, at each treatment session, and daily throughout the treatment and observation period. Body weight changes were expressed as a percentage relative to the initial weight (DPT of 0) for each animal. Tumor size was measured every 2 days using a digital caliper, with two perpendicular diameters recorded: length (*L*, the larger diameter) and width (*W*, the smaller diameter). Tumor volume (*V*) was calculated using the following formula: *V* = (*W* ^2^ × *L*) × 0.5 mm^3^. Mice were euthanized if tumor volumes exceeded 3000 mm^3^ or if tumor sites developed ulcerations. Euthanasia was performed via CO_2_ inhalation, followed by cervical dislocation, in accordance with Bienta’s SOP, by trained personnel. The relative tumor volume (RTV) was calculated using the formula: RTV = (tumor volume on measured day)/(tumor volume on day 0). Tumor growth inhibition (TGI, %) was determined using TGI (%) = [1 – (RTV of treated group)/(RTV of control group)] × 100. Alternatively, the inhibition of tumor growth was calculated as the percentage of tumor volume in the treated group relative to the control group (T/C, %). Euthanized animals were classified as deceased in the survival analysis.

### LC–MS Analysis of Cell Incubation Media and Lysate Samples

#### Liquid Chromatography with Tandem Mass Spectrometry (LC–MS/MS)

For LC–MS/MS quantitation of compounds in cell incubation media and lysate samples, chromatographic separation was conducted using a Vanquish Flex system (Thermo Fisher Scientific) equipped with a Kinetex PS C18 column (100 × 2.1 mm, 2.6 μm particle size; Phenomenex). The column temperature was maintained at 40 °C, and the injection volume was 10 μL. The mobile phase consisted of water/MeCN/formic acid (950:5:1, v/v/v) as solvent A and water/MeCN/formic acid (50:950:1, v/v/v) as solvent B. The following gradient was applied: 0–7.0 min, 5–75% B; 7.0–8.0 min, 75% B; 8.0–8.1 min, 75%–5% B; and 8.1–10.0 min, 5% B. The flow rate was kept constant at 0.50 mL/min throughout the analysis, and the autosampler temperature was maintained at 5 °C. MS/MS analysis was performed by using a TSQ Fortis triple quadrupole mass spectrometer (Thermo Fisher Scientific) operated with a heated electrospray ionization source (H-ESI) in positive ionization mode and single-reaction monitoring. For all analytes, the detector dwell time was 100 ms, and the collision-induced dissociation gas pressure was 2 mTorr, except for compound T4, for which it was 2.5 mTorr. All other MS/MS parameters and chromatographic conditions for each compound are summarized in Table S10. Instrument control, data acquisition, and quantification were performed using Xcalibur software (Thermo Fisher Scientific).

#### Determination of Extra- and Intracellular Concentrations

PBMCs, isolated from healthy donors, were seeded (0.5 × 10^6^ cells/well) in 24-well plates in 700 μL of growth medium and treated with the compounds (1 μM) or the corresponding vehicle (0.1% DMSO). After 18 h, the supernatants and cells were washed with ice-cold PBS and scraped, collected, and centrifuged (1800 rpm, 5 min). The supernatants were stored at −80 °C, while the cell pellets were further washed with ice-cold PBS and centrifuged (1800 rpm, 5 min). Then, 1 mL of the ice-cold extraction solvent MeCN/MeOH (v/v = 1:1) was added, and the cell suspension was incubated at 4 °C for 30 min. Subsequently, cells were transferred to cold microcentrifuge tubes, and supernatants were harvested (12,000*g*, 15 min, 4 °C) and stored at −80 °C. Samples were analyzed with LC–MS.

### Screening against PAINS

All compounds underwent screening against the PAINS filter^54^ conducted using CANVAS software (Schrödinger, Release 2021-2, New York, USA) and successfully passed the filter.

### Statistics

Data analysis was performed using Prism software (version 10; GraphPad Software, CA). Statistical differences were determined as specified in individual experimental procedures above and in the corresponding figure captions.

### Safety Statement

No unexpected or unusually high safety hazards were encountered during the experiments.

## Supplementary Material







## Data Availability

The data underlying this study are available in the manuscript and its Supporting Information.

## Notes

Experimental procedures; HPLC traces of final compounds; HRMS spectra of final compounds; and 1H and 13C NMR spectra of final compounds (PDF)
Molecular formula strings (CSV)
Complete DESeq2 output for transcriptomic analysis of PBMCs treated with conjugated PRR ligands, including gene identifiers, log_2_-fold changes, and adjusted p-values (XLSX)

## Abbreviations

BMDC: bone-marrow-derived dendritic cell
CFSE: carboxyfluorescein succinimidyl ester
COMU: (1-cyano-2-ethoxy-2-oxoethylidenaminooxy)­dimethylamino-morpholino-carbenium hexafluorophosphate
DC: dendritic cell
DIPEA: *N*,*N*-diisopropylethylamine
DMAP: 4-dimethylaminopyridine
DMF: dimethylformamide
EDC: 1-ethyl-3-(3-(dimethylamino)­propyl)­carbodiimide
HATU: *O*-(7-azabenzotriazol-1-yl)-*N*,*N*,*N*′,*N*′-tetramethyluronium hexafluorophosphate
IFN: interferon
IL: interleukin
IP-10: interferon gamma-induced protein 10
IRF: interferon regulatory factor
MCP-1: monocyte chemoattractant protein-1
LPS: lipopolysaccharide
NF-κB: nuclear factor κB
NK: natural killer
NLR: NOD-like receptor
NOD: nucleotide-binding oligomerization domain
OVA: ovalbumin
PAMP: pathogen-associated molecular pattern
PBMC: peripheral blood mononuclear cell
PCA: principal component analysis
PRR: pattern recognition receptor
RIG-I: retinoic acid-inducible gene I
RLR: RIG-I-like receptor
TGF: transforming growth factor
TLR: Toll-like receptor
TNF: tumor necrosis factor
