# Supplementary material for: Probing Immune Signatures of Conjugated Pattern Recognition Receptor Ligands Identifies Chimeras with Potent Adjuvant and Antitumor Activities
Source: J Med Chem. 2026 May 6;69(10):12479–501. doi: 10.1021/acs.jmedchem.6c00372 (PMC13224164; doi:10.1021/acs.jmedchem.6c00372)

SUPPLEMENTARY INFORMATION

Probing immune signatures of conjugated pattern recognition receptor ligands identifies chimeras with potent adjuvant and antitumor activity

Špela Janež,[1] Samo Guzelj,[1] Veronika Weiss,[1] Marcela Šišić,[2] Ruža Frkanec,[2] Stane Pajk,[1] Lenny Burgmeijer,[3] Bram Slütter,[3] Žiga Jakopin*,[1]

[1]Faculty of Pharmacy, University of Ljubljana, SI-1000 Ljubljana, Slovenia

[2]Centre for Research and Knowledge Transfer in Biotechnology, University of Zagreb, 10000 Zagreb, Croatia.

[3]Div. BioTherapeutics, Leiden Academic Centre for Drug Research, Leiden University, 2333 CC Leiden, The Netherlands

*Corresponding Author

Žiga Jakopin

Phone: +386 1 4769 646

Fax: +386 1 4258 031

E-mail: ziga.jakopin@ffa.uni-lj.si

Table of Contents

1. Experimental Procedures 3
2. Supplementary Figures 5
3. Supplementary Schemes 13
4. Supplementary Tables 14
5. HPLC chromatograms/traces of final compounds 27
6. HRMS spectra of final compounds 36
7. 1H and 13C NMR spectra of final compounds 45

1. Experimental Procedures

Synthesis of linkers

*Tert-Butyl (2-(2-(2-aminoethoxy)ethoxy)ethyl)carbamate (**10**).*

A solution of Boc_2O (4.365 g, 20 mmol, 1 eq) in DCM (40 mL) was added dropwise to a stirring solution of 1,2-Bis(2-aminoethoxy)ethane (14.821 g, 100 mmol, 5 eq) in DCM (100 mL) at 0 °C. The resulting mixture was stirred at rt for 20 h. Subsequently, the solution was washed with water (3 × 100 mL) and brine (100 mL), dried over anhydrous Na_2SO_4 and concentrated in vacuo to give compound **10** as a colourless oil (4.370 g, yield: 88%). 1H NMR (400 MHz, DMSO-*d*$_6$) δ 6.72 (m, 1H), 3.53 - 3.44 (m, 4H), 3.41 - 3.30 (m, 4H), 3.05 (q, 2H), 2.64 (t, 2H), 1.36 (s, 9H).

*6-ethoxy-6-oxohexan-1-aminium chloride (**11**).*

To a suspension of 6-aminohexanoic acid (4.00 g, 30.9 mmol) in EtOH (22 mL), thionyl chloride (3.3 mL, 45.7 mmol) was added. The resulting mixture was refluxed for 3 h. After concentrating the mixture in vacuo, the resulting oily residue was coevaporated three times with diethyl ether to give compound **11** as a white powder. Yield (5.96 g, 100%). ^{1}H NMR (400 MHz, DMSO-*d*$_6$) δ 7.98 (s, 3H), 4.05 (q, J = 7.1 Hz, 2H), 2.79 – 2.60 (m, 2H), 2.29 (t, J = 7.3 Hz, 2H), 1.64 – 1.43 (m, 4H), 1.43 – 1.25 (m, 2H), 1.18 (t, J = 7.1 Hz, 3H).

*6-((tert-butoxycarbonyl)amino)hexanoic acid (**12**).*

6-aminohexanoic acid (3.00 g, 22.9 mmol) was dissolved in water (8 mL) and 1 M NaOH (40 mL), while di-tert-butyl dicarbonate (6.49 g, 29.7 mmol) was dissolved in dioxane (15 mL). The reaction mixtures were combined on ice and stirred at room temperature overnight. Subsequently 1 M NaOH (4 mL) was added to increase the pH to 10. Dioxane was evaporated off *in vacuo* and the residual was washed with ether (20 mL). Water phase was acidified with

1 M HCl to pH 2 and extracted with ethyl acetate (3 × 70 mL). Combined organic layers were dried over Na_2SO_4 and concentrated *in vacuo* to produce compound **12** as a colorless oil (4.55 g, 86%). ^{1}H NMR (400 MHz, DMSO-d_6) δ 11.98 (s, 1H), 6.77 (t, *J* = 5.8 Hz, 1H), 3.57 (s, 1H), 2.88 (q, *J* = 6.9 Hz, 2H), 2.18 (t, *J* = 7.4 Hz, 2H), 1.55 – 1.41 (m, 2H), 1.41 – 1.28 (m, 11H), 1.28 – 1.13 (m, 3H).

2. Supplementary Figures

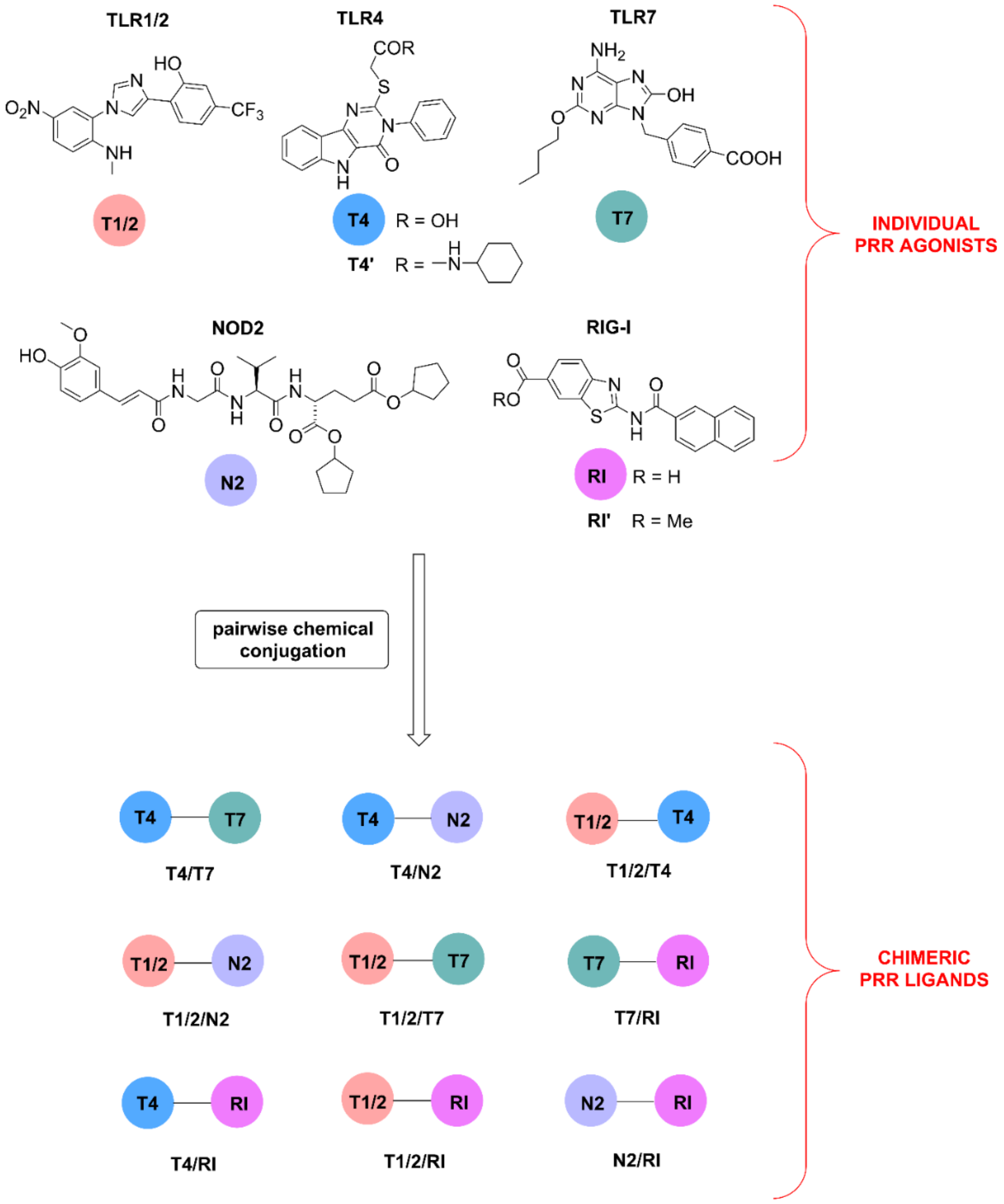

Fig. S1. Chemical structures of the selected PRR agonists and their combinations featured in chimeric PRR ligands.

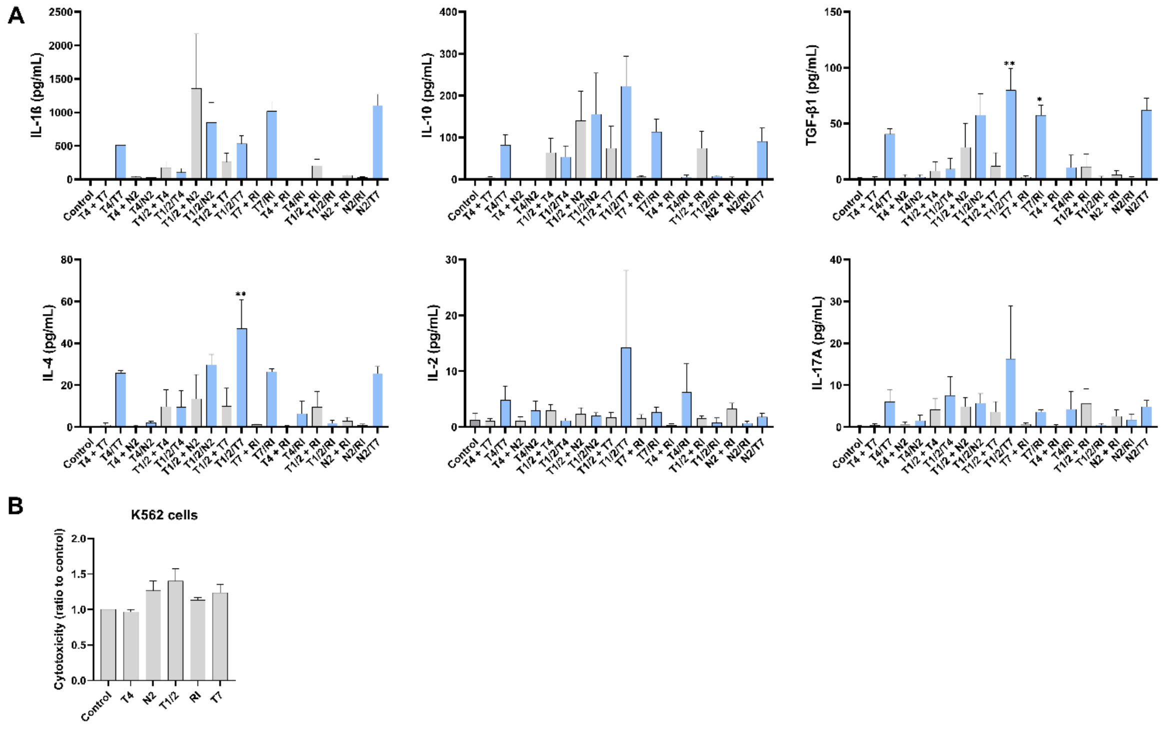

Fig. S2. (A) Cytokine release from human PBMCs after 18 h stimulation with conjugated agonists, unlinked mixtures (both 1 µM), **N2/T7** (1 µM, positive control), or vehicle (0.1% DMSO). Data are mean ± SEM of three independent experiments. One-way ANOVA with Bonferroni's test compared unlinked mixtures to conjugates. *, $p < 0.05$; **, $p < 0.01$ vs. vehicle. (B) PBMC cytotoxicity against K562 cells after 18 h treatment with single agonists (1 µM) or vehicle, followed by 4 h coincubation with K562 cells. Each experiment was conducted in duplicate and repeated three times. Data are relative to the negative control (NT, 0.1% DMSO) and shown as mean ± SEM of three independent experiments. ns, $p > 0.05$ vs. controls (ANOVA with Bonferroni's test).

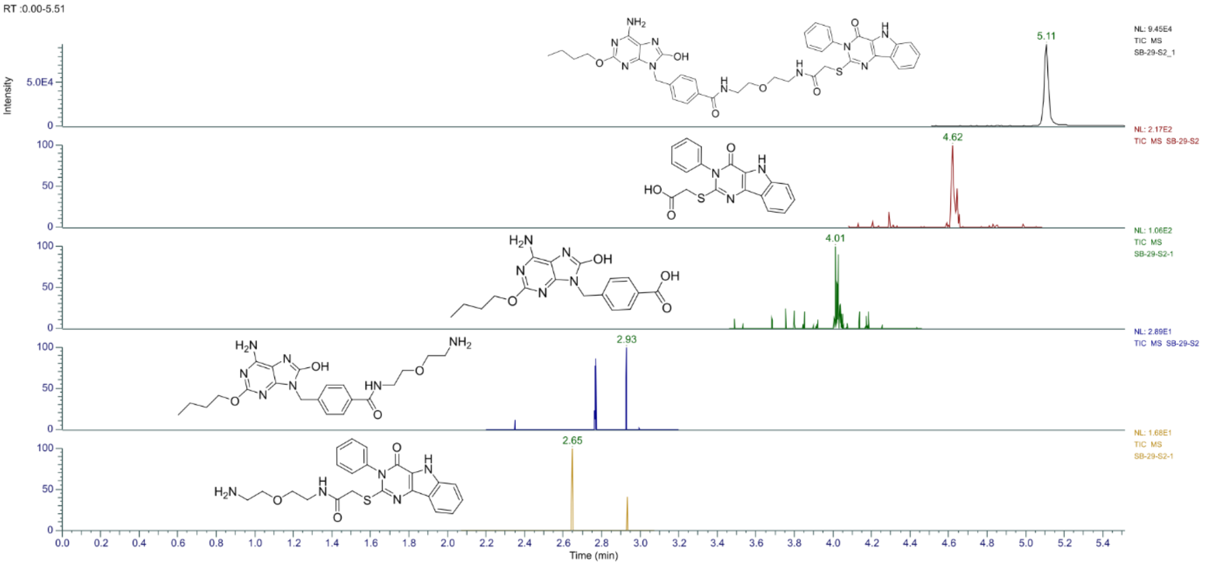

Fig. S3. Extracted-ion chromatograms of compound **T4/T7** and its detected metabolites in PBMC lysates following overnight stimulation with compound **T4/T7**.

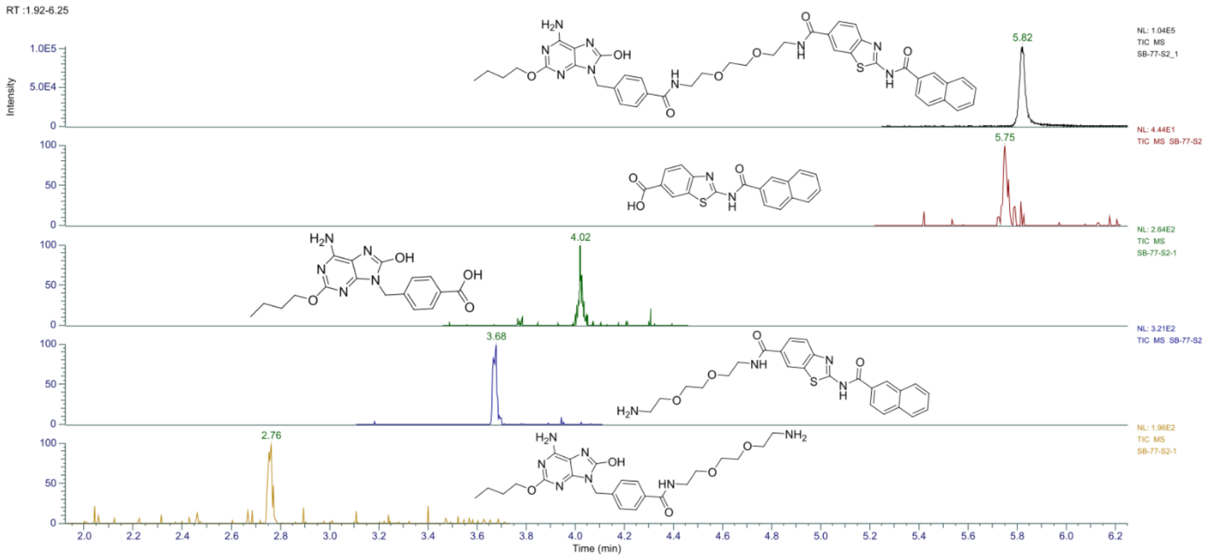

Fig. S4. Extracted-ion chromatograms of compound **T7/RI** and its detected metabolites in PBMC lysates following overnight stimulation with compound **T7/RI**.

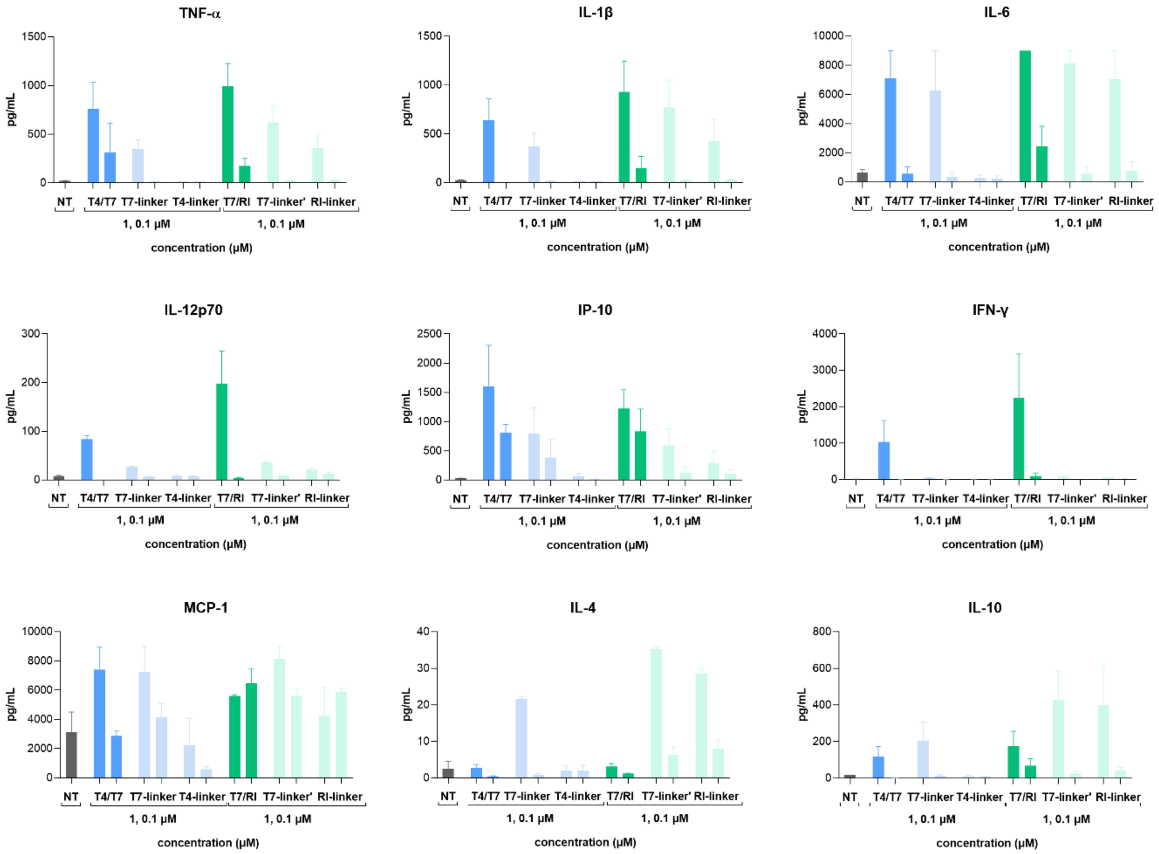

Fig. **S5**. (A) Cytokine release from human PBMCs after 18 h stimulation with conjugated PRR ligand **T4/T7** (dark blue bars) or its metabolites **T7-linker** and **T4-linker** (light blue bars) and **T7/RI** (dark green bars) or its metabolites **T7-linker'** and **RI-linker** (light green bars) (all at 0.1 and 1 µM) or vehicle (grey bars; 0.1% DMSO). Data are mean ± SEM of two independent experiments.

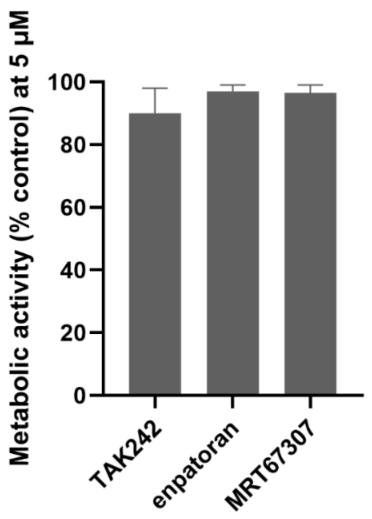

Fig. S6. Commercially available PRR antagonists/inhibitors are not cytotoxic towards PBMCs. Metabolic activities of PBMCs were measured after 18 h treatment with TLR4 antagonist TAK242, TLR7 antagonist enpatoran or inhibitor of RIG-I signaling pathway MRT67307 (all at 5 µM). Data are shown relative to the untreated control (0.1% DMSO). Data are means ±SEM of two independent experiments.

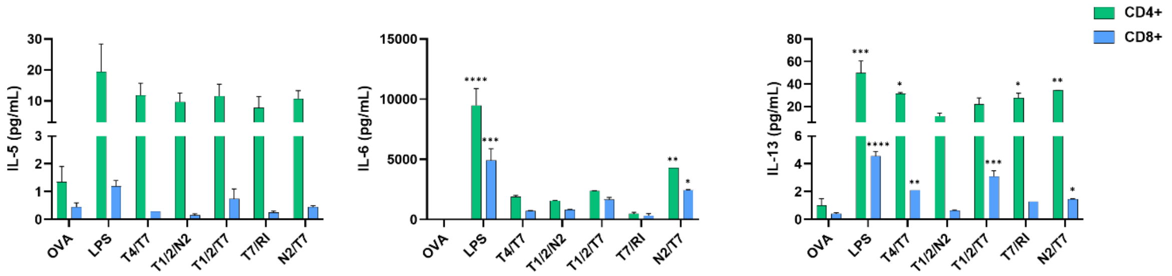

Fig. S7. Cytokine concentrations in BMDC-T-cell coculture supernatants following the 72 h coincubation. BMDCs from C57BL/6 mice were treated for 18 h with compounds (1 µM), LPS (1 µg/mL), or vehicle (0.1% DMSO) in the presence of OVA (50 µg/mL). CFSE-labeled OVA-specific CD4$^+$ or CD8$^+$ T cells (isolated from OT-II or OT-I mouse splenocytes, respectively) were added to the treated and washed BMDCs and cocultured for 72 h. Data are mean ± SEM of duplicates of two independent experiments. *, $p < 0.05$, **, $p <0.01$, ***, $p <0.001$, ****, $p <0.0001$ versus vehicle-treated control. Statistical significance was determined using one-way ANOVA with post-hoc Dunnett's test comparing conjugates versus OVA.

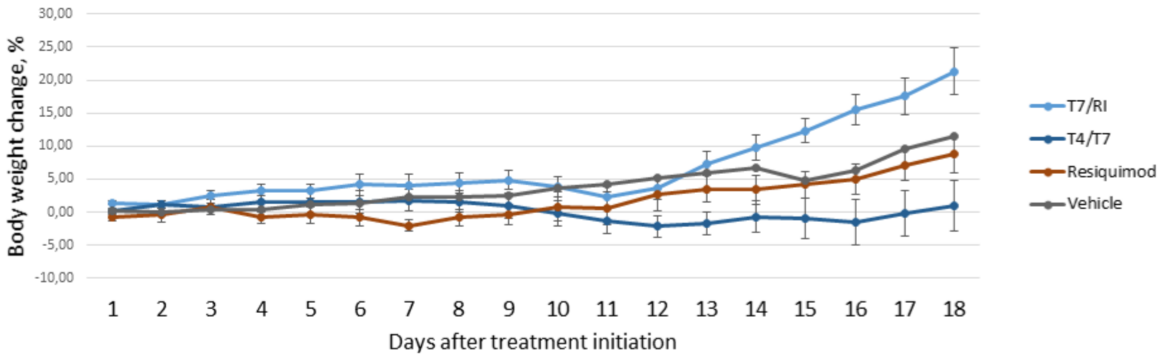

Fig. S8. The dynamics of body weight change values for B16F10 tumors bearing animals of groups treated with vehicle, **T4/T7**, **T7/RI**, and resiquimod. The data are presented as mean ±SEM; p-values are not depicted.

3. Supplementary Schemes

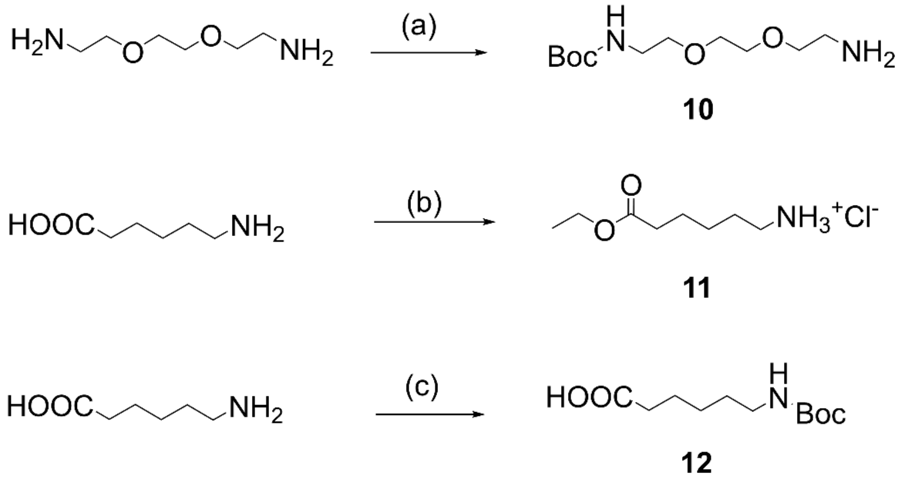

Scheme S1: Synthesis of linkers **10**, **11** and **12**. Reagents and conditions: (a) Boc_2O, DCM, rt.; (b) $SOCl_2$, EtOH, reflux, (c) Boc_2O, NaOH/H_2O, dioxane, rt.

4. Supplementary Tables

Table S1. Cytokine concentrations after treating human PBMCs with conjugates and unlinked mixtures of agonists.

	Average ± SEM [pg/mL]												
	**IL-4**	**IL-2**	**IP-10**	**IL-1β**	**TNF-α**	**MCP-1**	**IL-17A**	**IL-6**	**IL-10**	**IFN-γ**	**IL-12p70**	**IL-8**	**TGF-β1**
**Control**	0.3 ± 0.2	1.2 ± 0.8	21 ± 3.9	0 ± 0	0.8 ± 0.3	35.1 ± 20.8	0.2 ± 0.1	15.2 ± 3.2	0.7 ± 0.3	0.3 ± 0.3	0.3 ± 0.2	45.1 ± 5.8	0.7 ± 0.4
**T1/2**	26 ± 9.4	4.5 ± 3.7	106.4 ± 2	188.9 ± 80.8	1497.8 ± 1058.4	9000 ± 0	9 ± 4.7	6858.2 ± 2141.8	81.9 ± 51.5	52.9 ± 27.5	15.6 ± 7.6	10000 ± 0	32 ± 15.2
**T4**	0.7 ± 0.2	0.6 ± 0.4	43.4 ± 10.6	4.3 ± 2.2	2.3 ± 1	5.9 ± 1.7	0.6 ± 0.3	15.6 ± 6.3	3.5 ± 1.6	1.7 ± 0.9	1 ± 0.4	111.3 ± 37.9	3.6 ± 1.9
**T7**	0.3 ± 0.2	0.9 ± 0.4	79.2 ± 16.2	9 ± 5.6	25.6 ± 18.1	84.4 ± 51.2	1.3 ± 0.5	276.6 ± 97.1	4.6 ± 2.4	3.2 ± 1	1.5 ± 0.4	528.9 ± 272.7	3.8 ± 1
**N2**	1.7 ± 0.8	1 ± 0.6	104.1 ± 2.2	51.9 ± 8	94.6 ± 23.4	338.6 ± 104.6	2.5 ± 0.5	940.8 ± 70.2	1.8 ± 0.2	4.2 ± 0.3	1.5 ± 0	8764.3 ± 3587.7	7.7 ± 1.4
**RI**	0.2 ± 0.2	3.2 ± 1.3	28.3 ± 15.4	1.9 ± 1.9	3.4 ± 2.4	57.5 ± 24.7	0.1 ± 0.1	28.4 ± 5.6	0.7 ± 0.4	0.4 ± 0.4	0.1 ± 0.1	129.5 ± 44.1	0.2 ± 0.2
**T1/2+T4**	9.8 ± 8.2	3 ± 0.9	29.3 ± 24.7	176.2 ± 94.2	405.2 ± 239.3	7109.8 ± 1257.2	4.1 ± 2.6	6169.2 ± 2830.8	63.6 ± 33.9	55.5 ± 40.7	7.2 ± 4.9	6193.1 ± 2703.5	7.7 ± 7.7
**T1/2+T7**	9.9 ± 8.6	1.7 ± 0.9	32.1 ± 28.2	259.8 ± 131.5	651.2 ± 364.9	5705.8 ± 1841.8	3.6 ± 2.5	6203.6 ± 2796.4	74 ± 52.7	31.3 ± 28.8	6.9 ± 5.7	5697.6 ± 2499.5	11.9 ± 11.9
**T1/2+N2**	13.6 ± 11.5	2.4 ± 1.1	37 ± 31.3	1358.7 ± 814.2	1431.1 ± 945.6	7538.3 ± 1461.7	4.9 ± 2.2	8384.8 ± 615.2	140.2 ± 70.7	56.3 ± 29.8	10.4 ± 6.9	7495.1 ± 3018.7	28.6 ± 21.4
**T1/2+RI**	9.5 ± 7.4	1.6 ± 0.4	35.1 ± 30.5	207.5 ± 93.1	524.6 ± 352.6	7712.8 ± 1152.7	5.5 ± 3.5	6633.8 ± 2366.2	74.4 ± 40.3	42.1 ± 25.1	7.1 ± 4.5	6602.9 ± 2653.7	11.3 ± 11.3
**T4+T7**	1.1 ± 0.8	1.1 ± 0.5	25.3 ± 22	11.2 ± 2.7	15 ± 3.9	311.3 ± 168.4	0.4 ± 0.4	205.8 ± 20	5.1 ± 1.3	1.6 ± 1.1	0.3 ± 0.3	481.5 ± 97.5	1.2 ± 1.2
**T4+N2**	0.5 ± 0.3	1.1 ± 0.7	31.2 ± 30.8	39.3 ± 6.6	44.9 ± 21.8	747.9 ± 267.9	0.6 ± 0.6	576 ± 183.9	1.7 ± 0.3	2 ± 2	0.5 ± 0.5	3687 ± 1076.3	2.2 ± 2.2
**T4+RI**	0.4 ± 0.2	0.3 ± 0.3	18.4 ± 17	1 ± 1	2.1 ± 1.7	17.8 ± 14.6	0.3 ± 0.3	27.2 ± 17.2	0.7 ± 0.7	0.6 ± 0.6	0.2 ± 0.2	109.2 ± 62.8	0.3 ± 0.3
**T7+RI**	1.1 ± 0.2	1.6 ± 0.6	45 ± 17.7	14.6 ± 6.1	15.9 ± 5.2	814.5 ± 755.5	0.6 ± 0.3	328.3 ± 129.9	6.4 ± 2.7	2.7 ± 1.2	1 ± 0.5	498.5 ± 234.6	2.2 ± 1.1
**N2+RI**	2.8 ± 1.8	3.2 ± 1	36.9 ± 28.5	64.9 ± 19.4	72.2 ± 35.2	1888.7 ± 1065.2	2.6 ± 1.6	1125.7 ± 366.7	3.9 ± 1.5	7.5 ± 1.5	2.1 ± 1.5	8789 ± 2211	4 ± 4
**T4/T7**	25.8 ± 1.1	4.9 ± 2.5	1595.8 ± 218	513.2 ± 39.4	1320.3 ± 367.1	9000 ± 0	6 ± 2.9	8933.6 ± 36.4	81.9 ± 24.8	1061.7 ± 120.6	82.4 ± 23.4	4476.2 ± 2013	40.6 ± 4.6
**T4/N2**	1.9 ± 0.9	3 ± 1.6	34 ± 31.5	27.6 ± 6.5	38.8 ± 18.2	1435.6 ± 880.1	1.5 ± 1.3	484.7 ± 175.9	2.3 ± 0.5	3.1 ± 2.3	0.8 ± 0.8	5473.7 ± 2849.9	2.2 ± 2.2
**T1/2/T4**	9.4 ± 8	1.2 ± 0.4	29.6 ± 27.1	105.8 ± 56.2	190.5 ± 141.2	6117.8 ± 2570.4	7.5 ± 4.4	6059.6 ± 2940.4	53.5 ± 26.5	12.2 ± 7.4	6.1 ± 4.2	5039.3 ± 1861.2	9.4 ± 9.4
**T1/2/N2**	29.7 ± 5	2 ± 0.6	106.8 ± 9.2	848.9 ± 298.5	4768.4 ± 2311.5	7885.4 ± 608.1	5.7 ± 2.3	9000 ± 0	156.1 ± 97.5	198.1 ± 67.4	28.8 ± 4.2	8939.6 ± 762.1	57.6 ± 19.2

**T1/2/T7**	47 ± 13.9	14.2 ± 13.8	379.8 ± 142.4	533.9 ± 118.8	3045 ± 2094.4	8212.1 ± 366.8	16.3 ± 12.7	9000 ± 0	222.3 ± 71.6	579.4 ± 398.1	58.9 ± 13.8	8048.5 ± 1951.5	79.9 ± 19.2
**T7/RI**	26.5 ± 1.4	2.7 ± 0.9	1974.8 ± 284.1	1019.1 ± 147	2037.7 ± 483	8910.8 ± 89.2	3.6 ± 0.4	9000 ± 0	113.4 ± 31.1	3335.1 ± 582.8	95.5 ± 18	5132.5 ± 2443.8	57.3 ± 9.2
**T4/RI**	6.3 ± 6	6.3 ± 5	35.4 ± 33.8	9.1 ± 6.1	6.9 ± 6.4	32 ± 17.4	4.3 ± 4.3	26.8 ± 12.5	5.6 ± 4.9	5.4 ± 5.4	4.5 ± 4.5	91.7 ± 27	10.8 ± 10.8
**T1/2/RI**	1.8 ± 1.3	0.9 ± 0.8	34.6 ± 33.7	4.5 ± 3.6	6.5 ± 2.2	2062.7 ± 1461.7	0.4 ± 0.4	279.1 ± 84.6	7.8 ± 1.8	1.3 ± 1.2	0.6 ± 0.6	1230.7 ± 504.1	1.4 ± 1.4
**N2/RI**	0.7 ± 0.6	0.7 ± 0.4	32.4 ± 30.7	29 ± 11.2	42.2 ± 24.2	279.8 ± 164.7	1.7 ± 1.5	434.6 ± 257.1	1.3 ± 0.7	1.4 ± 1.4	0.6 ± 0.6	1438.9 ± 549.6	1.3 ± 1.3
**N2/T7**	25.7 ± 3.2	1.8 ± 0.6	1765.2 ± 259.5	1106.8 ± 166.3	2391.5 ± 594.1	8814.4 ± 110.7	4.8 ± 1.6	9000 ± 0	90.3 ± 32.1	2149.4 ± 515.1	146.6 ± 55.5	8568.5 ± 1431.5	62 ± 10.9

Table S2A. Contributions of protein analytes to the two principal component axes.

Loadings		
**Variable**	**PC1**	**PC2**
**IL-4**	0.967	-0.125
**IL-2**	0.631	-0.294
**IP-10**	0.617	0.753
**IL-1β**	0.794	0.130
**TNF-α**	0.858	-0.151
**MCP-1**	0.894	-0.088
**IL-17A**	0.811	-0.442
**IL-6**	0.930	-0.076
**IL-10**	0.941	-0.233
**IFN-γ**	0.613	0.744
**IL-12p70**	0.841	0.494
**IL-8**	0.648	-0.418
**TGF-β1**	0.966	-0.006

Table S2B. Contributions of samples to the two principal component axes.

Samples	PC1	PC2
**NT**	-2.494	0.485
**T1/2**	2.607	-1.774
**T4**	-2.418	0.514
**T7**	-2.262	0.437
**N2**	-1.433	-0.364
**RI**	-2.352	0.345
**T1/2 + T4**	0.405	-0.805
**T1/2 + T7**	0.361	-0.652
**T1/2 + N2**	2.684	-0.963
**T1/2 + RI**	0.670	-0.910
**T4 + T7**	-2.356	0.419
**T4 + N2**	-2.059	0.142
**T4 + RI**	-2.554	0.532
**T7 + RI**	-2.211	0.383
**N2 + RI**	-1.151	-0.635
**T4/T7**	4.202	2.275
**T4/N2**	-1.657	-0.244
**T1/2/T4**	0.193	-0.861
**T1/2/N2**	4.534	-1.310
**T1/2/T7**	7.212	-2.276
**T7/RI**	5.849	4.864
**T4/RI**	-1.417	-0.223
**T1/2/RI**	-2.139	0.340
**N2/RI**	-2.216	0.280

Table S3. Percentage of dead K562 cells, dead PBMCs and ratio of dead K562 cells compared to untreated control.

	Average ± SEM		
	% dead K562	dead K562, ratio to NT	% dead PBMCs
**Control**	24.2 ± 2.1	1.0 ± 0.0	2.23 ± 0.32
**T4**	22.0 ± 2.8	0.94 ± 0.03	1.79 ± 0.74
**N2**	25.8 ± 1.8	1.25 ± 0.13	1.05 ± 0.23
**T1/2**	29.3 ± 5.3	1.38 ± 0.15	1.36 ± 0.56
**RI**	30.4 ± 3.3	1.11 ± 0.03	1.27 ± 0.30
**T7**	25.9 ± 4.2	1.23 ± 0.11	1.76 ± 0.21
**T4 + T7**	30.6 ± 1.0	1.14 ± 0.12	1.35 ± 0.19
**T4/T7**	77.3 ± 2.5	3.74 ± 0.23	1.18 ± 0.31
**T4 + N2**	30.9 ± 3.4	1.0 ± 0.0	1.28 ± 0.33
**T4/N2**	28.3 ± 2.9	1.01 ± 0.16	1.27 ± 0.17
**T1/2 + T4**	43.4 ± 4.9	1.40 ± 0.31	8.71 ± 4.10
**T1/2/T4**	26.3 ± 3.5	1.26 ± 0.13	2.02 ± 0.35
**T1/2 + N2**	33.4 ± 4.3	1.60 ± 0.14	2.92 ± 1.86
**T1/2/N2**	35.7 ± 3.2	2.11 ± 0.18	2.85 ± 1.58
**T1/2 + T7**	36.6 ± 5.7	1.73 ± 0.14	1.42 ± 0.53
**T1/2/T7**	64.8 ± 7.0	3.12 ± 0.30	1.36 ± 0.41
**T7 + RI**	31.2 ± 1.2	1.19 ± 0.10	1.04 ± 0.13
**T7/RI**	79.1 ± 1.7	3.84 ± 0.30	1.45 ± 0.34
**T4 + RI**	36.8 ± 2.5	1.17 ± 0.05	1.11 ± 0.02
**T4/RI**	25.2 ± 2.0	0.88 ± 0.08	2.27 ± 0.76
**T1/2 + RI**	45.3 ± 0.1	1.45 ± 0.17	9.30 ± 4.30
**T1/2/RI**	38.0 ± 0.8	1.35 ± 0.17	1.55 ± 0.20
**N2 + RI**	37.4 ± 0.2	1.19 ± 0.13	1.46 ± 0.31
**N2/RI**	32.4 ± 2.2	1.13 ± 0.06	1.20 ± 0.12
**N2/T7**	77.6 ± 3.5	3.75 ± 0.25	1.29 ± 0.28
**IL-2**	75.5 ± 3.9	3.18 ± 0.40	1.67 ± 0.31

Table S4A. Extracellular and intracellular concentrations of **T4/T7**, **T7/RI** and single agonists after treatment of PBMCs (all at 1 µM).

**Compounds**	**Extracellular concentration (ng/mL)**	**Intracellular concentration (ng/mL)**
**T4**	252.77	0.95
**T7**	331.18	0.90
**RI**	302.32	1.86
**T4/T7**	667.22	52.44
**T7/RI**	209.44	126.21

Table S4B. Intracellular concentrations of **T4/T7** and detected degradation products/metabolites after treatment of PBMCs with 1 µM **T4/T7**.

**Compounds**	**Structures**	**Intracellular concentration (ng/mL)**
**T4/T7**	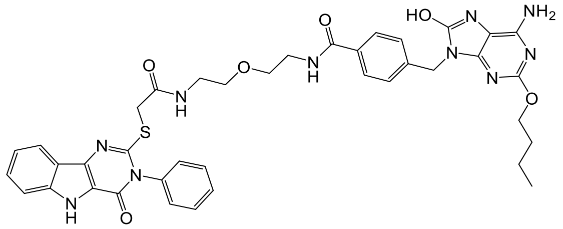	52.44
**T4**	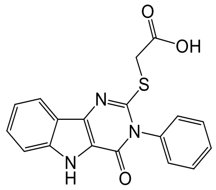	0.59
**T7**	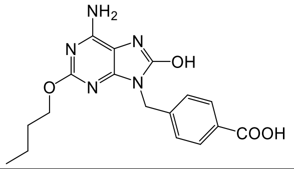	0.34
**T4-linker**	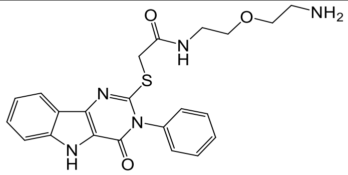	0.80
**T7-linker**	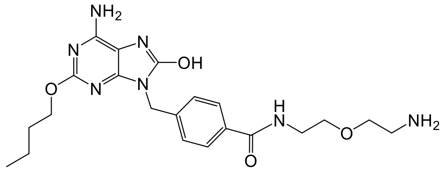	0.33

Table S4C. Intracellular concentrations of **T7/RI** and detected degradation products/metabolites after treatment of PBMCs with 1 μM **T7/RI**.

**Compounds**	**Structures**	**Intracellular concentration (ng/mL)**
**T7/RI**	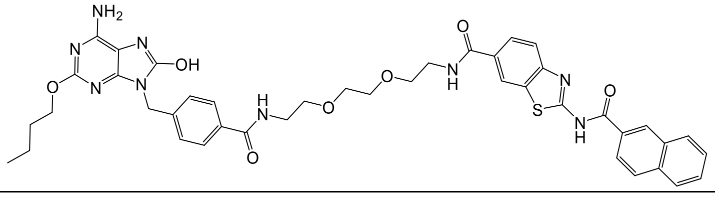	126.21
**T7**	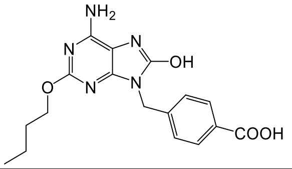	0.36
**RI**	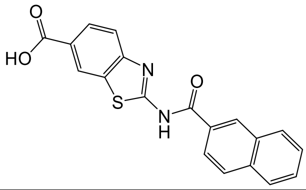	0.14
**T7-linker'**	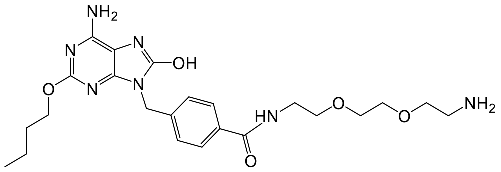	0.61
**RI-linker**	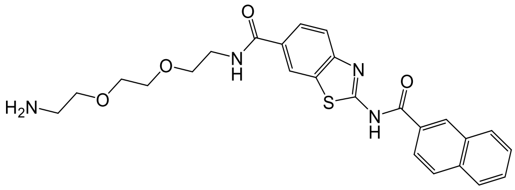	nd

Table S5A: Cytokine concentrations after treatment of human PBMCs with conjugates **T4/T7** and **T7/RI** and the corresponding antagonists.

	Average ± SEM [pg/mL]							
	**IP-10**	**IL-1b**	**TNF-α**	**MCP-1**	**IL-6**	**IL-10**	**IFN-γ**	**IL-8**
**NT**	50.23 ± 24.06	1.35 ± 0	0.22 ± 0.11	547.01 ± 104.2	71.02 ± 8.21	0.65 ± 0.18	0.23 ± 0.06	347.69 ± 51.88
**T4/T7**	700.01 ± 149.69	1.35 ± 0	311.34 ± 303.24	2867.75 ± 287.03	133.49 ± 39.62	0.84 ± 0.2	3.45 ± 1.37	420.57 ± 66.55
**T4/T7+TAK242**	11.88 ± 4.97	1.35 ± 0	0.14 ± 0.08	112.51 ± 37.81	12.69 ± 7.52	0.08 ± 0.01	0.06 ± 0.02	20.63 ± 2.99
**T4/T7+M5049**	34.43 ± 20.19	2.39 ± 1.04	0.23 ± 0.07	337.07 ± 10.33	49.8 ± 15.07	0.69 ± 0.16	0.26 ± 0.16	296.4 ± 38.6
**T4/T7+TAK242+M5049**	1.06 ± 0.35	1.35 ± 0	0.03 ± 0.01	8.8 ± 1.99	1.53 ± 0.8	0.07 ± 0	0.04 ± 0.01	5.63 ± 1.52
**T7/RI**	938.2 ± 166.38	150.49 ± 77	171.72 ± 52.23	6829.18 ± 897.04	2440.01 ± 854.67	66.8 ± 22.58	95.49 ± 51.03	1043.24 ± 169.12
**T7/RI+M5049**	31.65 ± 5.79	1.79 ± 0.44	0.47 ± 0.11	489.25 ± 43.03	74.02 ± 19.06	1.33 ± 0.87	0.28 ± 0.08	248.59 ± 79.24
**T7/RI+MRT67307**	5.56 ± 0.61	1.35 ± 0	0.04 ± 0.01	105.38 ± 8.71	39.66 ± 6.46	0.18 ± 0.05	0.08 ± 0.02	168.77 ± 37.88
**T7/RI+M5049+MRT67307**	7.04 ± 1.67	1.35 ± 0	0.04 ± 0.01	66.4 ± 9.53	66.94 ± 14.34	0.25 ± 0.1	0.15 ± 0.06	313.17 ± 78.07

Table S5B: Percentage of dead K562 cells, dead PBMCs and ratio of dead K562 cells compared to untreated control after pretreatment of human PBMCs with conjugates **T4/T7** and **T7/RI** and the corresponding antagonists.

	Average ±SEM		
	**% dead K562**	**dead K562, ratio to NT**	**% dead PBMCs**
**NT**	56.4 ± 7.5	1.0 ± 0.0	1.6 ± 0.4
**IL-2**	88.9 ± 0.8	1.6 ± 0.1	1.6 ± 0.1
**T4/T7**	84.2 ± 1.3	1.5 ± 0.1	1.4 ± 0.2
**T4/T7+TAK242**	37.8 ± 4.9	0.7 ± 0.1	3.2 ± 0.5
**T4/T7+M5049**	47.4 ± 1.3	0.8 ± 0.0	5.1 ± 0.5
**T4/T7+TAK242+M5049**	16.7 ± 1.2	0.3 ± 0.0	6.5 ± 0.5
**T7/RI**	80.7 ± 1.8	1.5 ± 0.1	1.3 ± 0.1
**T7/RI+M5049**	7.7 ± 0.3	0.1 ± 0.0	2.9 ± 0.7
**T7/RI+MRT67307**	45.4 ± 2.9	0.8 ± 0.0	6.0 ± 0.6
**T7/RI+M5049+MRT67307**	5.0 ± 2.1	0.1 ± 0.0	6.1 ± 0.3

Table S6A: Proliferation indexes of $CD4^+$ and $CD8^+$ T cells.

	Average ± SEM	
	**$CD4^+$ (OT-II) proliferation index**	**$CD8^+$ (OT-I) proliferation index**
**w/o OVA**	1.1 ± 0.0	1.1 ± 0.0
**OVA**	1.4 ± 0.1	2.0 ± 0.1
**LPS**	1.4 ± 0.0	2.4 ± 0.1
**T4/T7**	2.1 ± 0.1	3.2 ± 0.1
**T1/2/N2**	2.3 ± 0.0	3.6 ± 0.0
**T1/2/T7**	2.2 ± 0.0	3.5 ± 0.1
**T7/RI**	2.1 ± 0.0	3.5 ± 0.1
**N2/T7**	2.0 ± 0.1	3.2 ± 0.1

Table S6B: Cytokine concentrations in BMDC-T-cell coculture.

		**Average ± SEM**											
		**IL-2**	**IL-4**	**IL-5**	**IL-6**	**IL-9**	**IL-10**	**IL-13**	**IL-17A**	**IL-17F**	**IL-22**	**IFN-γ**	**TNF-α**
**CD4+**	**OVA only**	41.8 ± 3.9	0.7 ± 0.4	1.3 ± 0.5	11 ± 1.1	2.7 ± 1.5	0 ± 0	1 ± 0.5	6 ± 3.5	0.9 ± 0	2.2 ± 0.4	33.9 ± 1.7	13.1 ± 3.5
	**LPS**	873.5 ± 102.7	3.7 ± 0.2	19.3 ± 9.1	9490.7 ± 1406.1	520.2 ± 47.2	25.7 ± 4.4	49.7 ± 10.7	1655.4 ± 217.3	693.4 ± 68.5	5923.9 ± 784	6377 ± 1114.1	218.3 ± 26.5
	**T4/T7**	790.8 ± 11.1	5.8 ± 1.1	11.8 ± 3.9	1910.9 ± 97.6	349.9 ± 34.2	5.8 ± 0	31.5 ± 0.9	1213.6 ± 221.3	436.9 ± 62.2	3485.4 ± 174.5	2265.7 ± 151.3	176.5 ± 6.2
	**T1/2/N2**	1108 ± 83.6	2.4 ± 0.3	9.7 ± 2.8	1560 ± 39.9	368.5 ± 32.5	3.3 ± 0.3	11.8 ± 2.5	431.7 ± 4.7	199.2 ± 6.4	2446.9 ± 47	1095.1 ± 44	137 ± 7.8
	**T1/2/T7**	1052 ± 127.5	3.8 ± 1.3	11.5 ± 3.9	2399.8 ± 4	354.9 ± 11.8	10 ± 0.3	22 ± 5.8	1010.3 ± 53.9	415.2 ± 11	3557.6 ± 421.9	2268.4 ± 55.6	145.2 ± 12.2
	**T7/RI**	476.7 ± 89.1	39.1 ± 4.8	7.7 ± 3.7	491.9 ± 117.6	334.8 ± 9.2	2.3 ± 2.3	27.9 ± 4.1	925.1 ± 177.3	439.5 ± 105.5	1874.4 ± 229.3	1041.4 ± 132.8	140.7 ± 24.1
	**N2/T7**	722.9 ± 84	5 ± 1.4	10.7 ± 2.6	4304.1 ± 0.6	328.1 ± 4.5	14.4 ± 0.2	34.4 ± 0.2	1336.2 ± 2.4	623.2 ± 63.1	4058.6 ± 428.9	2673.2 ± 559.8	165.6 ± 9.9
**CD8+**	**OVA only**	123.2 ± 30.7	0.2 ± 0.2	0.5 ± 0.2	2.2 ± 0.2	0.8 ± 0.2	0 ± 0	0.4 ± 0.1	0.3 ± 0	0.7 ± 0	0.5 ± 0.2	15.9 ± 8	5.7 ± 0.8
	**LPS**	36.6 ± 6.6	0.8 ± 0.1	1.2 ± 0.2	4925.2 ± 965.8	33.8 ± 4.9	8.8 ± 0.5	4.5 ± 0.4	403.4 ± 43.8	157.9 ± 23.3	826.5 ± 19.1	3326.4 ± 263.9	172.1 ± 11.8
	**T4/T7**	117.8 ± 29.4	0 ± 0	0.3 ± 0	732.8 ± 22.5	5.5 ± 0.1	0 ± 0	2.1 ± 0	148.4 ± 23	80.5 ± 5.7	339.6 ± 21.4	434.1 ± 31.3	121.8 ± 5.3
	**T1/2/N2**	81.7 ± 6.6	0.1 ± 0.1	0.2 ± 0	815.2 ± 34.1	6.1 ± 0.5	0 ± 0	0.7 ± 0.1	33.3 ± 0.4	12.6 ± 5.6	86.4 ± 6.6	276 ± 1.3	152.9 ± 4
	**T1/2/T7**	74.5 ± 29.6	0.1 ± 0.1	0.8 ± 0.3	1664 ± 185.9	13.9 ± 0.9	4.4 ± 2.7	3.1 ± 0.4	240.7 ± 8.1	107.2 ± 2	546.6 ± 40.5	748.6 ± 126.4	143.2 ± 2.3
	**T7/RI**	141.5 ± 5.9	0.1 ± 0	0.3 ± 0.1	328.8 ± 162.6	2 ± 1.1	0 ± 0	1.3 ± 0	80.3 ± 7.3	32.4 ± 1.9	161.3 ± 45.5	345.8 ± 89.7	98.5 ± 10.1
	**N2/T7**	60.9 ± 3.3	0.1 ± 0	0.5 ± 0	2441.4 ± 58.4	11.7 ± 0.1	3.7 ± 0.5	1.4 ± 0.1	236.7 ± 15.6	100 ± 10.3	433.2 ± 7.3	917.4 ± 75.1	131.9 ± 7.9

Table S7A. Concentration of OVA-specific IgG, IgG1 and IgG2a antibody responses (screening).

	Average ± SEM [AU/mL]		
	**Total IgG**	**IgG1**	**IgG2a**
**OVA**	10.6 ± 0.6	161.6 ± 49.2	6.6 ± 1.2
**OVA + ALUM**	113.6 ± 49.2	922.8 ± 273.4	8.0 ± 1.2
**OVA + T4/T7**	1449.2 ± 231.4	10506.8 ± 2228.4	1106.0 ± 306
**OVA + T1/2/N2**	237.8 ± 18.9	2084.4 ± 409.2	49.0 ± 29.1
**OVA + T1/2/T7**	339.4 ± 52.6	2886.0 ± 554.6	12.8 ± 4.0
**OVA + T7/RI**	1289.4 ± 154.7	8488.6 ± 2916.2	1435.2 ± 420.1

Table S7B. Concentration of OVA-specific IgG, IgG1 and IgG2a antibody responses (benchmark study).

	Average ± SEM [AU/mL]		
	**Total IgG**	**IgG1**	**IgG2a**
**OVA**	92.8 ± 55.8	922.9 ± 556.1	3830 ± 2212
**OVA + MDP**	4589 ± 1441	53,468 ± 12,954	10,928 ± 3725
**OVA + imiquimod**	4310 ± 1908	3317 ± 1078	16,809 ± 7806
**OVA + T4/T7**	101,736 ± 21,377	212,855 ± 35,547	10,466,355 ± 2,792,084

Table S8A. Tumor volumes of B16F10 tumors.

	Average ± SEM tumor volume (mm^3)									
**Days after treatment initiation (DPT)**	**0**	**2**	**4**	**6**	**8**	**10**	**12**	**14**	**16**	**18**
**Vehicle**	102.4 ± 11.3	133.5 ± 14.3	191.2 ± 22.6	341 ± 54.0	499.8 ± 59.2	701.9 ± 79.7	1086.7 ± 103.3	1454.5 ± 146.2	1886.1 ± 155.5	2287.6 ± 154.6
**T4/T7**	100.3 ± 9.1	130.5 ± 17.6	169.1 ± 24.6	295 ± 55.8	385.9 ± 78.1	501.4 ± 100.9	684 ± 149.8	910.6 ± 219.3	1079.7 ± 279.5	1132.4 ± 199.6
**T7/RI**	105.3 ± 15.4	129.2 ± 17.6	162.3 ± 21.4	282.3 ± 48.1	397.1 ± 70.8	575.8 ± 96.4	893.4 ± 138.2	1013.4 ± 141.3	1548 ± 215.9	2006.3 ± 295.8
**Resiquimod**	102.1 ± 9.6	136.1 ± 12.2	177.6 ± 16.8	232.3 ± 27.6	290.2 ± 25.9	351.2 ± 36.4	505.5 ± 59.2	723.5 ± 76.5	1038.9 ± 104.1	1236.2 ± 156.1

Table S8B. Calculated TGI and T/C values.

	**TGI, %**			**T/C, %**		
**Groups**	**maximal**	**DPT 16**	**DPT 18**	**lowest**	**DPT 16**	**DPT 18**
**T4/T7**	58.95 (DPT 18)	50.25	58.95	49.5 (DPT 18)	57.25	49.50
**T7/RI**	32.1 (DPT 14)	25.96	30.62	69.7 (DPT 16)	69.67	87.70
**Resiquimod**	56.3 (DPT 18)	48.99	56.27	54.0 (DPT 18)	55.08	54.04

Table S8C. Survival of mice.

**Groups**	**vehicle**	**T4/T7**	**T7/RI**	**resiquimod**
**Median survival [days]**	18	22	20	24

Table S9. Individual clinical signs in B16F10 tumor bearing C57BL/6 mice during the study.

Group	mouse #	Days after treatment initiation																		
		0	1	2	3	4	5	6	7	8	9	10	11	12	13	14	15	16	17	18
Vehicle	11	/	/	/	/	/	/	/	P	H,P,D	H,P,D	H,P,D	H,P,D	H,P,D	H,P,D	H,P,D,Eh	H,P,D,Eh	H,P,D,Eh	H,P,D,Eh	H,P,D,Eh
	32	/	/	/	/	/	/	/	P	P	P	P, D	H,P,D	H,P,D	H,P,D,Eh	H,P,D,Eh	H,P,D,Eh	H,P,D,Eh	H,P,D,Eh	H,P,D,Eh
	38	/	/	/	/	/	/	/	/	P	P	P	P,D	P,D	P,D	H,P,D,Eh	H,P,D,Eh	H,P,D,Eh	H,P,D,Eh	H,P,D,Eh
	44	/	/	/	/	/	/	/	P	H,P,D	H,P,D	H,P,D	H,P,D	H,P,D	H,P,D	H,P,D	H,P,D	H,P,D	H,P,D,Eh	H,P,D,Eh
	82	/	/	/	/	/	/	/	/	/	/	P,D	P,D	H,P,D,Eh	H,P,D,Eh	H,P,D,Eh	H,P,D,Eh	H,P,D,Eh	H,P,D,Eh	H,P,D,Eh
	86	/	/	/	/	/	/	/	/	P, D	P,D	P,D	P,D,Eh	H,P,D,Eh	H,P,D,Eh	H,P,D,Eh	H,P,D,Eh	H,P,D,Eh	H,P,D,Eh	H,P,D,Eh
	92	/	/	/	/	/	/	/	/	H,P,D	H,P,D	H,P,D	H,P,D	H,P,D,Eh	H,P,D,Eh	H,P,D,Eh	H,P,D,Eh	H,P,D,Eh	H,P,D,Eh	H,P,D,Eh
	57	/	/	/	/	/	/	/	/	/	P,D	P,D	P,D	P,D	P,D,Eh	P,D,Eh	P,D,Eh	P,D,Eh	H,P,D,Eh	H,P,D,Eh

Group	mouse #	Days after treatment initiation																		
		0	1	2	3	4	5	6	7	8	9	10	11	12	13	14	15	16	17	18
T7/RI	53	/	/	/	/	/	/	D	P,D	P,D	P,D	P,D	P,D	H,P,D	H,P,D	H,P,D,Eh	H,P,D,Eh	H,P,D,Eh	H,P,D,Eh	H,P,D,Eh
	9	/	/	/	/	/	/	P,D	P,D	P,D	H,P,D	H,P,D	H,P,D,Eh	H,P,D,Eh	H,P,D,Eh	H,P,D,Eh	H,P,D,Eh	H,P,D,Eh	H,P,D,Eh	H,P,D,Eh
	12	/	/	/	/	/	/	D	P,D	P,D	H,P,D	H,P,D	H,P,D,Eh	H,P,D,Eh	Died	N/A	N/A	N/A	N/A	N/A
	43	/	/	/	/	/	/	/	P	P,D	P,D	H,P,D	H,P,D,Eh	H,P,D,Eh	H,P,D,Eh	H,P,D,Eh	H,P,D,Eh	H,P,D,Eh	H,P,D,Eh	H,P,D,Eh
	46	/	/	/	/	/	/	/	P	P	P,D	P,D	P,D	P,D	P,D	P,D	P,D	H,P,D	H,P,D	H,P,D,Eh
	83	/	/	/	/	/	/	/	P	P	P, D	P, D	P, D	H,P,D	H,P,D	H,P,D	H,P,D	H,P,D	H,P,D,Eh	H,P,D,Eh
	87	/	/	/	/	/	/	/	P	P	P, D	P, D	P, D	P,D,Eh	H,P,D,Eh	H,P,D,Eh	H,P,D,Eh	H,P,D,Eh	H,P,D,Eh	H,P,D,Eh
	94	/	/	/	/	/	/	/	P	P	P,D	P,D	P,D	P,D	H,P,D	H,P,D	H,P,D	H,P,D	H,P,D	H,P,D

Group	mouse #	Days after treatment initiation																		
		0	1	2	3	4	5	6	7	8	9	10	11	12	13	14	15	16	17	18
T4/T7	3	/	/	/	/	/	/	/	P	P	P	P,D	P,D	P,D	P,D	P,D,Eh	P,D,Eh	H,P,D,Eh	H,P,D,Eh	H,P,D,Eh
	34	/	/	/	/	/	/	/	/	P	P	P,D	H,P,D	H,P,D,Eh	H,P,D,Eh	H,P,D,Eh	H,P,D,Eh	H,P,D,Eh	H,P,D,Eh	H,P,D,Eh
	25	/	/	/	/	/	/	/	P	P	P	H,P,D	H,P,D	H,P,D,Eh	H,P,D,Eh	H,P,D,Eh	H,P,D,Eh	H,P,D,Eh	**Eu**	N/A
	63	/	/	/	/	/	/	/	/	P	P	P,D	P,D	P,D	H,P,D	H,P,D,Eh	H,P,D,Eh	H,P,D,Eh	H,P,D,Eh	H,P,D,Eh
	90	/	/	/	/	/	/	/	P	P	H,P,D	H,P,D	H,P,D	H,P,D	H,P,D,Eh	H,P,D,Eh	H,P,D,Eh	H,P,D,Eh	H,P,D,Eh	H,P,D,Eh
	49	/	/	/	/	/	/	P, D	P, D	P,D	H,P,D	H,P,D	H,P,D	H,P,D	H,P,D	H,P,D,Eh	H,P,D,Eh	H,P,D,Eh	H,P,D,Eh	H,P,D,Eh
	54	/	/	/	/	/	/	/	/	/	P	P,D	P,D	P,D	P,D	P,D,Eh	P,D,Eh	P,D,Eh	H,P,D,Eh	H,P,D,Eh
	8	/	/	/	/	/	/	/	/	P	P,D	P,D	P,D	P,D	P,D	P,D,Eh	H,P,D,Eh	H,P,D,Eh	H,P,D,Eh	H,P,D,Eh

Group	mouse #	Days after treatment initiation																		
		0	1	2	3	4	5	6	7	8	9	10	11	12	13	14	15	16	17	18
Resiquimod	69	/	/	/	/	/	/	/	P	P	P	P	P	P,D	P,D	H,P,D	H,P,D	H,P,D	H,P,D ,Eh	H,P,D ,Eh
	4	/	/	/	/	/	/	P	P	P	P	P	P	P	P,D	H,P,D	H,P,D	H,P,D	H,P,D	H,P,D
	95	/	/	/	/	/	/	/	P	P	P	P	P	P,D	P,D	H,P,D	H,P,D	H,P,D	H,P,D ,Eh	H,P,D ,Eh
	19	/	/	/	/	/	/	P, D	P, D	P,D	P,D	P,D	P,D	P,D	P,D	H,P,D ,Eh	H,P,D ,Eh	H,P,D, Eh	H,P,D ,Eh	H,P,D ,Eh
	107	/	/	/	/	/	/	/	/	P,D	P,D	P,D	P,D	P,D	P,D	H,P,D	H,P,D ,Ep	H,P,D, Ep	H,P,D ,Ep	H,P,D ,Ep
	51	/	/	/	/	/	/	/	/	D	P,D	P,D	P,D	P,D	P,D	H,P,D	H,P,D ,Ep	H,P,D, Ep	H,P,D ,Ep	H,P,D ,Ep
	103	/	/	/	/	/	/	/	P	P	P,D	P,D	P,D	P,D	P,D	H,P,D	H,P,D	H,P,D, Ep	H,P,D ,Ep	H,P,D ,Ep
	101	/	/	/	/	/	/	/	P	P	P,D	P,D	P,D	P,D	P,D	H,P,D	H,P,D ,Ep	H,P,D, Ep	H,P,D ,Ep	H,P,D ,Ep

H – Hunched posture, P – Piloerection, D – Decreased activity (hypokinesia), Da – Diarrhea, Ds – Dyspnoa, Eh – Half-closed eyes, En – Eyes do not open on touch, Ep – Pale/clouded eyes, W – Wounds, Eu – Euthanized.

Table S10. The optimized instrument conditions for analyzed compounds.

Cpd	Retention time (min)	Precursor ion (m/z)	Product ion (m/z)	Ion source voltage (V)	Collision energy (V)	Vaporizer temperature (°C)	Sheath gas (AU)	Auxiliary gas (AU)	Sweep gas (AU)
**T7/RI**	5.75	409.66	155.05	3500	30	200	50	10	1
**T7**	3.96	358.15	184.08	4000	20	200	55	10	1
**T7-linker'**	2.73	244.63	160.08, 284.08	3500	20	160	40	8	1
**RI**	5.72	349.06	155.05	4000	20	180	50	10	1
**RI-linker**	3.61	479.17	331.05, 374.09	3500	25	160	50	10	1
**T4/T7**	5.01	389.15	334.06	4000	15	200	50	10	1
**T4**	4.58	352.07	171.05, 199.05, 260.08	3500	25	150	50	10	1
**T7-linker**	2.57	222.62	284.08, 327.12	3500	15	160	55	12	1
**T4-linker**	2.7	438.16	334.06	3500	20	180	50	8	1

5. HPLC chromatograms/traces of final compounds

Compound **T4/T7**

Chromatogram and Results

Injection Details

Injection Name:	T4/T7	*Run Time (min):*	12,50
Vial Number:	GB3	*Injection Volume:*	
Injection Type:		*Channel:*	UV_VIS_2
Calibration Level:		*Wavelength:*	254
Instrument Method:	Method2021	*Bandwidth:*	16
Processing Method:	Quantitative	*Dilution Factor:*	1,0000
Injection Date/Time:		*Sample Weight:*	1,0000

Chromatogram

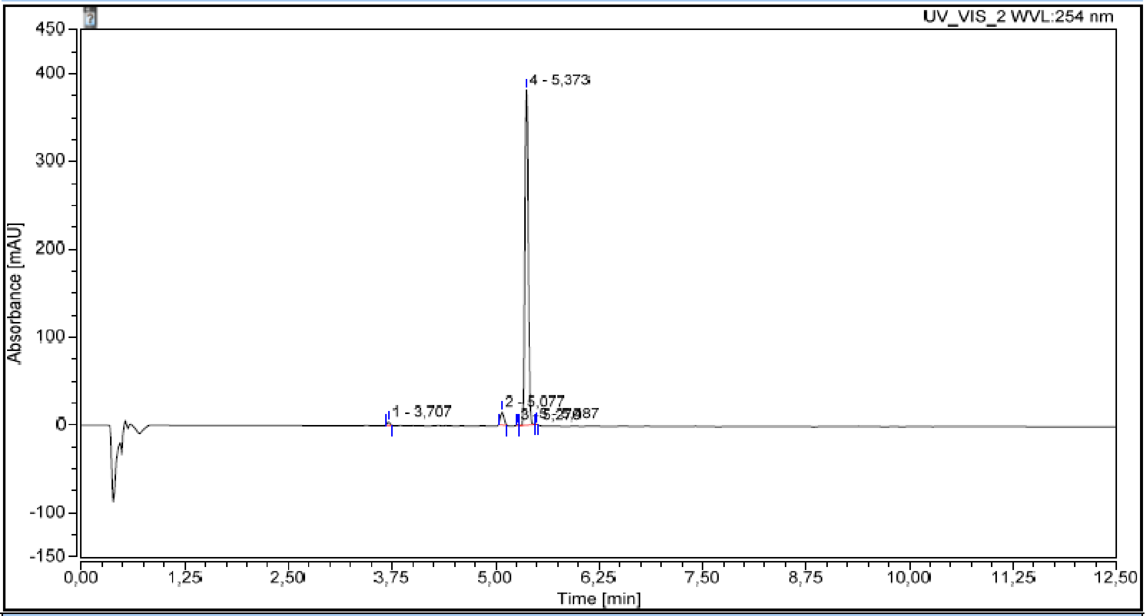

Integration Results

No.	Peak Name	Retention Time min	Area mAU*min	Height mAU	Relative Area %	Relative Height %	Amount n.a.
1		3,707	0,162	4,452	0,87	1,11	n.a.
2		5,077	0,710	15,064	3,83	3,74	n.a.
3		5,270	0,025	0,857	0,13	0,21	n.a.
4		5,373	17,606	381,538	95,03	94,72	n.a.
5		5,487	0,024	0,904	0,13	0,22	n.a.
**Total:**			**18,527**	**402,815**	**100,00**	**100,00**	

Compound **T4/N2**

Chromatogram and Results

Injection Details

Injection Name:	T4/N2	*Run Time (min):*	12,50
Vial Number:	BB6	*Injection Volume:*	
Injection Type:		*Channel:*	UV_VIS_2
Calibration Level:		*Wavelength:*	254
Instrument Method:	Method2021	*Bandwidth:*	16
Processing Method:	Quantitative	*Dilution Factor:*	1,0000
Injection Date/Time:		*Sample Weight:*	1,0000

Chromatogram

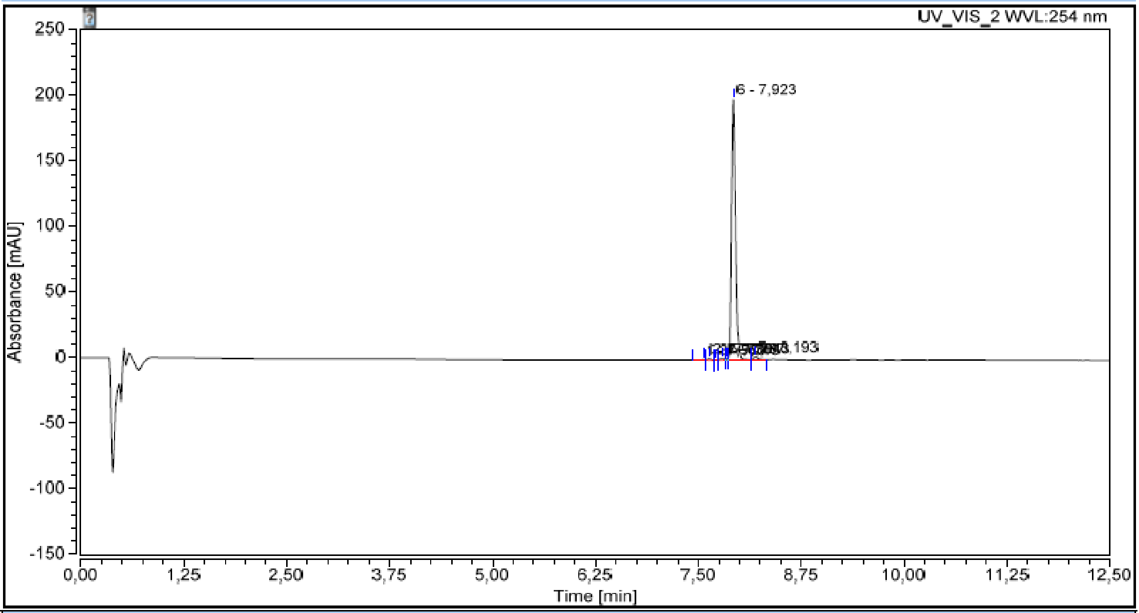

Integration Results

No.	Peak Name	Retention Time min	Area mAU*min	Height mAU	Relative Area %	Relative Height %	Amount n.a.
1		7,560	0,013	0,242	0,12	0,12	n.a.
2		7,630	0,060	1,149	0,52	0,56	n.a.
3		7,713	0,008	0,190	0,07	0,09	n.a.
4		7,787	0,074	1,102	0,65	0,54	n.a.
5		7,843	0,026	0,991	0,23	0,48	n.a.
6		7,923	11,184	198,616	97,17	97,17	n.a.
7		8,193	0,144	2,121	1,25	1,04	n.a.
**Total:**			**11,510**	**204,410**	**100,00**	**100,00**	

Compound **T1/2/T4**

Chromatogram and Results

Injection Details

Injection Name:	T1/2/T4	*Run Time (min):*	12,00
Vial Number:	BB4	*Injection Volume:*	
Injection Type:		*Channel:*	UV_VIS_2
Calibration Level:		*Wavelength:*	254
Instrument Method:	Method2021_12	*Bandwidth:*	16
Processing Method:	Quantitative	*Dilution Factor:*	1,0000
Injection Date/Time:		*Sample Weight:*	1,0000

Chromatogram

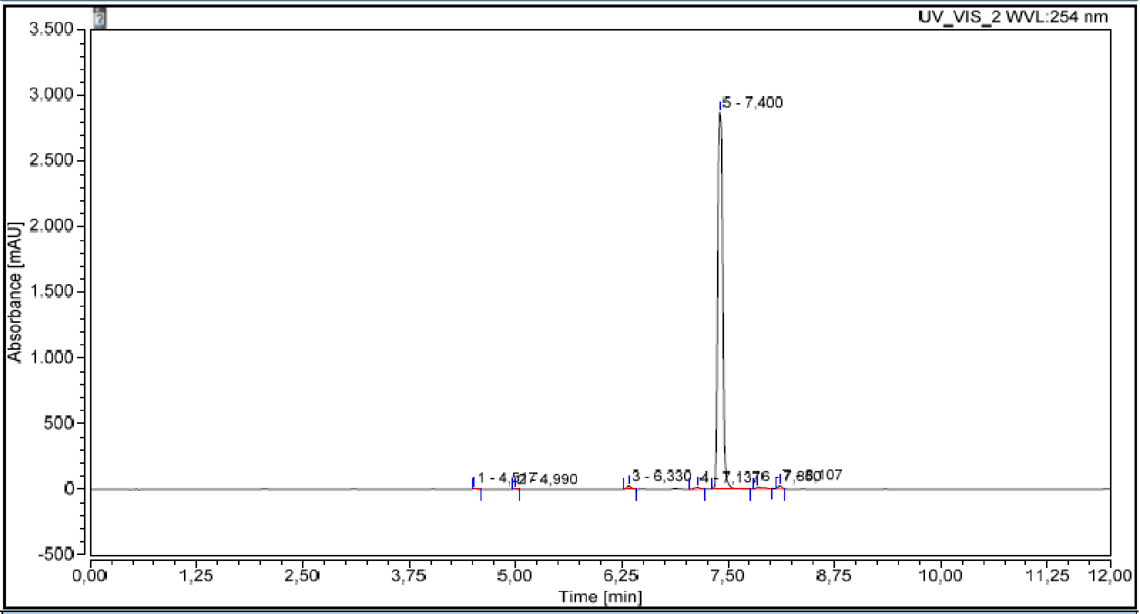

Integration Results

No.	Peak Name	Retention Time min	Area mAU*min	Height mAU	Relative Area %	Relative Height %	Amount n.a.
1		4,517	0,310	7,433	0,15	0,25	n.a.
2		4,990	0,110	2,537	0,05	0,09	n.a.
3		6,330	1,573	28,336	0,76	0,95	n.a.
4		7,137	1,030	14,844	0,50	0,50	n.a.
5		7,400	201,222	2873,035	97,04	96,69	n.a.
6		7,850	1,832	18,820	0,88	0,63	n.a.
7		8,107	1,292	26,451	0,62	0,89	n.a.
**Total:**			**207,369**	**2971,456**	**100,00**	**100,00**	

Compound **T1/2/N2**

Chromatogram and Results

Injection Details

Injection Name:	T1/2/N2	*Run Time (min):*	12,50
Vial Number:	BB5	*Injection Volume:*	
Injection Type:		*Channel:*	UV_VIS_2
Calibration Level:		*Wavelength:*	254
Instrument Method:	Method2021	*Bandwidth:*	16
Processing Method:	Quantitative	*Dilution Factor:*	1,0000
Injection Date/Time:		*Sample Weight:*	1,0000

Chromatogram

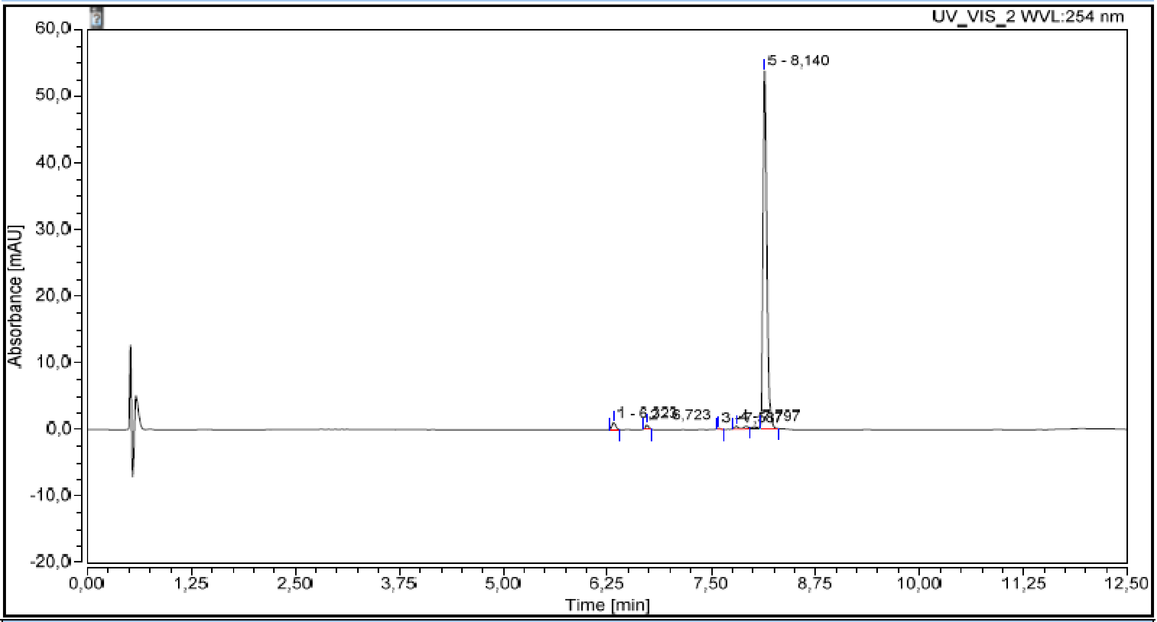

Integration Results

No.	Peak Name	Retention Time min	Area mAU*min	Height mAU	Relative Area %	Relative Height %	Amount n.a.
1		6,323	0,057	1,065	1,86	1,90	n.a.
2		6,723	0,031	0,683	1,02	1,22	n.a.
3		7,587	0,005	0,141	0,18	0,25	n.a.
4		7,797	0,056	0,495	1,83	0,88	n.a.
5		8,140	2,919	53,793	95,11	95,76	n.a.
Total:			3,069	56,177	100,00	100,00	

Compound **T1/2/T7**

Chromatogram and Results

Injection Details

Injection Name:	T1/2/T7	*Run Time (min):*	12,00
Vial Number:	BB2	*Injection Volume:*	
Injection Type:		*Channel:*	UV_VIS_2
Calibration Level:		*Wavelength:*	254
Instrument Method:	Method2021_12	*Bandwidth:*	16
Processing Method:	Quantitative(1)	*Dilution Factor:*	1,0000
Injection Date/Time:		*Sample Weight:*	1,0000

Chromatogram

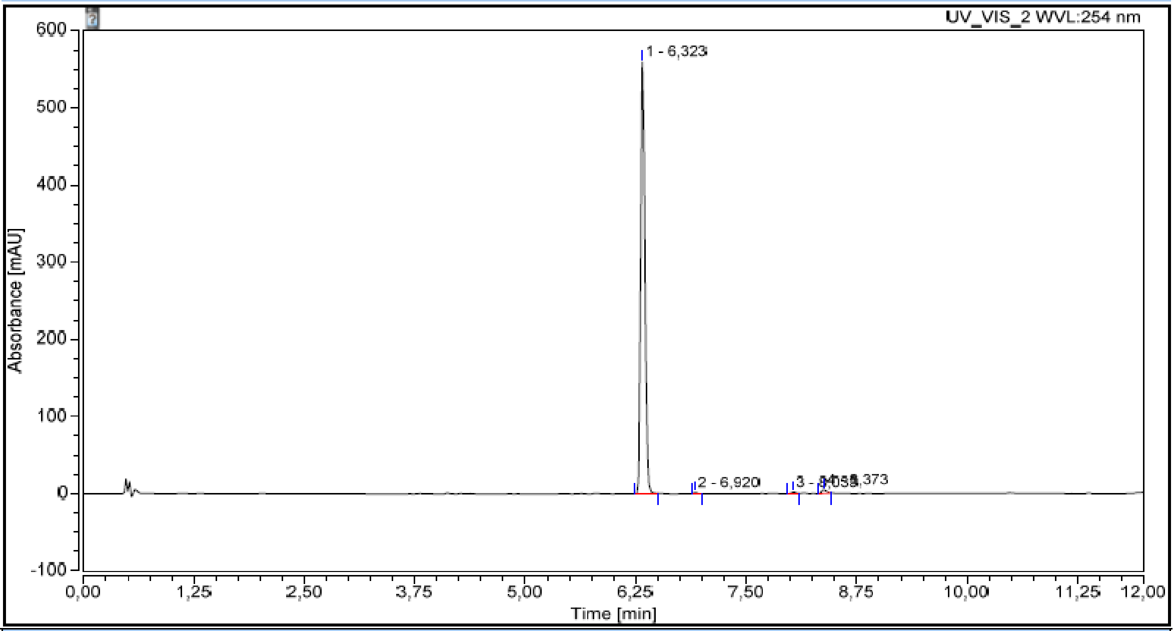

Integration Results

No.	Peak Name	Retention Time min	Area mAU*min	Height mAU	Relative Area %	Relative Height %	Amount n.a.
1		6,323	30,897	560,071	98,45	98,60	n.a.
2		6,920	0,081	1,600	0,26	0,28	n.a.
3		8,033	0,106	1,784	0,34	0,31	n.a.
4		8,373	0,298	4,579	0,95	0,81	n.a.
**Total:**			**31,382**	**568,034**	**100,00**	**100,00**	

Compound **T7/RI**

Chromatogram and Results

Injection Details

Injection Name:	T7/RI	*Run Time (min):*	8,00
Vial Number:	RD2	*Injection Volume:*	5,00
Injection Type:	Unknown	*Channel:*	UV_VIS_1
Calibration Level:		*Wavelength:*	210
Instrument Method:	Method2021	*Bandwidth:*	8
Processing Method:	Quantitative	*Dilution Factor:*	1,0000
Injection Date/Time:		*Sample Weight:*	1,0000

Chromatogram

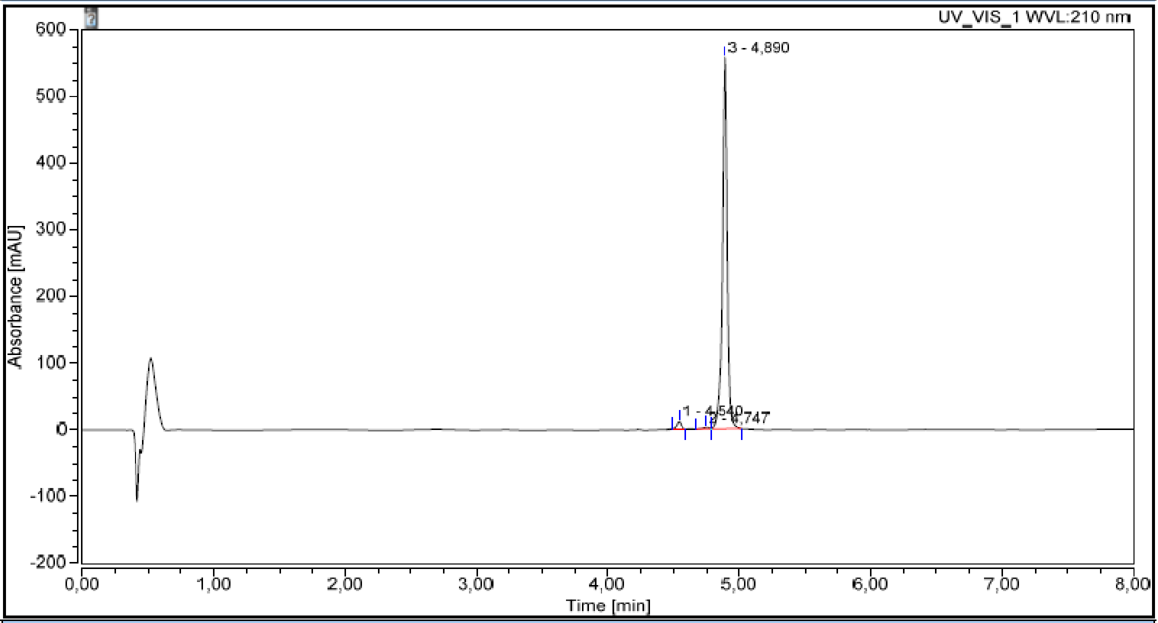

Integration Results

No.	Peak Name	Retention Time<br>min	Area<br>mAU*min	Height<br>mAU	Relative Area<br>%	Relative Height<br>%	Amount<br>n.a.
1		4,540	0,490	12,510	1,97	2,19	n.a.
2		4,747	0,146	2,320	0,59	0,41	n.a.
3		4,890	24,201	557,444	97,44	97,41	n.a.
Total:			24,837	572,274	100,00	100,00	

Compound **T4/RI**

Chromatogram and Results

Injection Details

Injection Name:	T4/RI	*Run Time (min):*	12,50
Vial Number:	GB4	*Injection Volume:*	
Injection Type:		*Channel:*	UV_VIS_2
Calibration Level:		*Wavelength:*	254
Instrument Method:	Method2021	*Bandwidth:*	16
Processing Method:	Quantitative	*Dilution Factor:*	1,0000
Injection Date/Time:		*Sample Weight:*	1,0000

Chromatogram

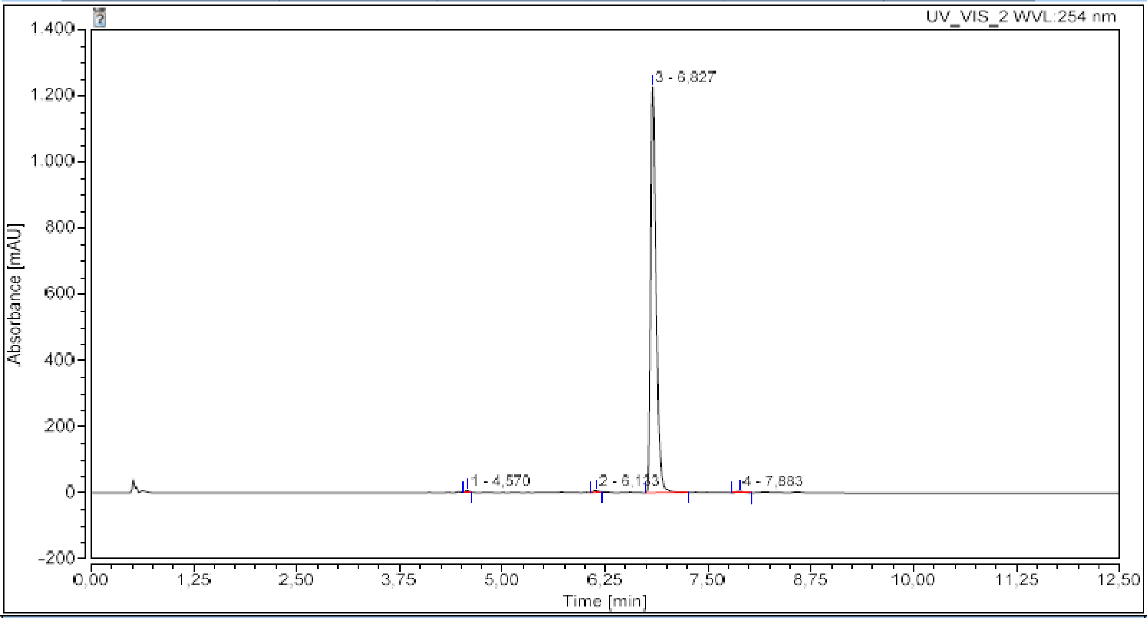

Integration Results

No.	Peak Name	Retention Time<br>min	Area<br>mAU*min	Height<br>mAU	Relative Area<br>%	Relative Height<br>%	Amount<br>n.a.
1		4,570	0,267	5,407	0,28	0,43	n.a.
2		6,133	0,324	5,158	0,34	0,41	n.a.
3		6,827	94,781	1228,734	99,01	98,78	n.a.
4		7,883	0,360	4,642	0,38	0,37	n.a.
**Total:**			**95,732**	**1243,941**	**100,00**	**100,00**	

Compound **T1/2/RI**

Chromatogram and Results

Injection Details

Injection Name:	T1/2/RI	*Run Time (min):*	12,50
Vial Number:	GB6	*Injection Volume:*	
Injection Type:		*Channel:*	UV_VIS_2
Calibration Level:		*Wavelength:*	254
Instrument Method:	Method2021	*Bandwidth:*	16
Processing Method:	Quantitative	*Dilution Factor:*	1,0000
Injection Date/Time:		*Sample Weight:*	1,0000

Chromatogram

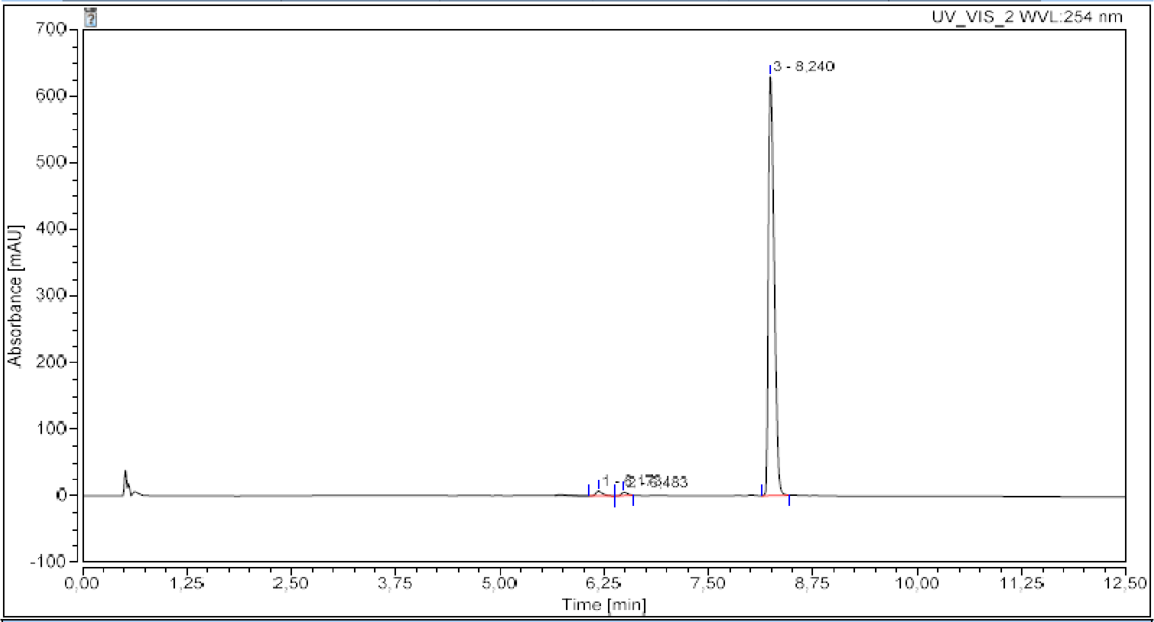

Integration Results

No.	Peak Name	Retention Time min	Area mAU*min	Height mAU	Relative Area %	Relative Height %	Amount n.a.
1		6,173	0,709	7,762	1,34	1,21	n.a.
2		6,483	0,445	5,486	0,84	0,85	n.a.
3		8,240	51,653	630,243	97,82	97,94	n.a.
**Total:**			**52,807**	**643,491**	**100,00**	**100,00**	

Compound **N2/RI**

Chromatogram and Results

Injection Details

Injection Name:	N2/RI	*Run Time (min):*	12,50
Vial Number:	BB3	*Injection Volume:*	
Injection Type:		*Channel:*	UV_VIS_2
Calibration Level:		*Wavelength:*	254
Instrument Method:	Method2021	*Bandwidth:*	16
Processing Method:	Quantitative	*Dilution Factor:*	1,0000
Injection Date/Time:		*Sample Weight:*	1,0000

Chromatogram

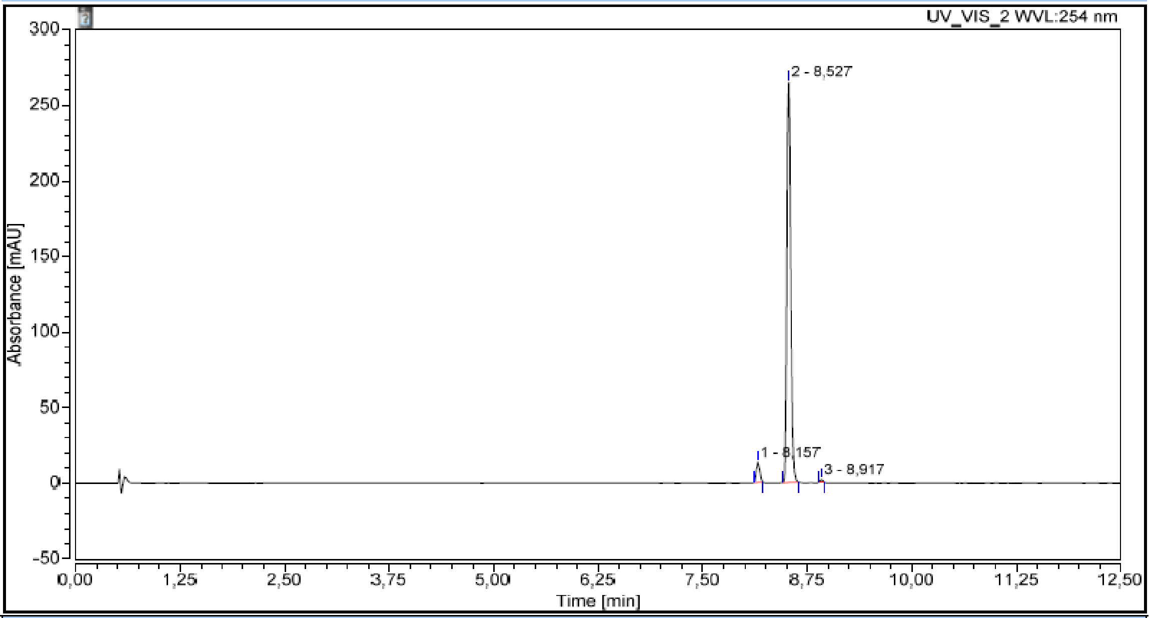

Integration Results

No.	Peak Name	Retention Time min	Area mAU*min	Height mAU	Relative Area %	Relative Height %	Amount n.a.
1		8,157	0,680	13,456	4,52	4,80	n.a.
2		8,527	14,315	264,958	95,04	94,59	n.a.
3		8,917	0,066	1,692	0,44	0,60	n.a.
Total:			15,062	280,105	100,00	100,00	

6. HRMS spectra of final compounds

Compound **T4/T7**

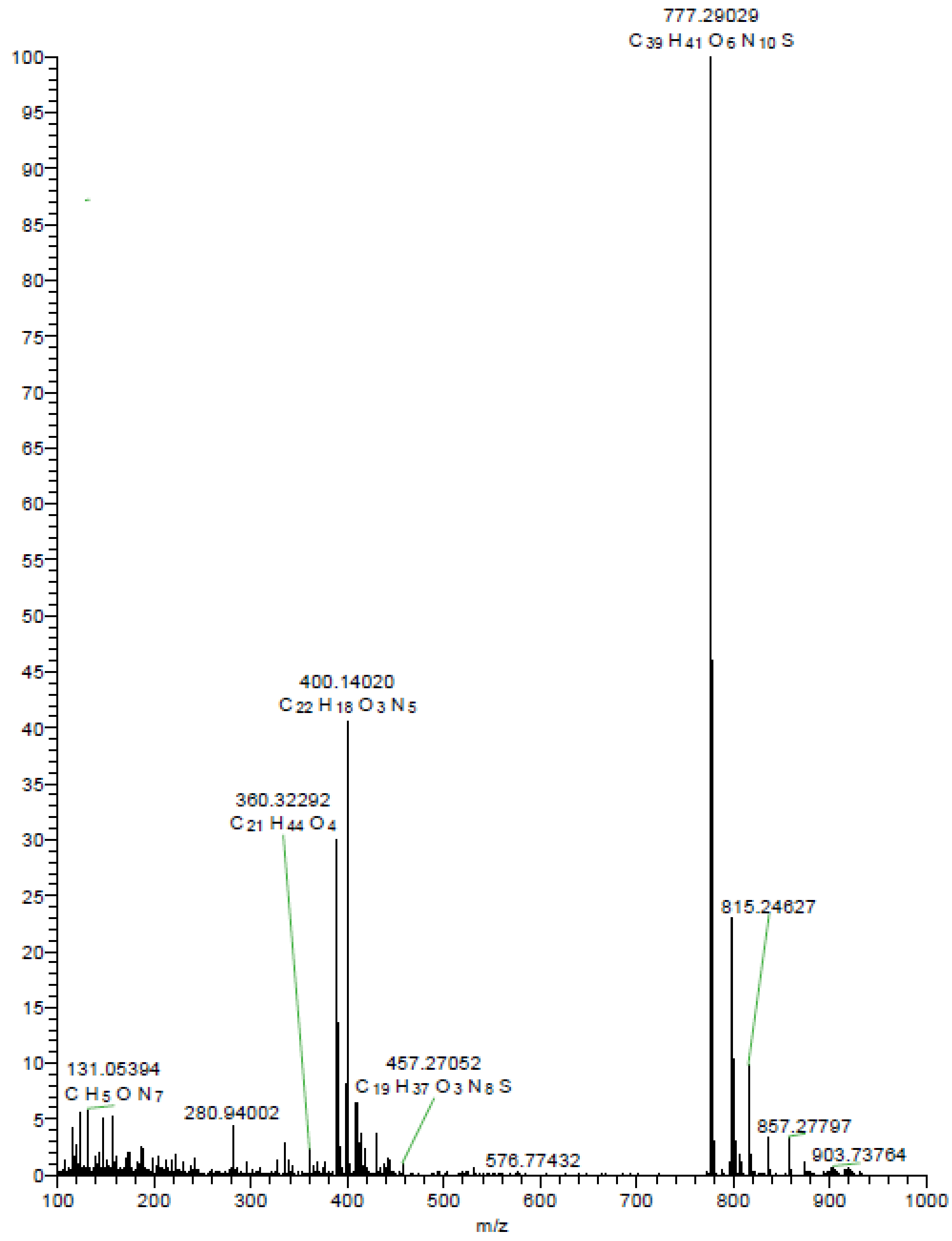

Elemental composition search on mass 777.29029

m/z= 772.29029-782.29029

m/z	Theo. Mass	Delta (ppm)	RDB equiv.	Composition
777.29029	777.29258	-2.94	24.5	$C_{39}H_{41}O_6N_{10}S$

Compound **T4/N2**

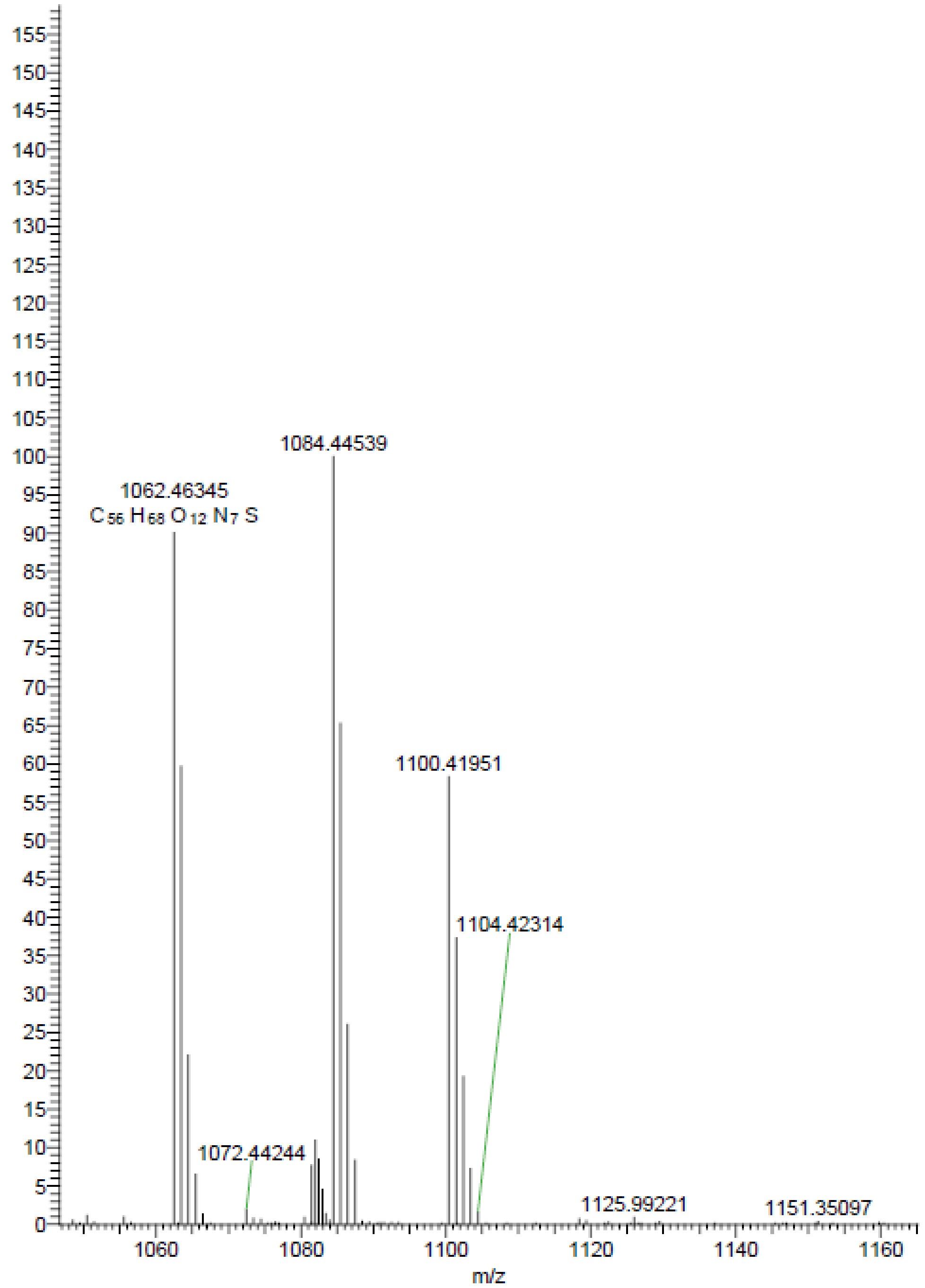

Elemental composition search on mass 1062.46345

m/z= 1057.46345-1067.46345

m/z	Theo. Mass	Delta (ppm)	RDB equiv.	Composition
1062.46345	1062.46412	-0.63	26.5	$C_{56}H_{68}O_{12}N_7S$

Compound **T1/2/T4**

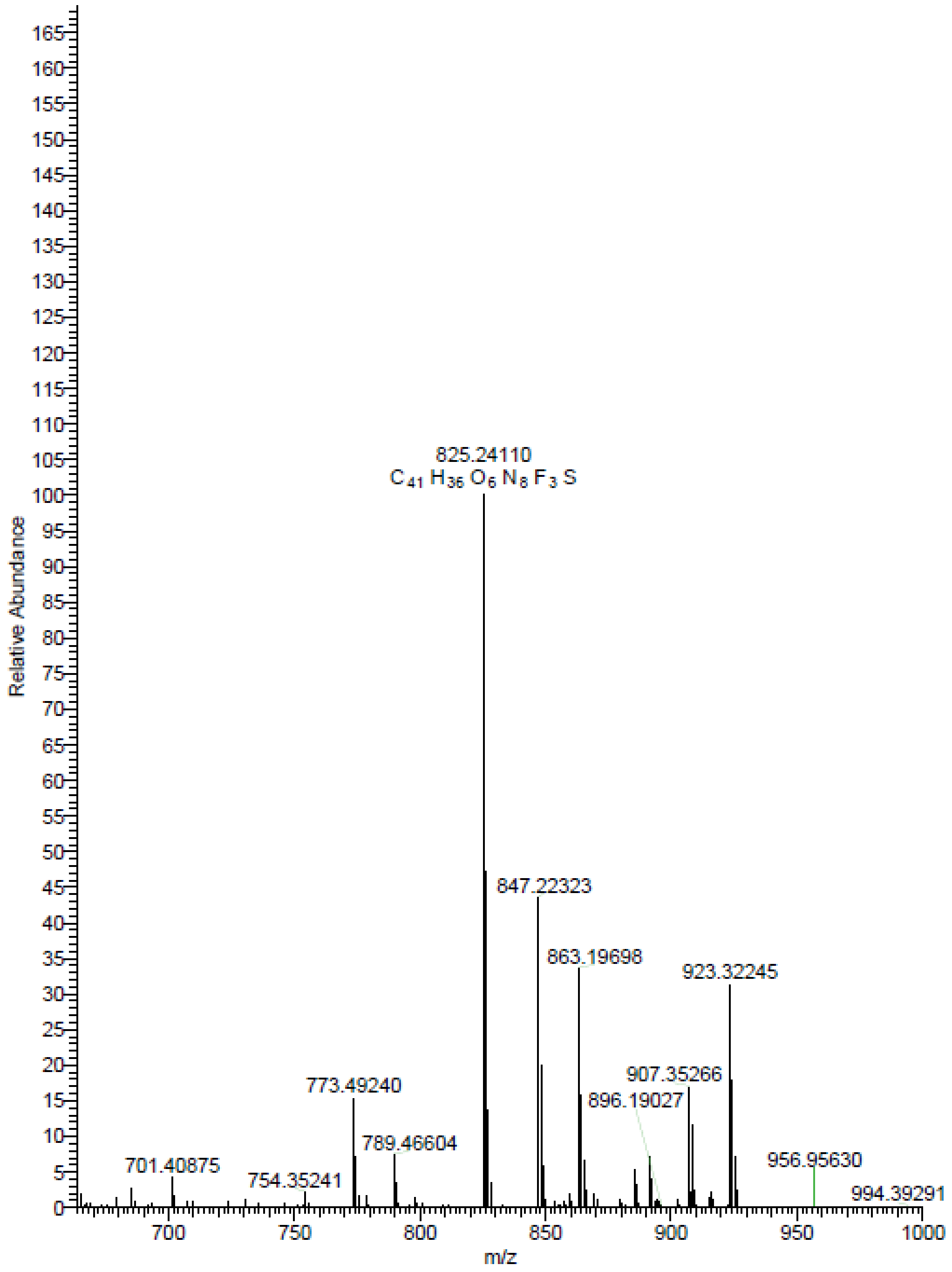

Elemental composition search on mass 825.24110

m/z= 820.24110-830.24110

m/z	Theo. Mass	Delta (ppm)	RDB equiv.	Composition
825.24110	825.24251	-1.71	26.5	$C_{41}H_{36}O_6N_8F_3S$

Compound **T1/2/N2**

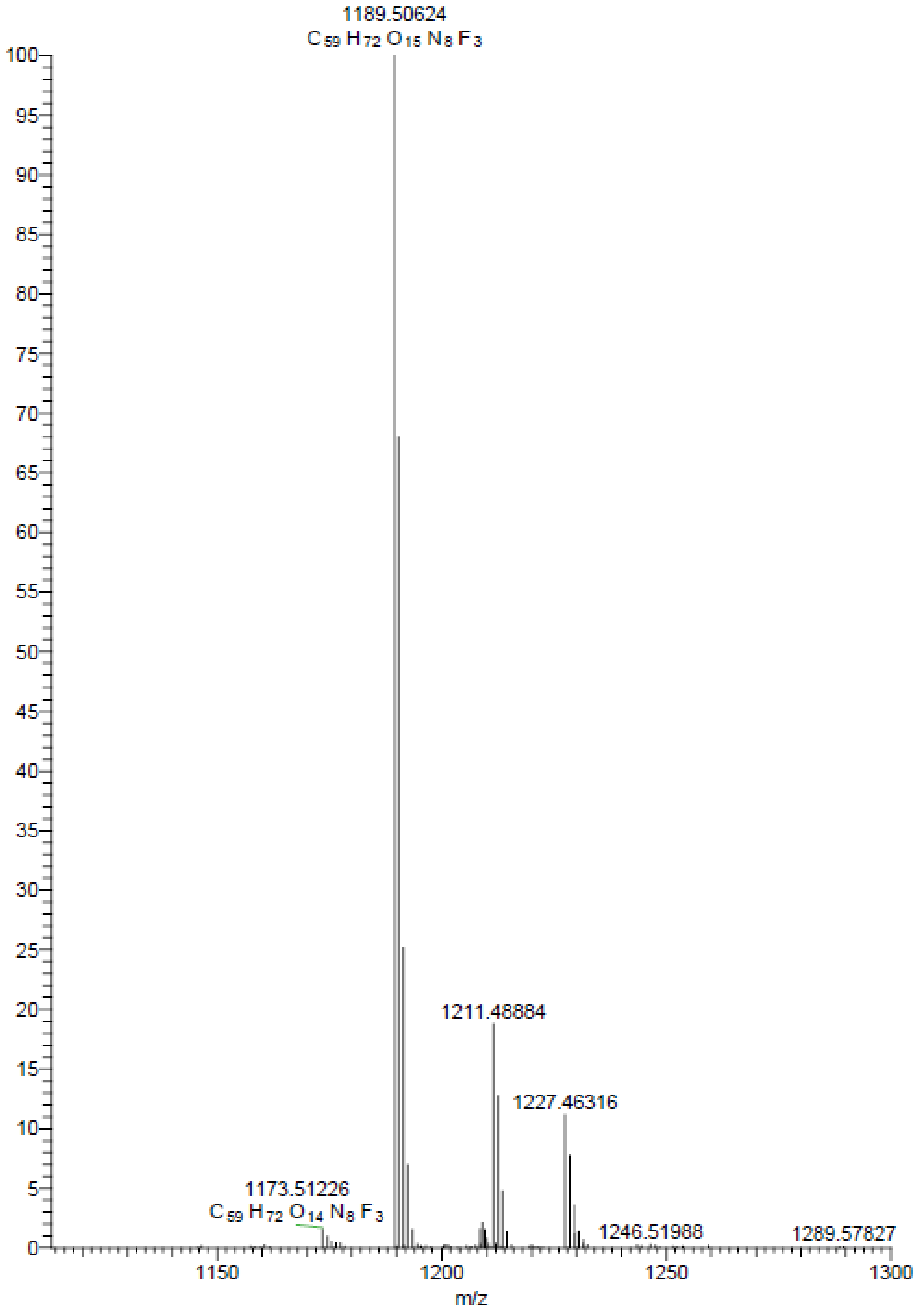

Elemental composition search on mass 1189.50624

m/z= 1184.50624-1194.50624

m/z	Theo. Mass	Delta (ppm)	RDB equiv.	Composition
1189.50624	1189.50637	-0.11	26.5	$C_{59}H_{72}O_{15}N_8F_3$

Compound **T1/2/T7**

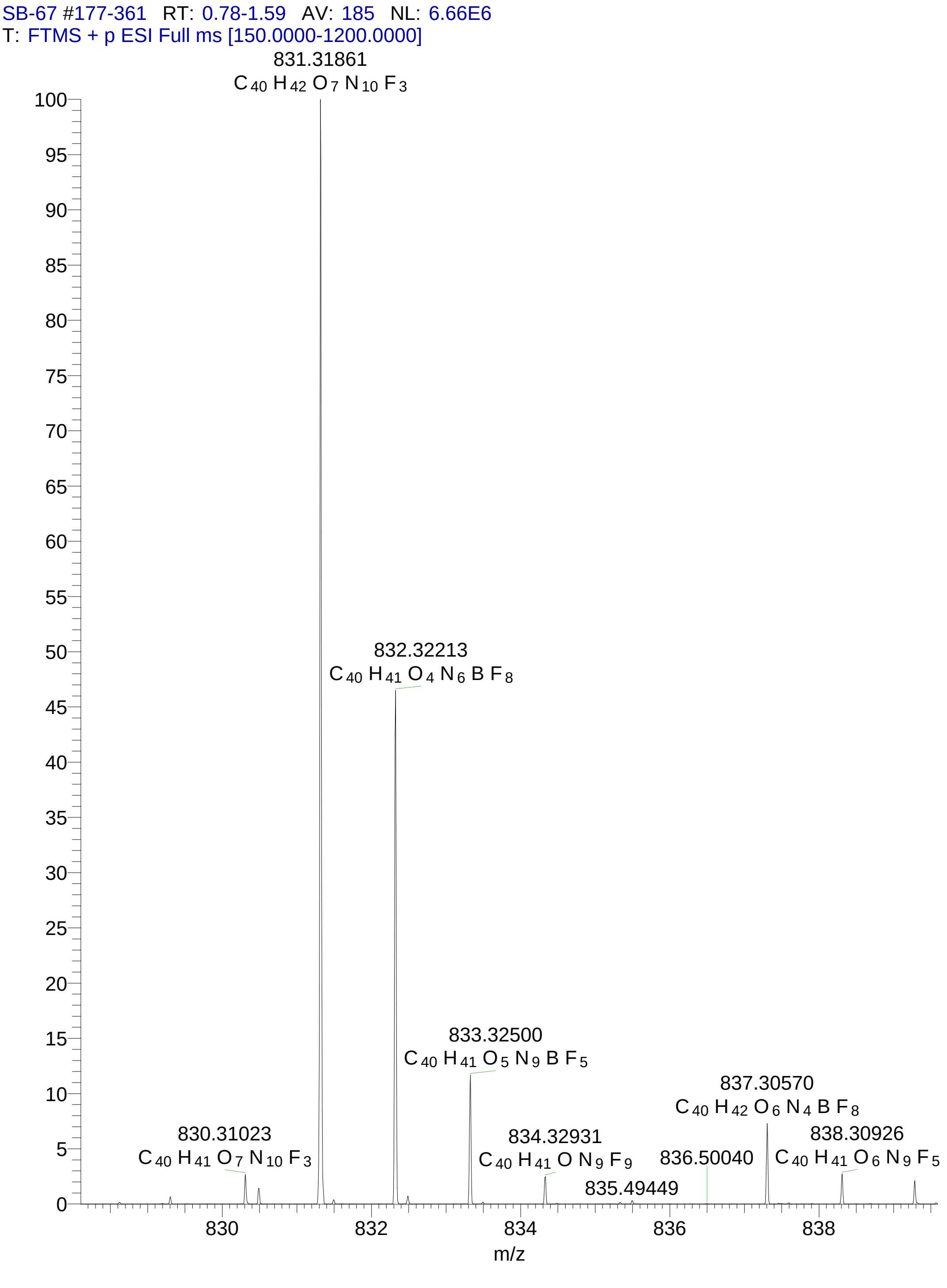

Elemental composition search on mass 831.31861

m/z= 826.31861-836.31861

m/z	Theo. Mass	Delta (ppm)	RDB equiv.	Composition
831.3186	831.3185	0.19	23.5	$C_{40}H_{42}O_7N_{10}F_3$
	[illegible]	[illegible]	[illegible]	[illegible]

Compound **T7/RI**

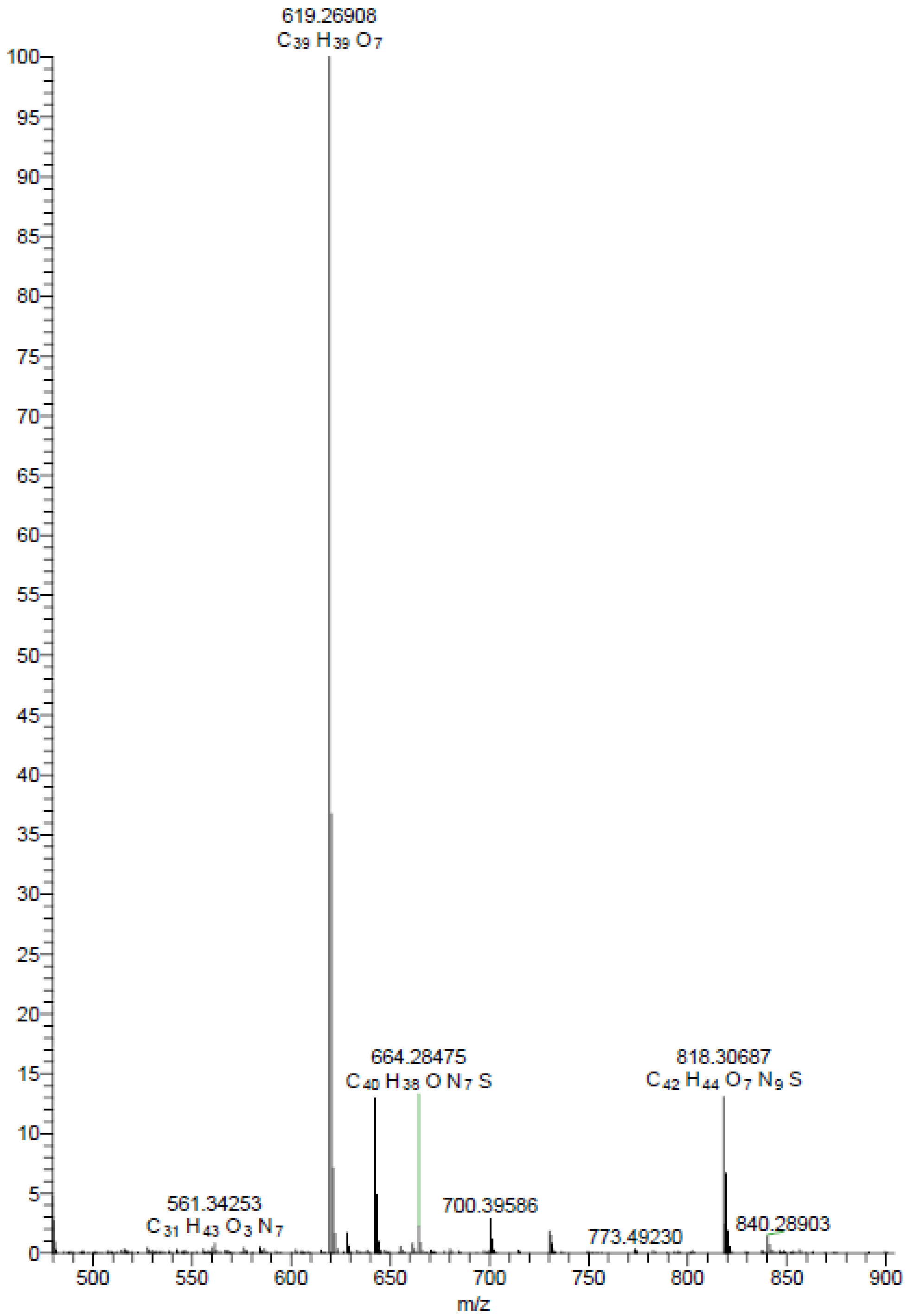

Elemental composition search on mass 818.30687

m/z= 813.30687-823.30687

m/z	Theo. Mass	Delta (ppm)	RDB equiv.	Composition
818.30687	818.30789	-1.25	25.5	$C_{42}H_{44}O_7N_9S$

Compound **T4/RI**

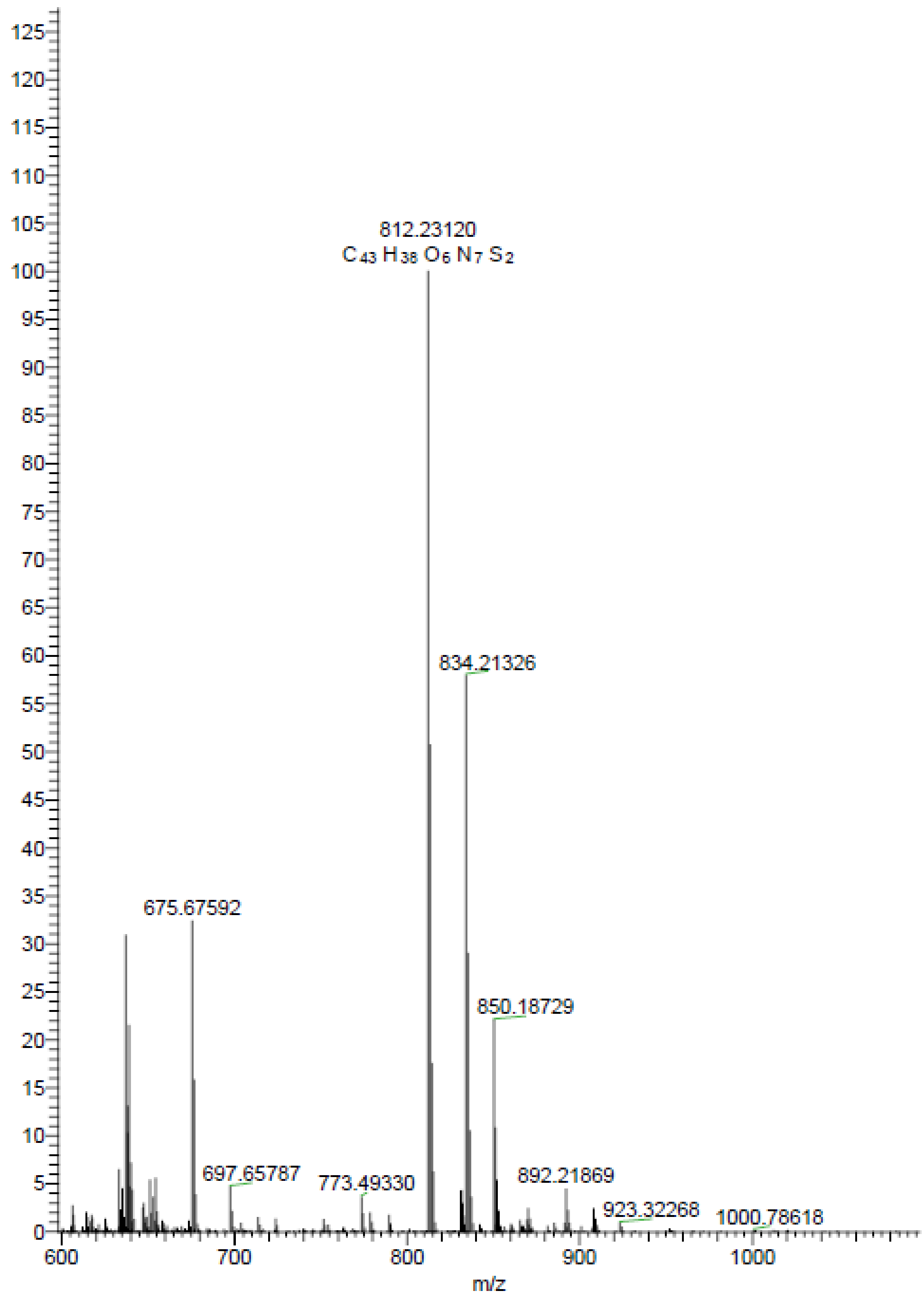

Elemental composition search on mass 812.23120

m/z= 807.23120-817.23120

m/z	Theo. Mass	Delta (ppm)	RDB equiv.	Composition
812.23120	812.23195	-0.92	28.5	$C_{43}H_{38}O_6N_7S_2$

Compound **T1/2/RI**

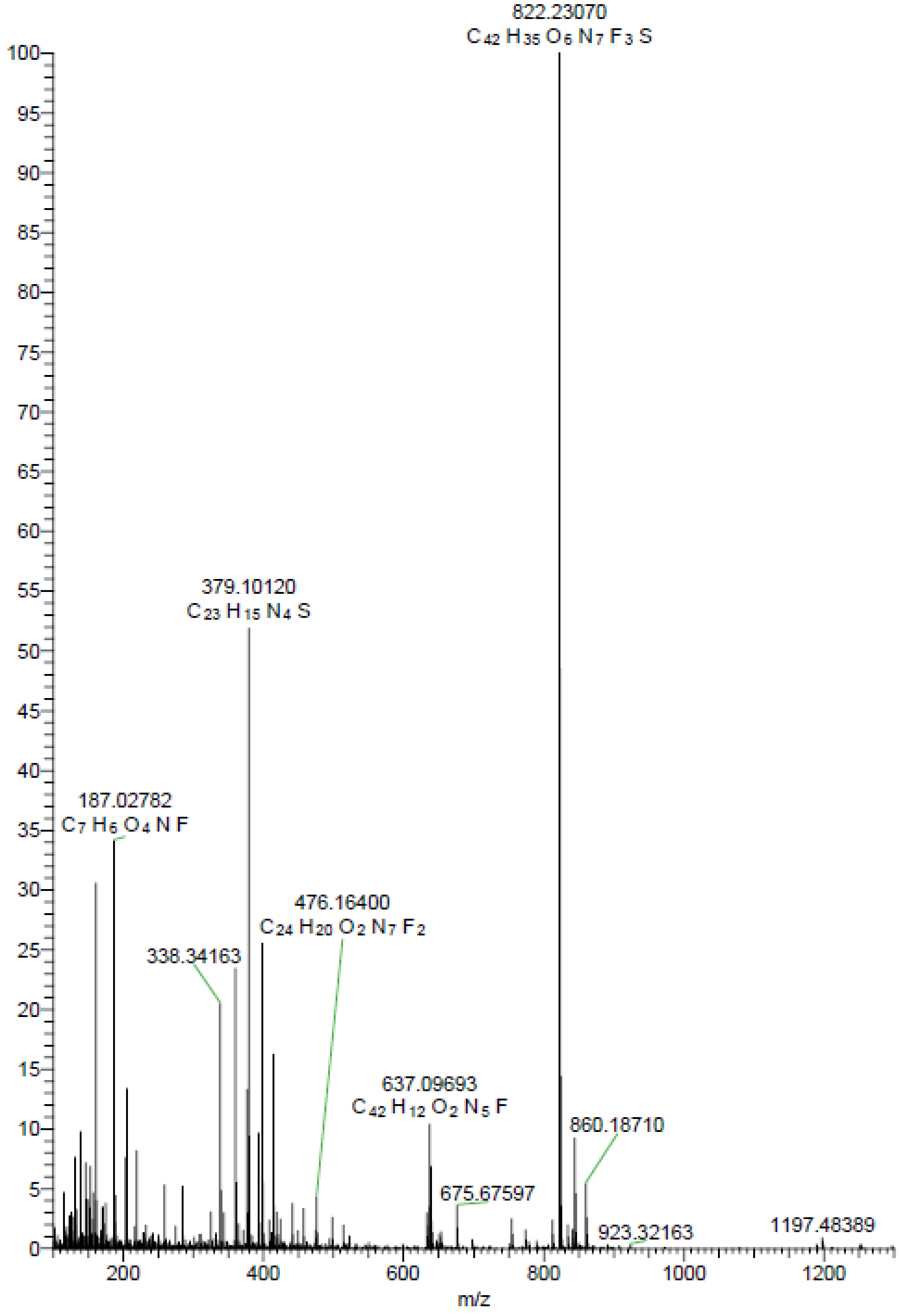

Elemental composition search on mass 822.23070

m/z= 817.23070-827.23070

m/z	Theo. Mass	Delta (ppm)	RDB equiv.	Composition
822.23070	822.23161	-1.11	27.5	$C_{42} H_{35} O_6 N_7 F_3 S$

Compound **N2/RI**

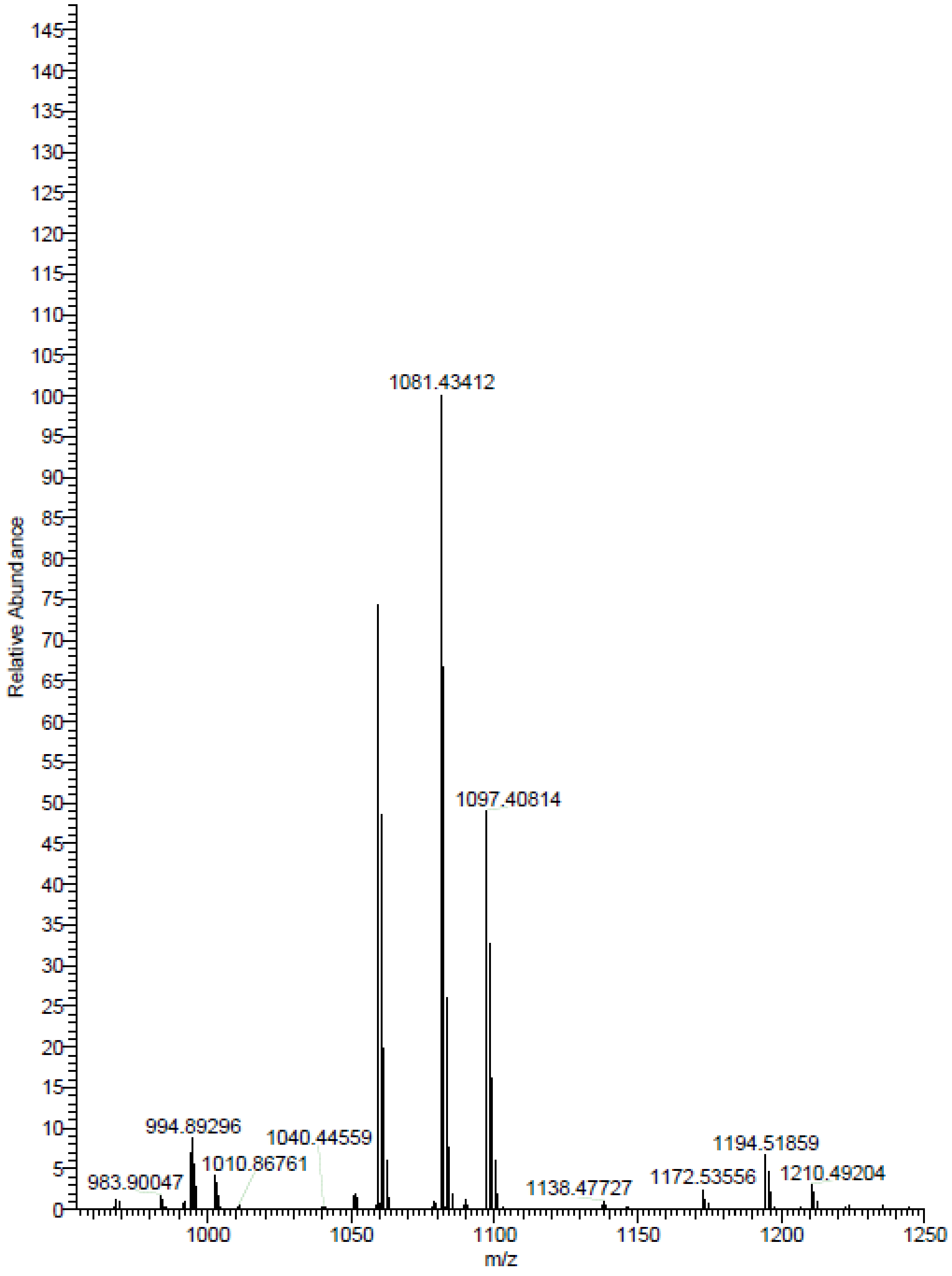

Elemental composition search on mass 1059.45239

m/z= 1054.45239-1064.45239

m/z	Theo. Mass	Delta (ppm)	RDB equiv.	Composition
1059.45239	1059.45322	-0.78	27.5	$C_{57}H_{67}O_{12}N_6S$

7. 1H and 13C NMR spectra of final compounds

Compound **T4/T7:** ^{1}H, 400 MHz, DMSO-d_6

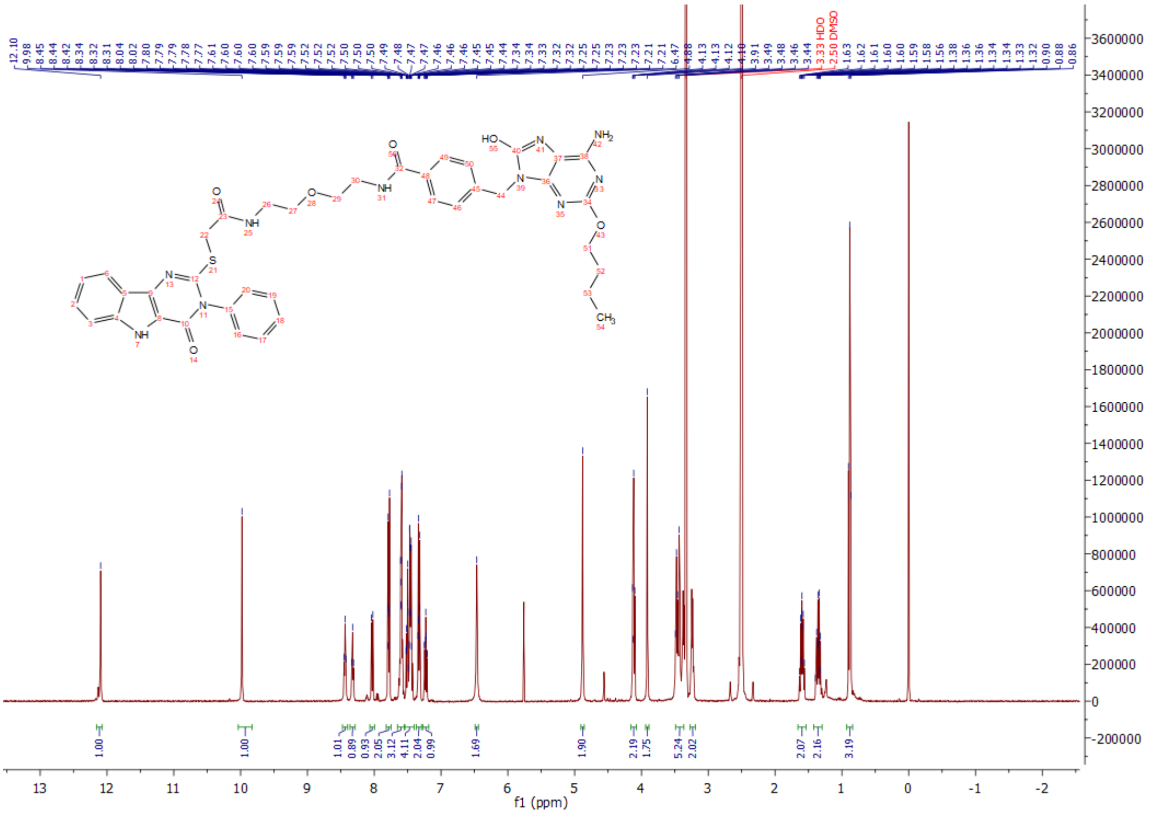

Compound **T4/T7:** ^{13}C, 100 MHz, DMSO-d_6

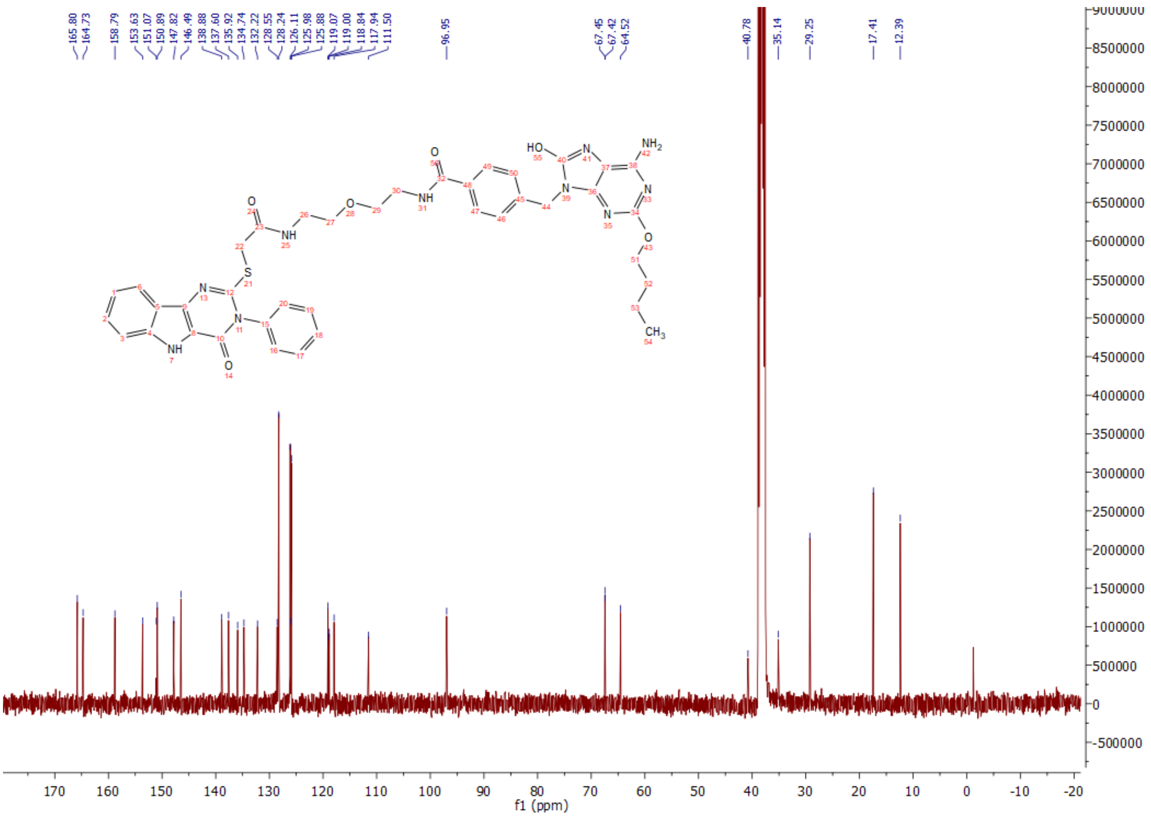

Compound **T4/N2:** ^{1}H, 400 MHz, $CDCl_3$

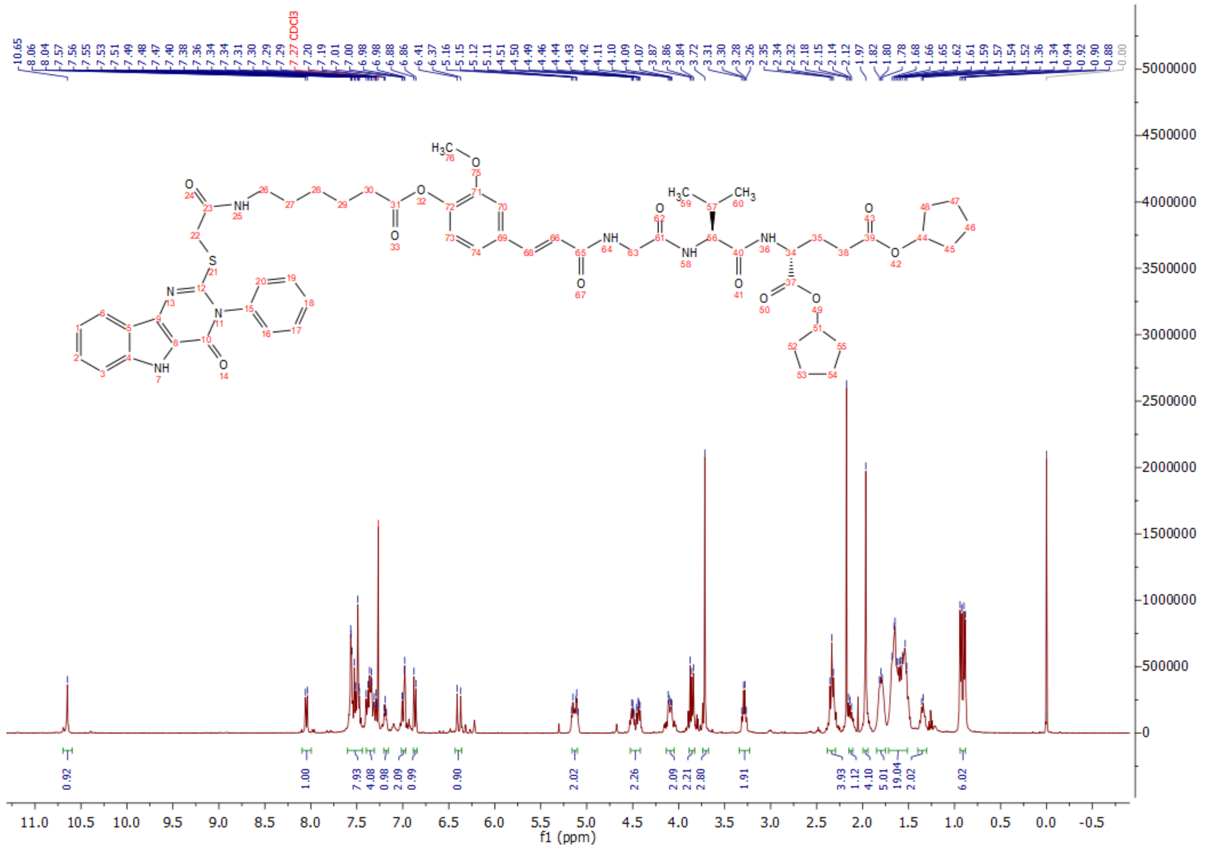

Compound **T4/N2:** ^{13}C, 100 MHz, $CDCl_3$

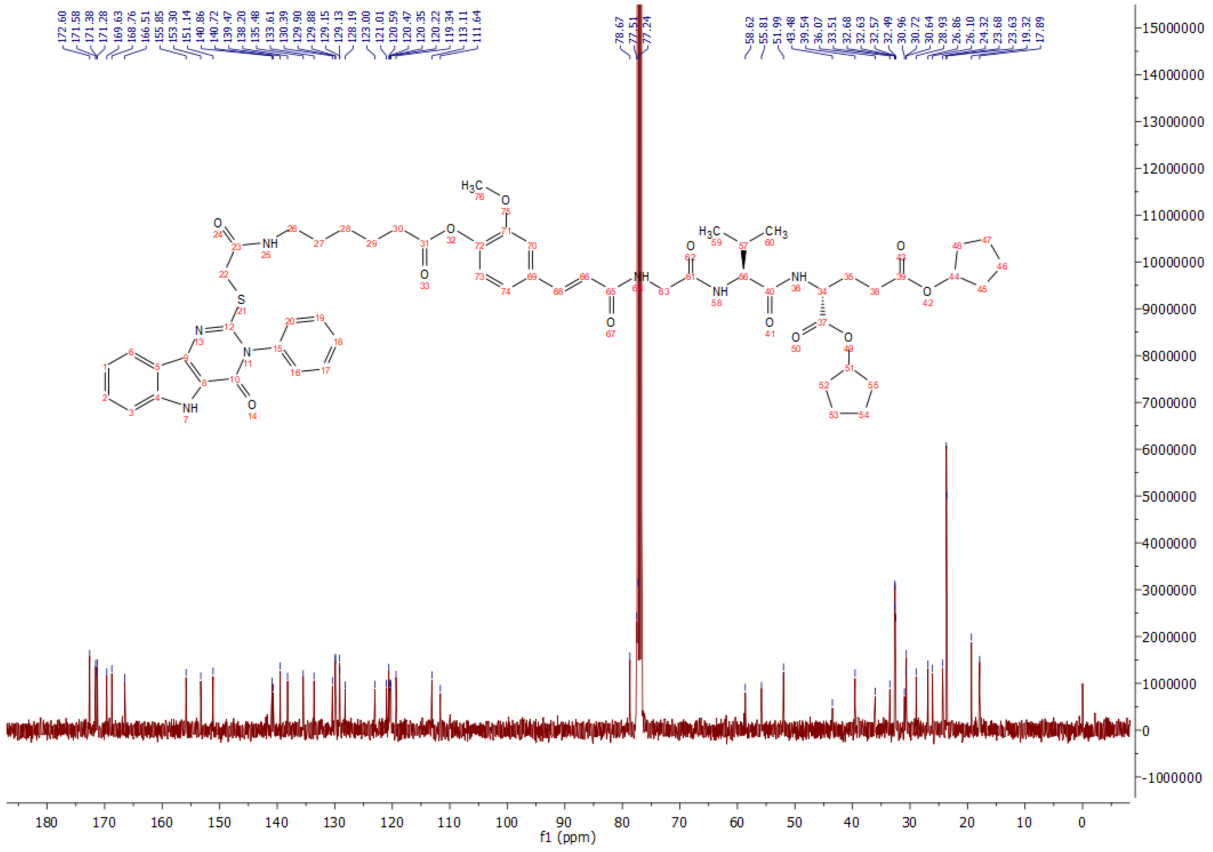

Compound **T1/2/T4:** ^{1}H, 400 MHz, DMSO-d_6

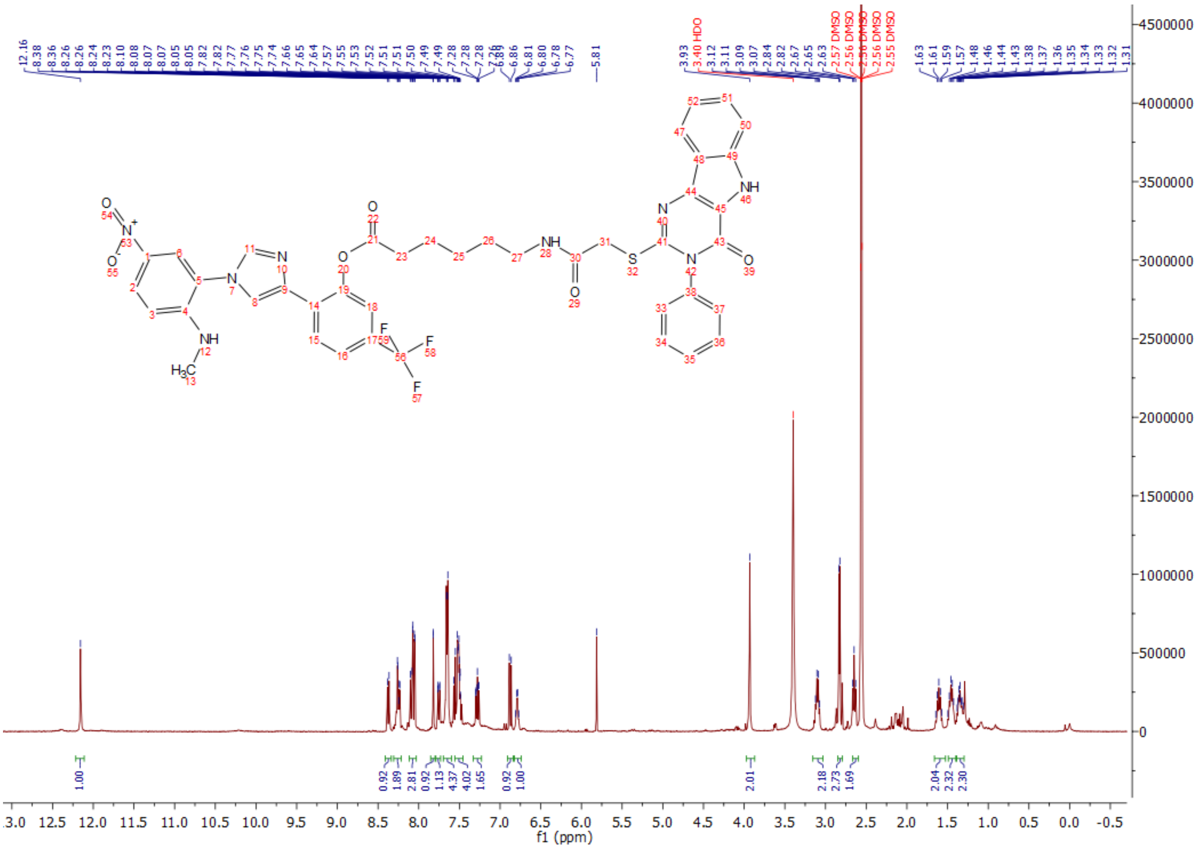

Compound **T1/2/T4:** ^{13}C, 100 MHz, DMSO-d_6

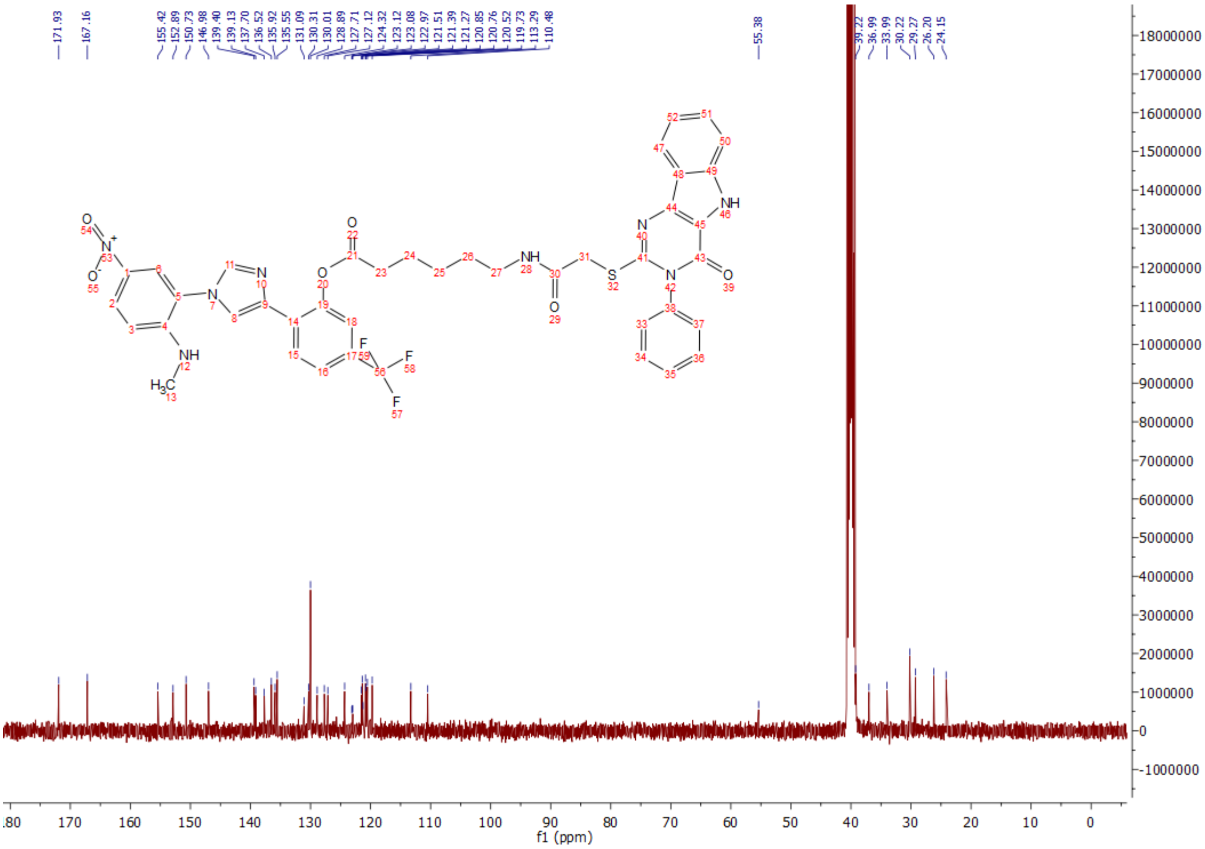

Compound **T1/2/N2:** ^{1}H, 400 MHz, DMSO-d_6

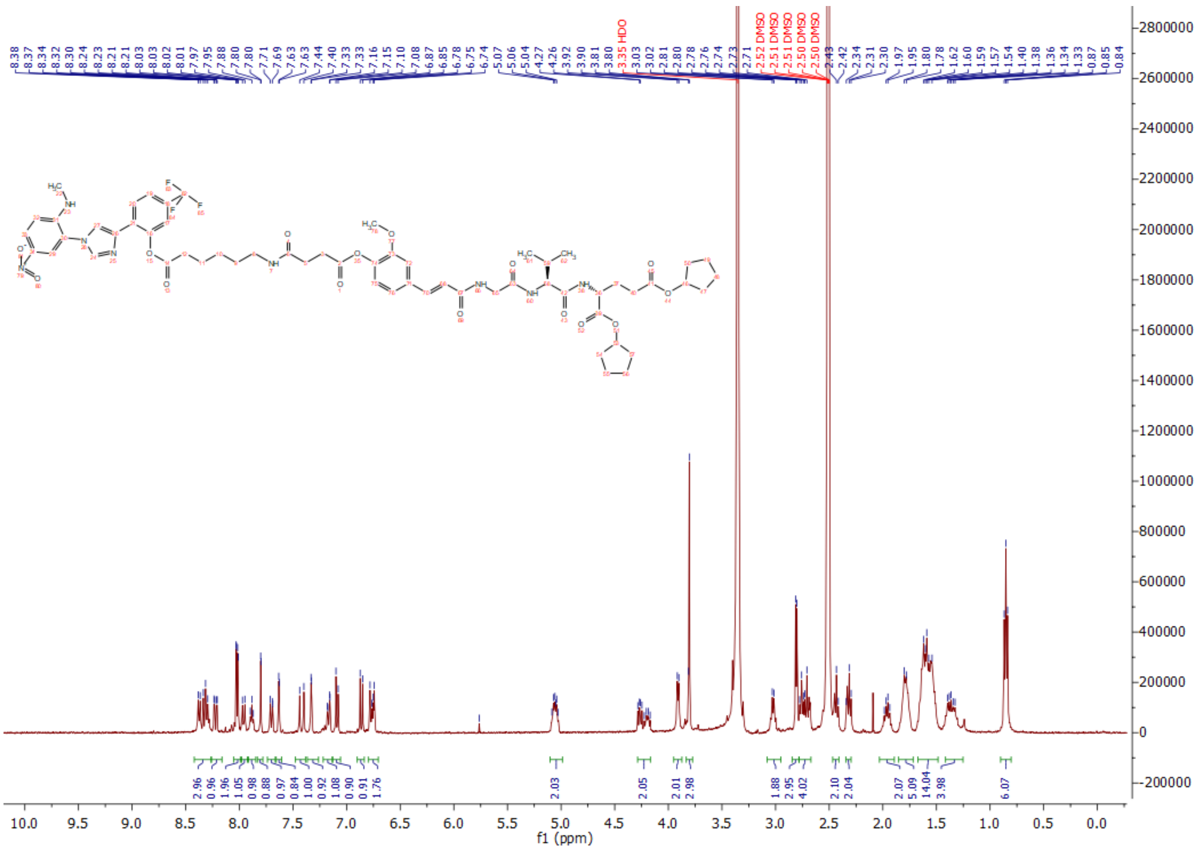

Compound **T1/2/N2:** ^{13}C, 100 MHz, DMSO-d_6

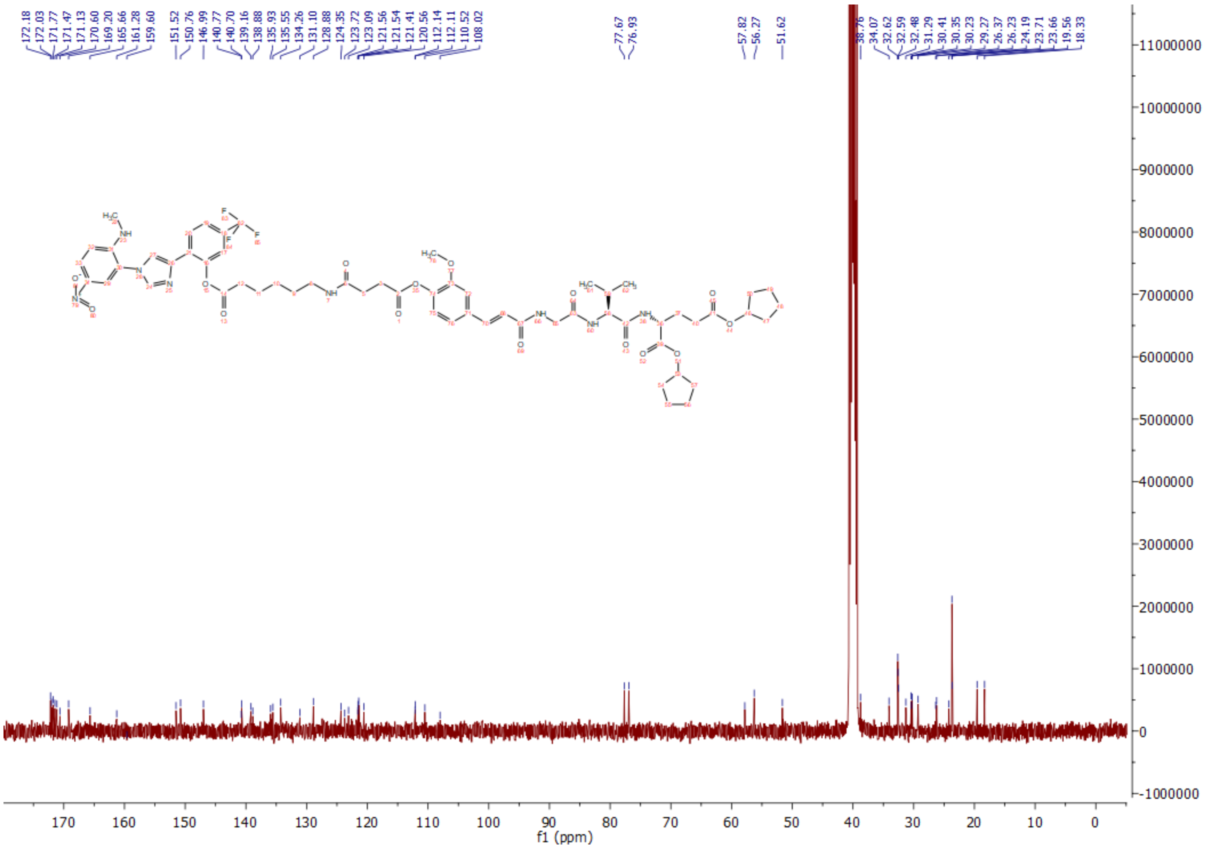

Compound **T1/2/T7:** ^{1}H, 400 MHz, DMSO-d$_6$

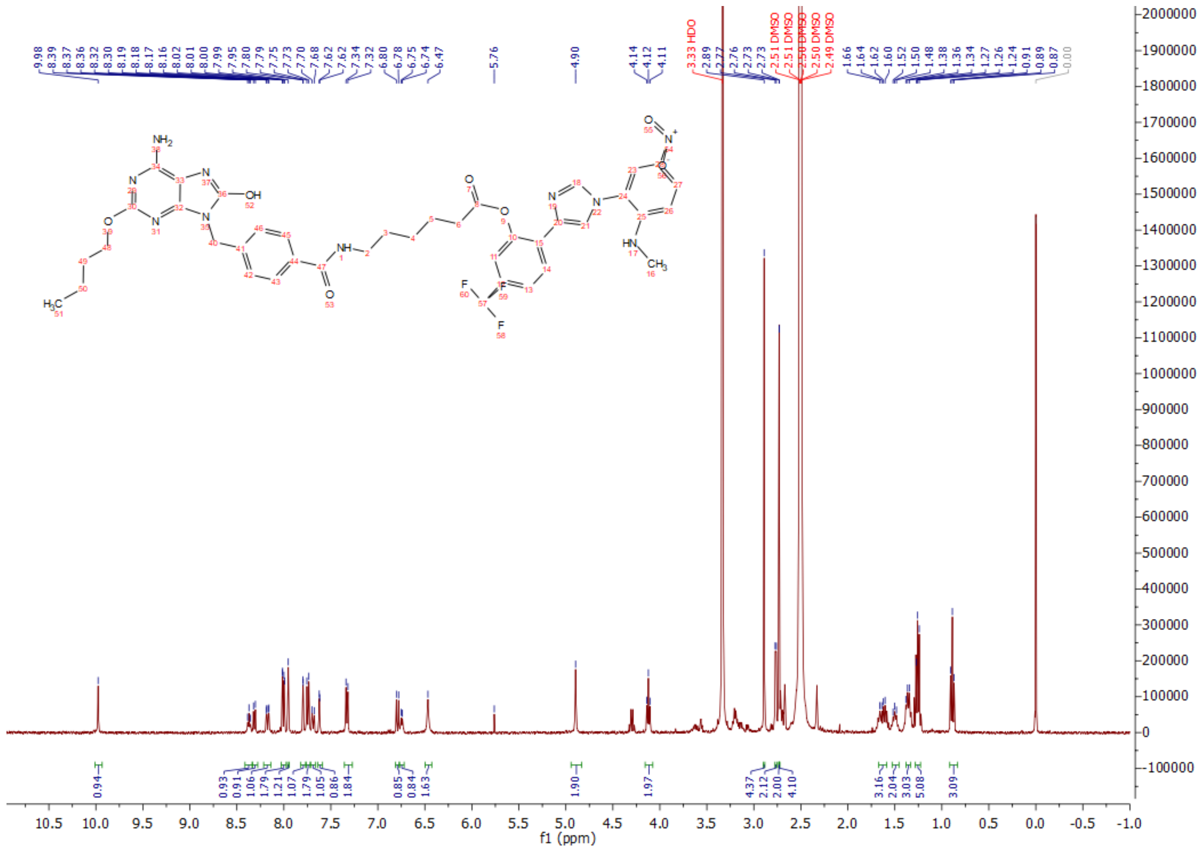

Compound **T1/2/T7:** ^{1}H, 100 MHz, DMSO-d$_6$

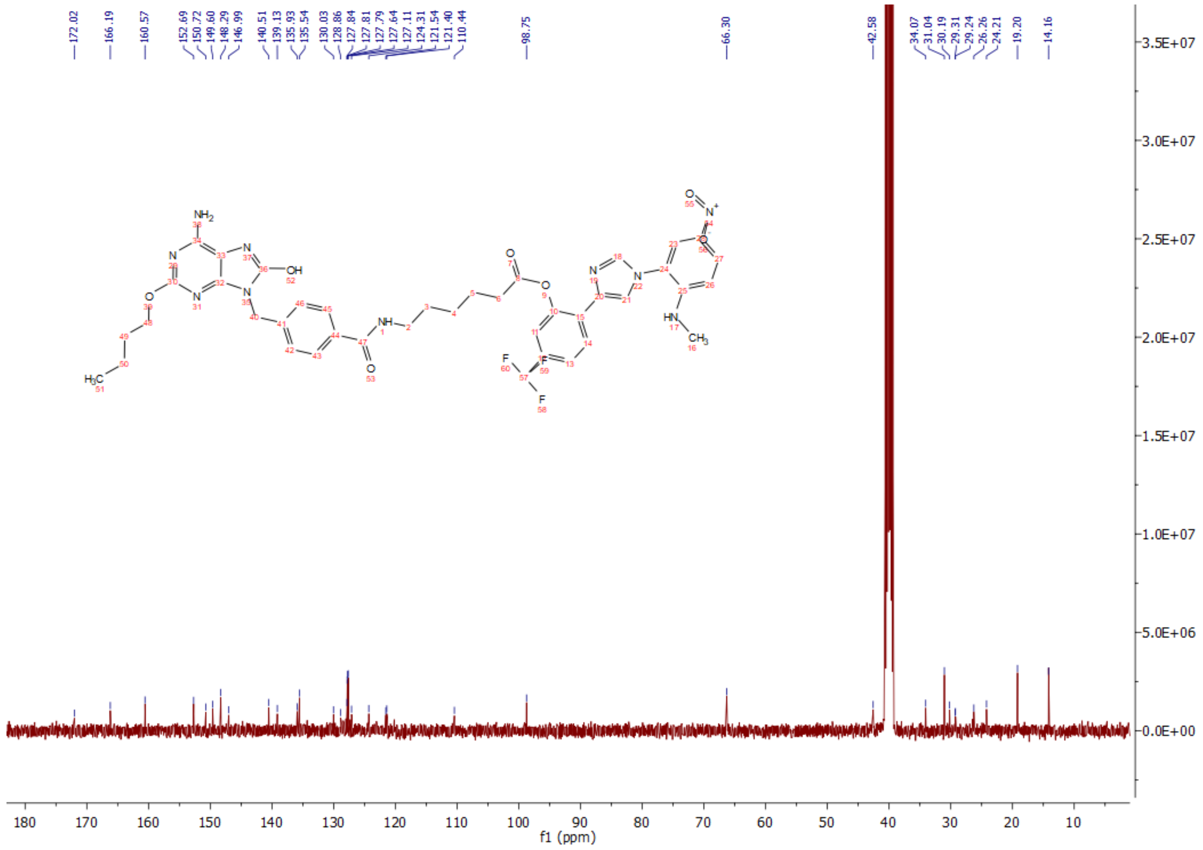

Compound **T7/RI**: ^{1}H, 400 MHz, DMSO-d_6

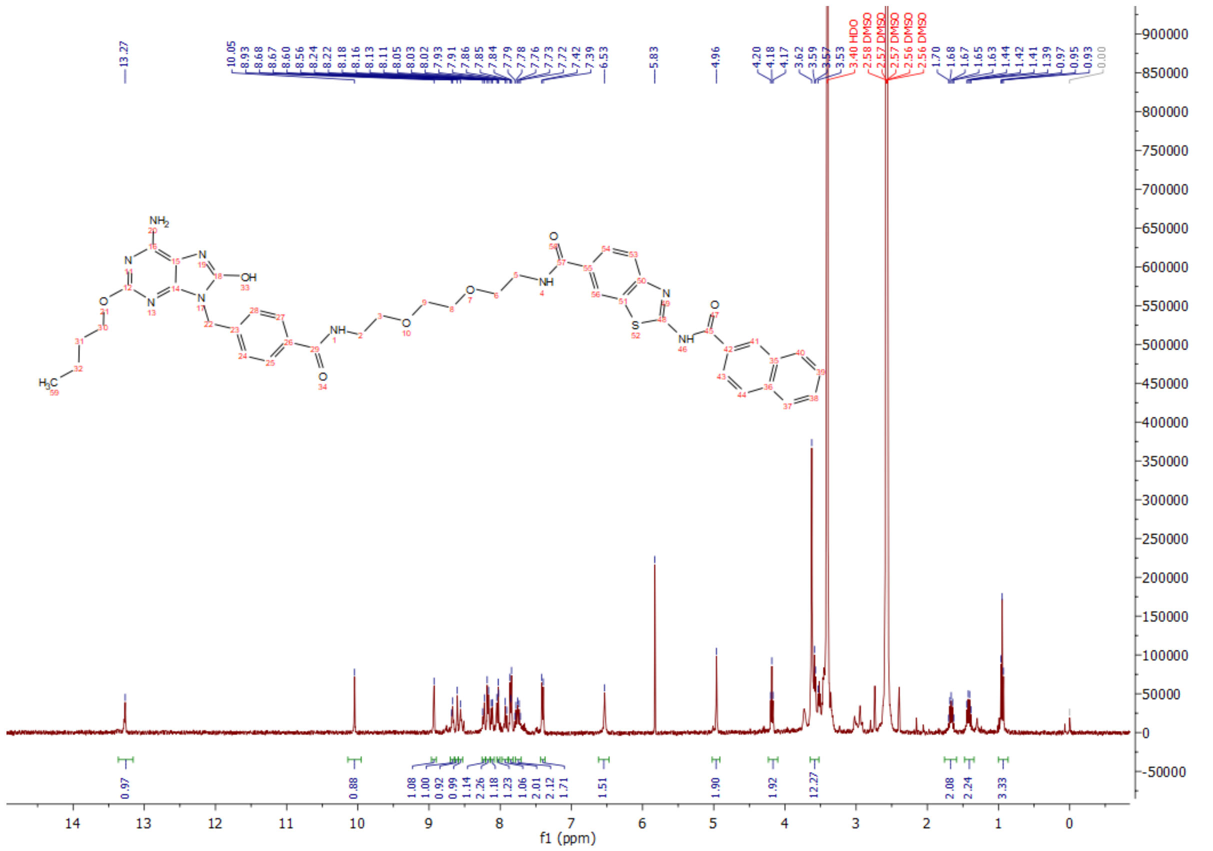

Compound **T7/RI**: ^{13}C, 100 MHz, DMSO-d_6

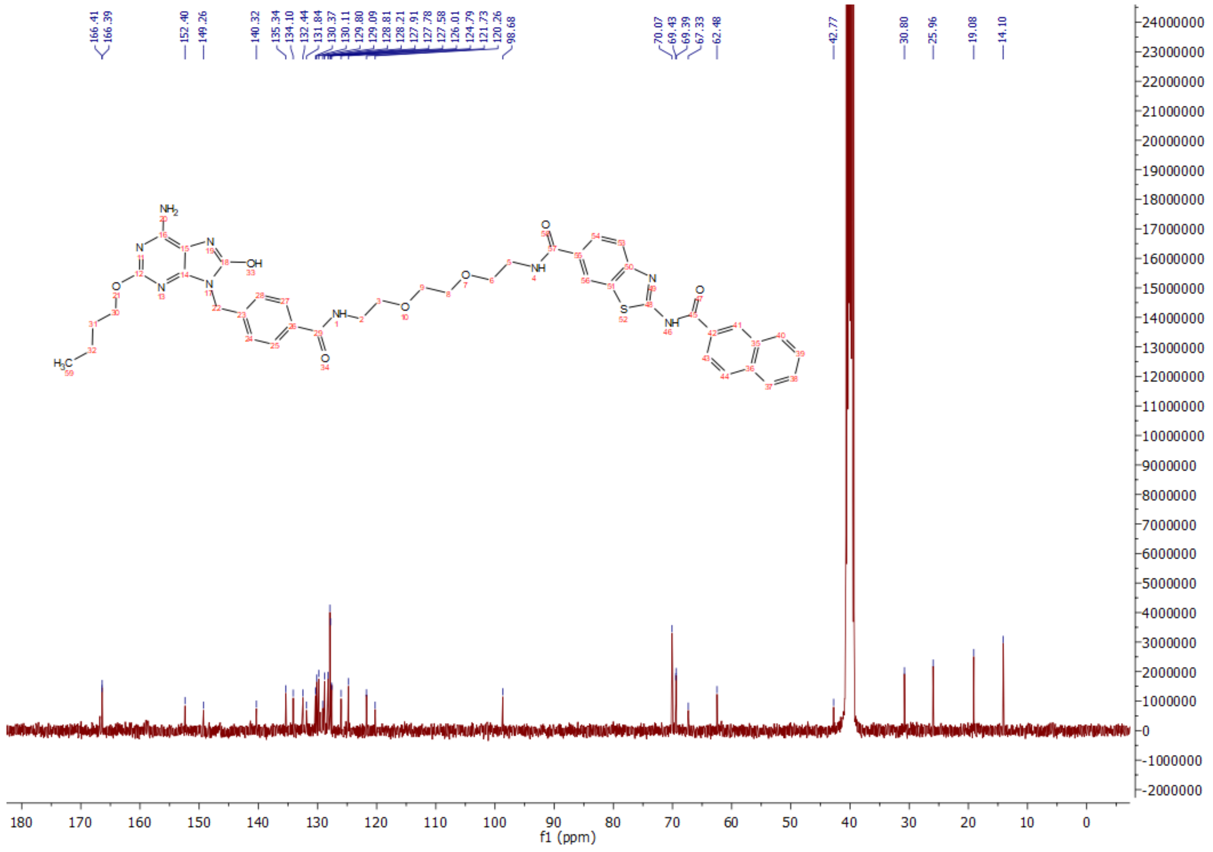

Compound **T4/RI**: ^{1}H, 400 MHz, DMSO-d$_6$

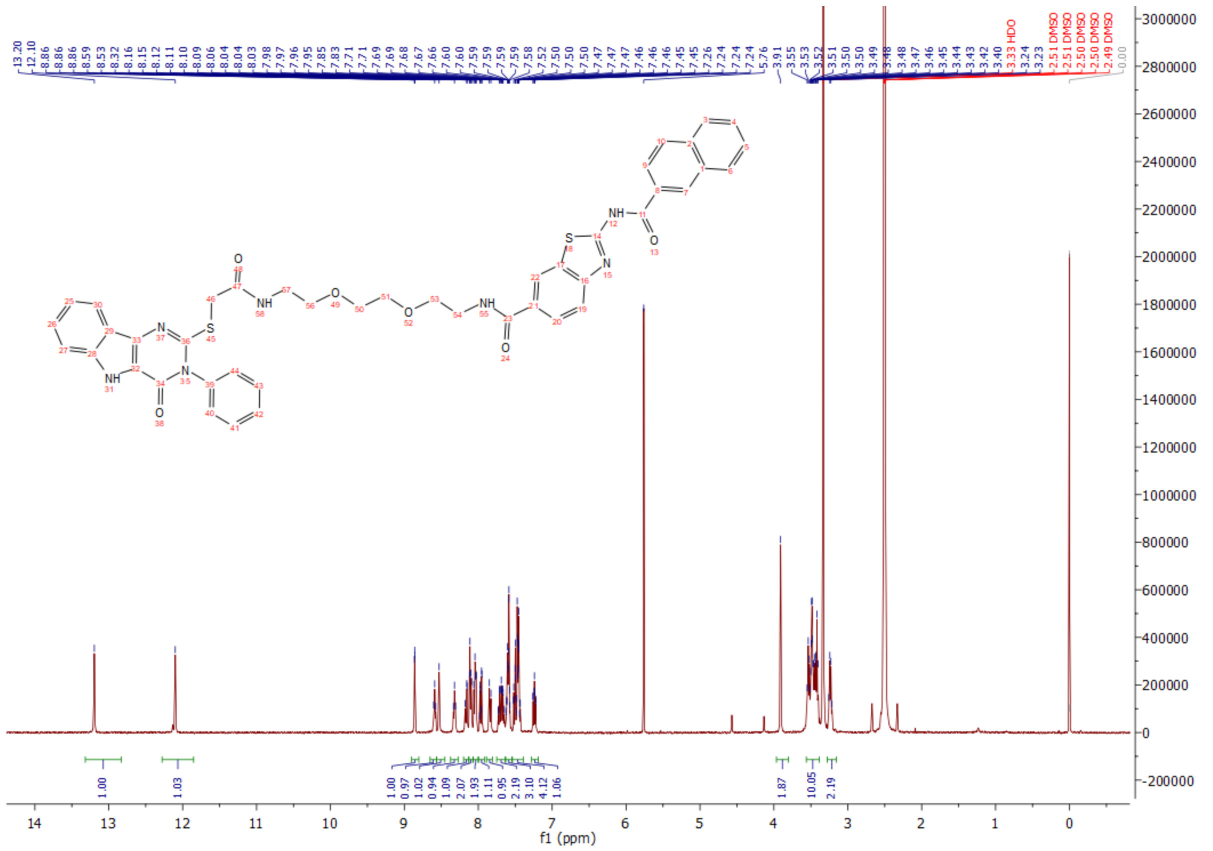

Compound **T4/RI**: ^{13}C, 100 MHz, DMSO-d$_6$

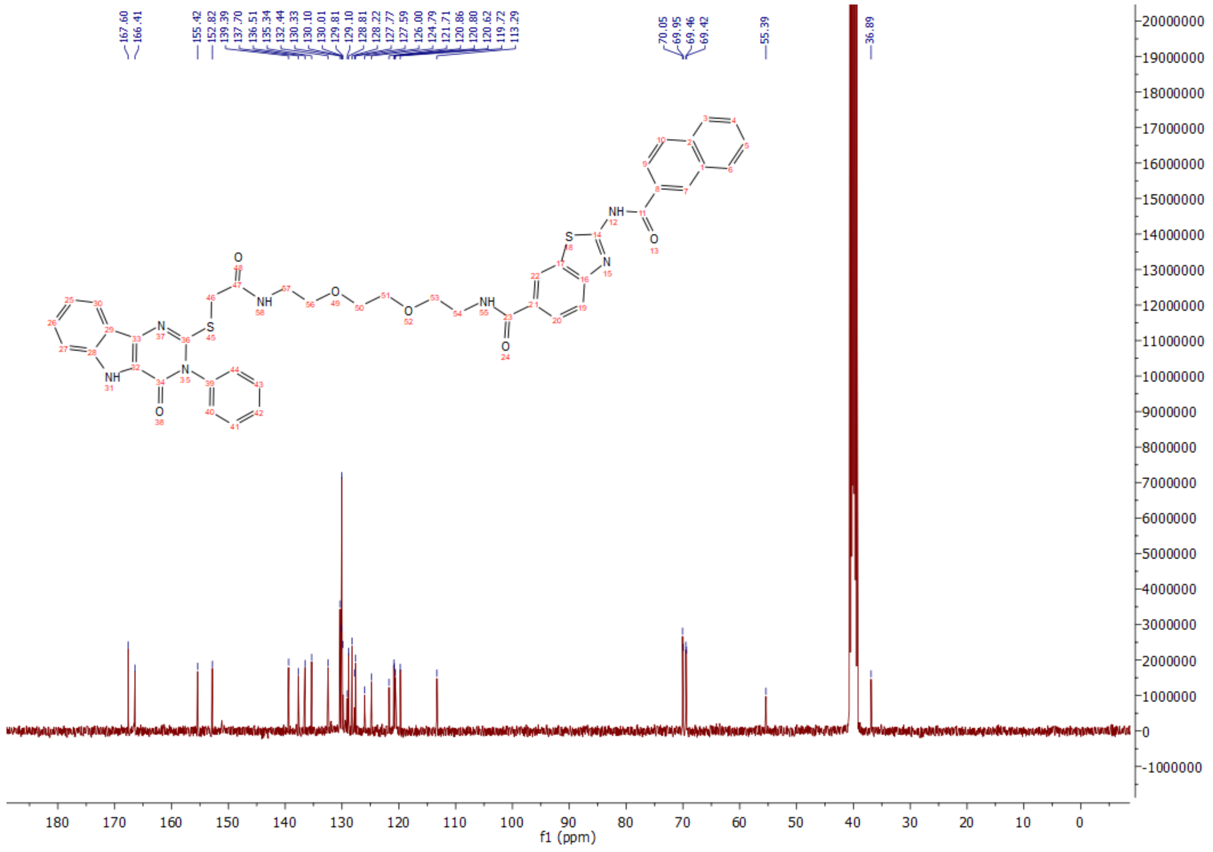

Compound **T1/2/RI**: ^{1}H, 400 MHz, DMSO-d$_6$

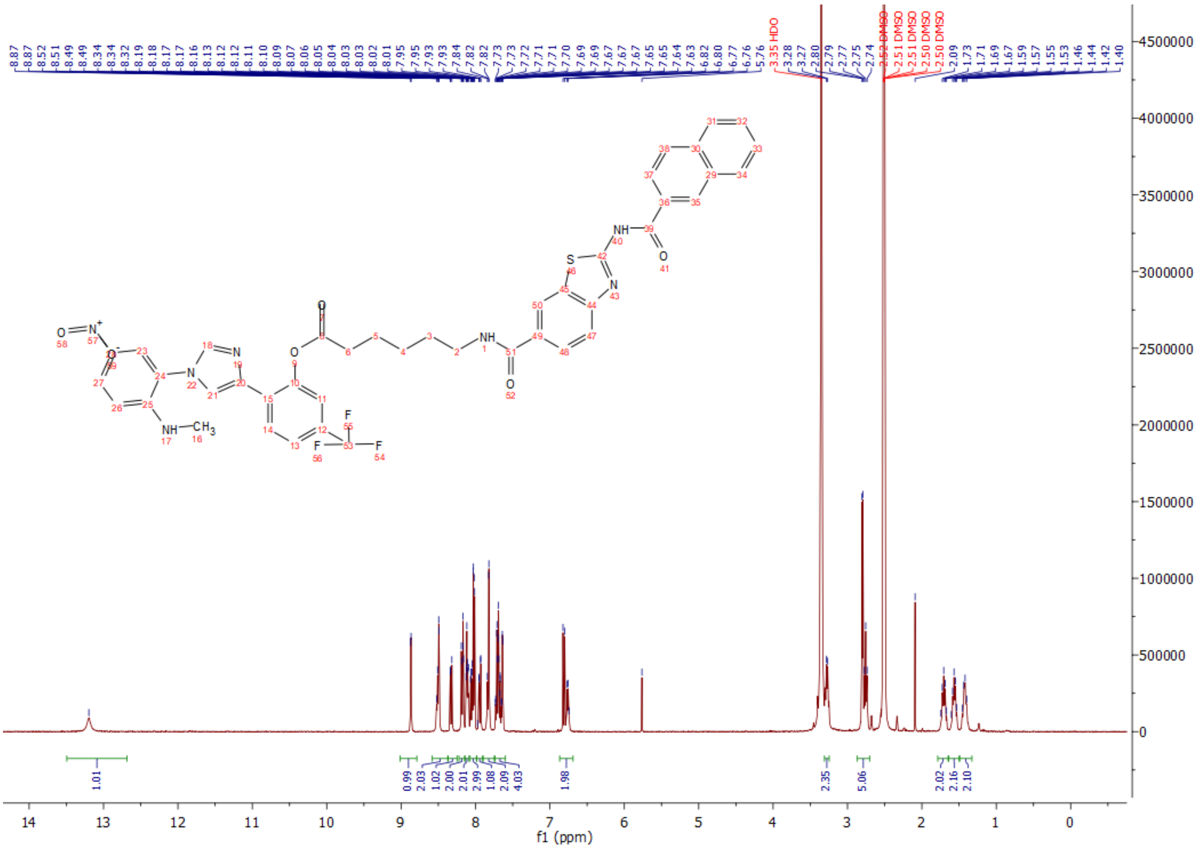

Compound **T1/2/RI**: ^{13}C, 100 MHz, DMSO-d$_6$

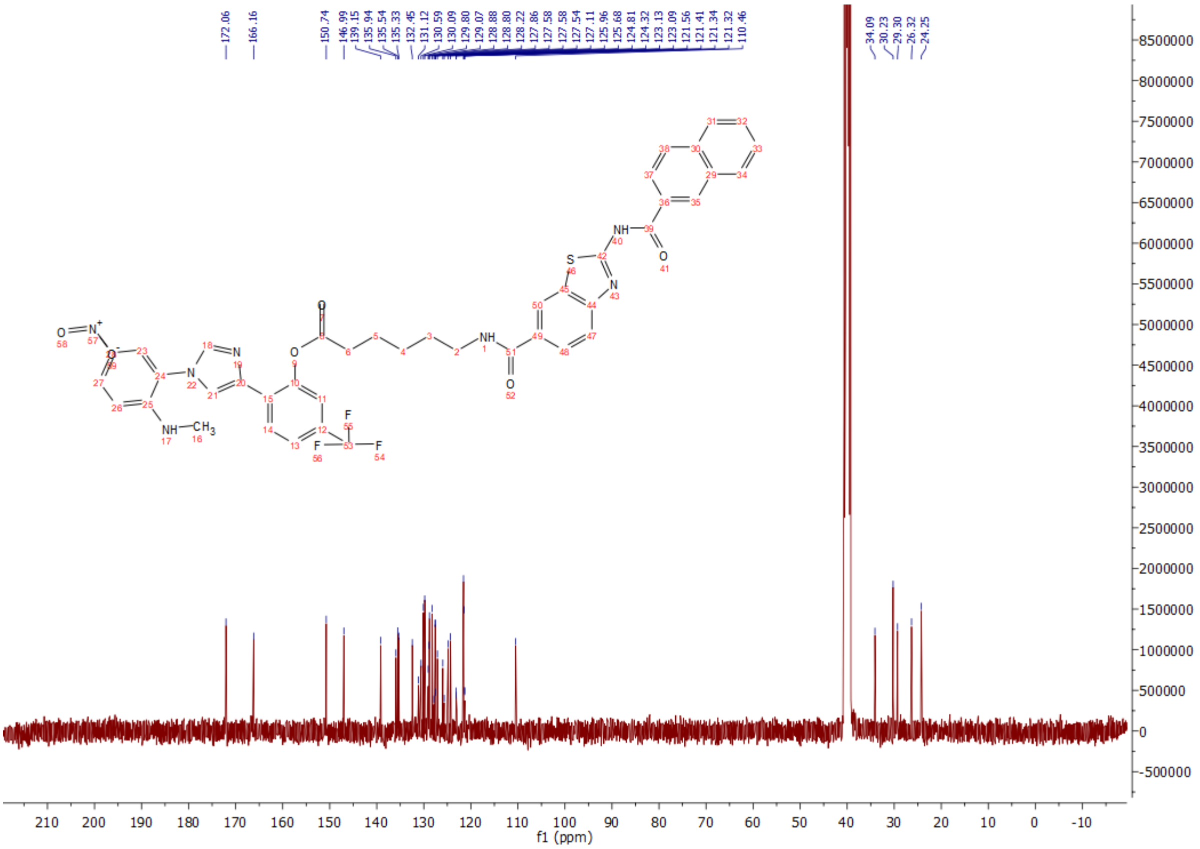

Compound **N2/RI**: ^{1}H, 400 MHz, DMSO-d$_6$

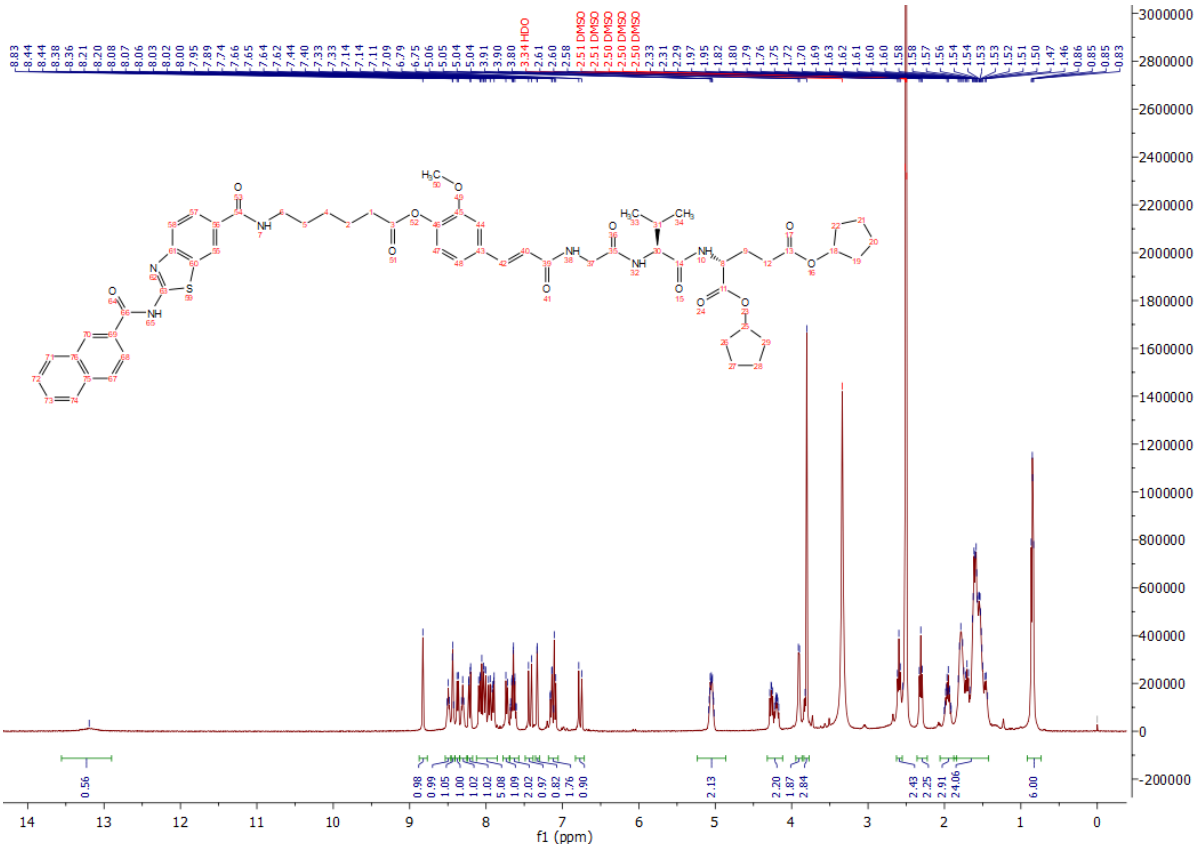

Compound **N2/RI**: ^{13}C, 100 MHz, DMSO-d$_6$

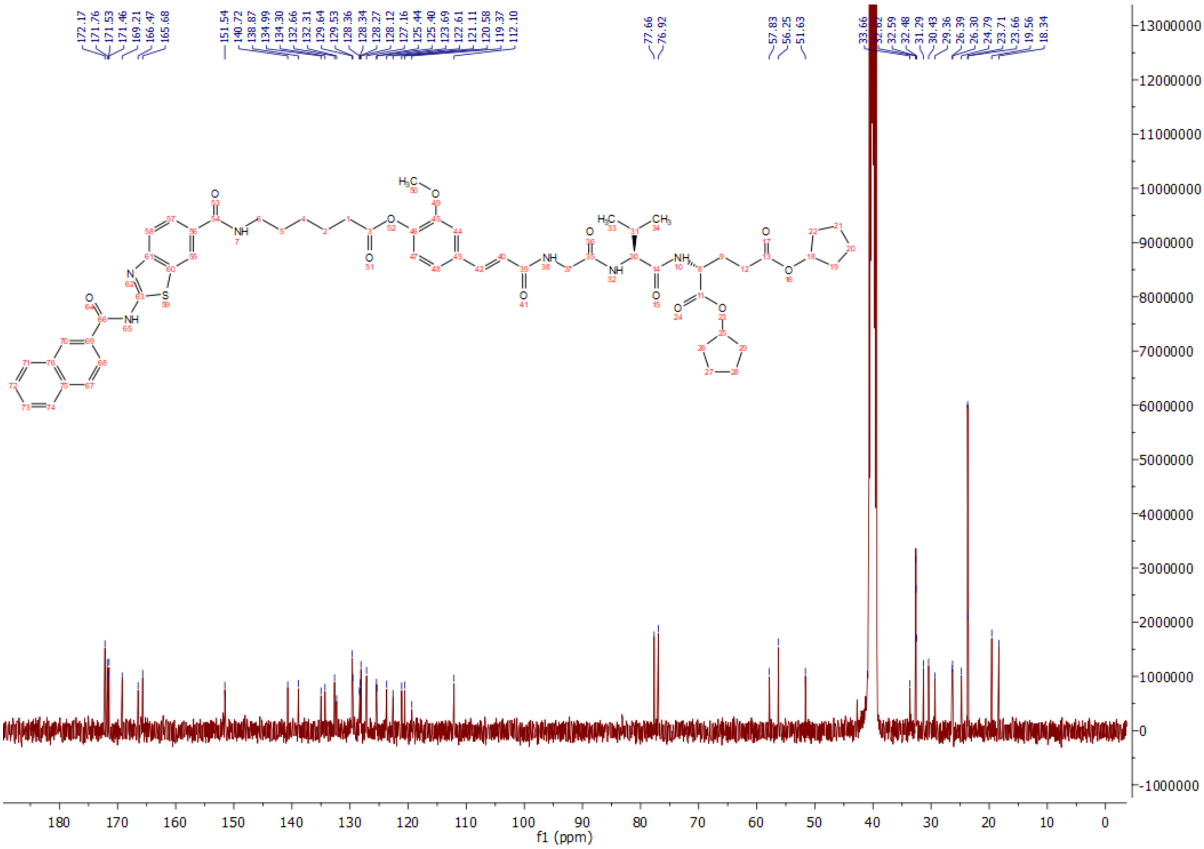

Supplement: Supplementary file 1 [file jm6c00372_si_001.pdf]
